# An overview of *Phoneutria nigriventer* spider venom using combined transcriptomic and proteomic approaches

**DOI:** 10.1371/journal.pone.0200628

**Published:** 2018-08-01

**Authors:** Marcelo R. V. Diniz, Ana L. B. Paiva, Clara Guerra-Duarte, Milton Y. Nishiyama, Mauricio A. Mudadu, Ursula de Oliveira, Márcia H. Borges, John R. Yates, Inácio de L. Junqueira-de-Azevedo

**Affiliations:** 1 Laboratório de Toxinologia Molecular, Diretoria de Pesquisa e Desenvolvimento, Fundação Ezequiel Dias, Belo Horizonte, Minas Gerais, Brazil; 2 Laboratório Especial de Toxinologia Aplicada, CeTICS, Instituto Butantan, São Paulo, SP, Brazil; 3 Embrapa Informática Agropecuária, Campinas, SP, Brazil; 4 Department of Chemical Physiology and Molecular and Cellular Neurobiology, The Scripps Research Institute, La Jolla, California, United States of America; Weizmann Institute of Science, ISRAEL

## Abstract

*Phoneutria nigriventer* is one of the largest existing true spiders and one of the few considered medically relevant. Its venom contains several neurotoxic peptides that act on different ion channels and chemical receptors of vertebrates and invertebrates. Some of these venom toxins have been shown as promising models for pharmaceutical or biotechnological use. However, the large diversity and the predominance of low molecular weight toxins in this venom have hampered the identification and deep investigation of the less abundant toxins and the proteins with high molecular weight. Here, we combined conventional and next-generation cDNA sequencing with Multidimensional Protein Identification Technology (MudPIT), to obtain an in-depth panorama of the composition of *P*. *nigriventer* spider venom. The results from these three approaches showed that cysteine-rich peptide toxins are the most abundant components in this venom and most of them contain the Inhibitor Cysteine Knot (ICK) structural motif. Ninety-eight sequences corresponding to cysteine-rich peptide toxins were identified by the three methodologies and many of them were considered as putative novel toxins, due to the low similarity to previously described toxins. Furthermore, using next-generation sequencing we identified families of several other classes of toxins, including CAPs (Cysteine Rich Secretory Protein—CRiSP, antigen 5 and Pathogenesis-Related 1—PR-1), serine proteinases, TCTPs (translationally controlled tumor proteins), proteinase inhibitors, metalloproteinases and hyaluronidases, which have been poorly described for this venom. This study provides an overview of the molecular diversity of *P*. *nigriventer* venom, revealing several novel components and providing a better basis to understand its toxicity and pharmacological activities.

## Introduction

The wandering spider *Phoneutria nigriventer* is a member of the Ctenidae family, infraorder Araneomorphae (true spiders). Together with other *Phoneutria* species, *P*. *nigriventer* is one of the largest existing true spiders [1], relying on its strength and venom toxicity for defense and prey capture. It belongs to the RTA (retrolateral tibial apophysis) clade, a diversified clade of modern spiders (~22,000 species) in which most members stopped using silk capture [2].

*P*. *nigriventer* is one of the few spiders considered medically relevant for human health [3]. The symptoms following *Phoneutria*’s bite (Phoneutrism) reveal the neurotoxic properties of its venom. The most frequent symptom is immediate local pain, usually of high intensity. Edema, erythema, sudoresis, paresthesia and muscle fasciculation may also occur at the bite site. In addition to local manifestations, tachycardia, hypertension, agitation, vomiting and sialorrhea are indications of systemic effects. In severe cases, which usually occur in children, profuse vomiting, priapism, diarrhea, bradycardia, hypotension, cardiac arrhythmia, acute pulmonary edema and shock have been described [4].

*P*. *nigriventer* venom is a complex mixture of enzymes, proteinaceous and non-proteinaceous neurotoxins [5], which act on ion channels (sodium, calcium and potassium) and chemical receptors of vertebrate and invertebrate neuromuscular systems (review in:[6]). Several of these venom toxins have been shown as promising models for pharmaceutical and biotechnological applications, with specific effects, such as penile erection [7], neuronal protection, cell death decrease after induced ischemia in hippocampal and retinal tissues [8,9], anti-arrhythmogenic effect on isolated heart and ventricular myocytes [10], antinociceptive effects in mice and rats [11–14] and insecticidal action [15,16], among others.

However, although much of the active proteinaceous components of *P*. *nigriventer* venom have already been characterized, there are still many other bioactive molecules that have not yet been explored and some remain undiscovered. Up to December 2017, UniProt database contained fifty-four molecules described for *P*. *nigriventer* venom, but this venom is speculated to comprise more than 150 proteinaceous molecules [17,18]. Most of the previous *P*. *nigriventer* venom characterization efforts have been driven and confined to studies on isolation and biological characterization of cysteine-rich peptide toxins with the Inhibitor Cysteine Knot (ICK) structural motif, first named as PnTxs (*Phoneutria nigriventer* toxins). The identification of high molecular weight proteins has been neglected, and only a few of them have been detected. Additionally, considering the large diversity and the predominance of PnTxs in this venom, including their isoforms, and considering that most of these toxins have similar molecular weight and isoeletric point, purification and analytical methods may not be efficient in detecting less abundant toxins.

High throughput methods for transcriptomic and proteomic analysis, combined with computational assembly and annotation of sequence data have allowed rapid characterization of protein components from spider venom glands [19–21]. In this work, we combined conventional and next-generation sequencing with Multidimensional protein identification technology (MudPIT) [22] proteomics to perform a large-scale omics investigation of *P*. *nigriventer* venom. Thus, this work presents the first overview of the molecular diversity of *P*. *nigriventer* venom, providing a better basis for understanding its toxicity and pharmacological activities.

## Material and methods

### Specimens

Venom glands and crude venom samples were obtained from adult specimens of *P*. *nigriventer* spiders maintained at Ezequiel Dias Foundation, Belo Horizonte, Brazil (CGEN license # 010815/2015-5).

### Next-generation sequencing (NGS) transcriptome

#### cDNA library preparation and sequencing

Venom glands from twenty adult female specimens of *P*. *nigriventer* were used to produce a cDNA library. Forty-eight hours after being milked by electrical stimulation, the venom glands were removed, dissected and immediately frozen at −80°C. Total RNA was extracted using TRIzol reagent (Ambion, Life Technologies). Total RNA integrity was assessed using an Agilent 2100 Bioanalyzer with the RNA 6000 Nano assay. mRNA was separated with magnetic beads with oligo (dT) using Dynabeads® mRNA DIRECT kit (Ambion, Life Technologies) and quantified by Quant-iT™ RiboGreen® RNA reagent and Kit (Invitrogen, Life Technologies Corp.). mRNA integrity was evaluated in a 2100 Bioanalyzer, picochip series (Agilent Technologies). A cDNA library was generated following the standard TruSeq RNA Sample Prep Kit protocol (Illumina, San Diego, CA). Briefly, cDNA was synthesized from fragmented mRNA using random hexamer primers, followed by ligation with appropriate sequencing adaptors. The size distribution of the cDNA libraries was measured with a 2100 Bioanalyzer using DNA1000 assay (Agilent Technologies). ABI StepOnePlus Real-Time PCR System was used for library sample quantification before sequencing. The cDNA library was sequenced on Illumina HiSeq 1500 System, in a Rapid Run mode in a 2-lane paired-end flowcell, run for 300 cycles, generating 2*151bp paired-end reads for each fragment, according to the standard manufacturer's protocol (Illumina).

#### RNA-Seq raw data pre-processing, *de novo* assembly and functional annotation

Using Illumina Casava software (version: 1.8.2), with Illumina quality control QC>Q30, a pair of paired-end “fastq” files was generated. RNA-Seq raw data reads were filtered to exclude PhiX internal control, using the software Bowtie2 version 2.2.3 [23]. Raw sequencing reads were pre-processed by an “in house” pipeline for sequencing quality control, to trim and remove low-complexity reads and homopolymer-enriched regions, poly-A/T/N tails, adapter sequences and low-quality bases, using the software programs fastq-mcf 1.04.662 [24] and Bowtie 2 2.2.3 [23]. The reads were filtered out when more than 90% of the sequence corresponded to homopolymers or low-complexity regions, and they were trimmed when the mean quality score was lower than 25 in a window size of 15. After trimming, all reads smaller than 40 bp were discarded. To generate a non-redundant set of unique sequence transcripts, we performed *de novo* assembly with Trinity software [25], with the CuffFly parameter to reduce the number of false-positive isoforms. In order to estimate the transcript abundance we aligned each set of reads back to the *P*. *nigrivinter* assembled transcriptome and maximum likelihood abundance estimates were obtained using the RSEM method [26]. Assembled unique sequences with sequence length lower than 300 bp, unique sequences classified as putative contaminants based on UniVec database (NCBI) and those lowly expressed (FPKM<1) were filtered out. The completeness of the transcriptome was estimated by the presence of ultra-conserved eukaryotic protein sequences, tested with CEGMA pipeline [27] and BUSCO approaches [28].

*P*. *nigriventer* transcriptome was annotated using the BLASTx search and alignment tool with cut-off e-value <1e-5, against multiple protein databases: UniProt-Swissprot database [29], NCBI Transcriptome Shotgun Assembly protein database (TSA), UniProt Animal Toxin Annotation Project (www.UniProt.org/program/Toxins) and Animal Toxin Database (ATDB) [30]. InterProScan [31] was used to predict protein functional domains. The sequences were further annotated by Gene Ontology [32] using GO Slim, to give a broad overview of the ontology of the sequences obtained. A spreadsheet containing all cDNA unique sequences retrieved, their annotation against multiple databases and the correspondent FPKM was generated.

In order to determine putative venom components, a manual search of the spreadsheet containing the annotated unique sequences was performed, using terms corresponding to the annotation of previously identified toxins in venom gland transcriptomic analyses. The cDNA sequences retrieved were translated using the Translate tool from SIB ExPASy Bioformatics Resources Portal [33]. The protein sequences generated were used to perform a BlastP (protein-protein BLAST) search for similar proteins, against UniProtKB/Swiss-Prot database, to confirm their annotation. The SignalP 4.1 Server [34] was used to predict the presence and location of signal peptide cleavage sites in the amino acid sequences generated. For cysteine-rich peptide toxin sequences, the SpiderP algorithm, available in the Arachnoserver database [35], was used to identify putative propeptides.

Alignments of the assembled protein sequences with other previously identified toxins were performed with MUSCLE (MUltiple Sequence Comparison by Log- Expectation) multiple alignment tool [36] or Clustal Omega [37] for incomplete sequences. The identity percentage (ID%) of the unique sequences with reference proteins was calculated using EMBOSS Stretcher for pairwise sequence alignment [38].

### Conventional sequencing (CS) transcriptome

#### cDNA library construction and EST sequencing

Venom glands of ten adult female specimens of *P*. *nigriventer* were extracted and dissected 48 hours after milking and immediately stored at -80 C°. Total RNA was extracted with TRIzol reagent (Invitrogen, USA). mRNA purification was performed on an oligo(dT)- cellulose affinity column using mRNA Purification Kit (Pharmacia, Sweden). A cDNA library was constructed using Super Script Plasmid System with Gateway Technology for cDNA Synthesis and Cloning Kit (Invitrogen). cDNA fragments with selected sizes, ranging from 300 to 800 bp, were separated by agarose gel electrophoresis and cloned into the psPORT 1 vector (Invitrogen). Recombinant plasmids were used to transform *E*.*coli* DH5-α and random colonies were selected and cultured in Circle Grown medium (MP Biomedicals, USA) containing ampicillin (100μg/ml). After overnight culture, the plasmids were extracted by the alkaline lysis method [39] and were single-pass sequenced on ABI 3130 Sequencer using the standard M13 reverse primer and Big Dye terminator v3.1 Cycle sequencing kit (Applied Biosystems, USA).

#### Bioinformatics analyses and functional annotation

Expressed sequence tag (EST) sequences were edited using Cross-match and several in-house scripts created in Perl language to remove plasmid and sequence adapters, small sized sequences (cutoff 100 bases min), low-quality bases (window of 22 bases with mean quality value of 25) and poly A tails from the reads. TGICL [40] was used to cluster and assemble contigs. BLASTx was used to align sequences against UniProt-Swissprot database. Best hits were defined using E-value cutoff of <1e-5 and selecting the best score. “No match” sequences were checked for potential ORFs using the Getorf software from Emboss version 6.1.0, with the following flags: -minsize 150 (to search for ORFs with at least 150 nucleotides) and -find 1 (inside start and stop codons). SignalP 4.0 [34] and Prop 1.0c [41] were used to search for the presence of signal peptides and propeptides in all sequences.

### Proteomic analyses

#### Preparation of venom samples

*P*. *nigriventer* crude venom extract was obtained by electrical stimulation (voltage 7 V; Frequency 1 pulse per second) followed by milking of the spider’s chelicerae. The venom was processed for LC-MS analyses as previously described [42]. Briefly, the venom sample (100 μg) was solubilized in water (100 μL) and carefully precipitated with trichloroacetic acid (6.1N TCA, Sigma) to reach a final concentration of 25% (p/v) and kept overnight at 4^°^C. Later, the solution was centrifuged (14.000 *g* for 30 min at 4°C), the pellet was washed twice with ice-cold acetone (500 μL), centrifuged (14.000 *g* for 20 min at 4°C) and then air dried. The recovered pellet was solubilized in 100μL of 100 mM Tris-HCl, containing 8 M urea, pH 8.5. Disulfide-bridge cysteine residues were reduced by 1 M Tris (2-carboxyethyl) phosphine (TCEP, Sigma Aldrich) to a final concentration of 5 mM and incubated at room temperature for 20 min. For alkylation of thiol groups, 500 mM iodoacetamide to a final concentration of 10 mM was added, and the solution was incubated at room temperature for 30 min in the dark. Trypsin (Promega) was added to a ratio of 1:50 (enzyme to substrate wt/wt) and incubated at 37 ^o^C overnight in the dark. Digestion was stopped by adding 90% formic acid to a final concentration of 5% (v/v) followed by centrifugation (14.000 g, 30 min, at 4^°^C). Supernatant protein digests were collected and stored at -20 ^o^C prior to analysis.

#### LC-MS MS—MudPIT analyses

The tryptically-digested venom sample was loaded into the biphasic capillary columns (100 μm internal diameter) containing a strong cation exchanger resin (Luna 5μm, Phenomenex, Ca; 2.5cm) followed by reversed phase chromatography (Aqua C18 5μm, Phenomenex, Ca; 2.5cm). The first step sample was desalted by using a 12-cm column (75 μm internal diameter) packed with the same reversed phase matrix. For mass spectrometry, an automated 12-step MudPIT separation method was used [22,43]. Eluent solutions were: A (water/acetonitrile/formic acid—95:5:0.1 v/v/v); B (water/acetonitrile/formic acid—20:80:0.1, v/v/v) and C (ammonium acetate 500 mM, with 5% acetonitrile and 0.1% formic acid). The flow rate was 0.150 μL/min. The peptides eluted from the LC-MS column were directly electrosprayed into the LTQ-XL Orbitrap mass spectrometer (Thermo, San Jose, CA). HCD fragmentation was employed and MS1 data were acquired at a resolution of 60,000. The analysis was controlled by the XCalibur software (Thermo, San Jose, CA).

#### Data process and database search

The resulting fragment spectra were analyzed using MASCOT search engine (Matrix Science, UK) against the generated database of predicted proteins from the NGS transcriptome of *P*. *nigrivinter* (29,967 proteins from 49,992 unique sequences), performed in the present work, with parent and fragment tolerances of 0.1 Da. Iodoacetamide derivatives of cysteine and methionine oxidation were specified in MASCOT, as fixed and variable modifications, respectively. Only peptides with a minimum of five amino acid residues and which showed significant threshold (p < 0.05) in Mascot-based score were considered. Peptide abundance was calculated using the emPAI (Exponentially modified protein abundance index) [44], obtained directly from MASCOT.

### Supporting data

Raw sequence reads from NGS transcriptome were deposited in the NCBI Short Read Archive SRR5929664 under Bioproject accession number PRJNA397584. Transcriptome Shotgun Assembly project has been deposited at DDBJ/EMBL/GenBank under the accession GFUY00000000. The version described in this paper is the first version, GFUY01000000. Data from CS transcriptome were deposited in the EST database (NCBI): dbEST JG016062-JG017285. Mass spectrometry proteomics data were deposited in the ProteomeXchange Consortium [45]via the PRIDE partner repository [46] with the dataset identifiers PXD008909 and 10.6019/PXD008909.

## Results and discussion

In this work, we were interested in providing a broad screening of the venom proteins produced in *P*. *nigriventer* spider venom glands. To accomplish this goal, we combined conventional and next generation cDNA sequencing with MudPIT proteomic approach to perform a large-scale investigation of *P*. *nigriventer* venom.

### Transcriptomics

#### Next-generation sequencing (NGS) transcriptome

To obtain an in-depth panorama of the proteins expressed in *P*. *nigriventer* venom glands, we performed Next Generation Sequencing analyses using Illumina technology. A cDNA library was constructed from mRNAs extracted from venom glands of twenty adult *P*. *nigriventer* spiders and after high-throughput paired-end sequencing, 75,620,396 raw reads were obtained. After preprocessing steps, we obtained 36,448,250 high quality reads. All these reads were used to perform a *de novo* assembly by the software Trinity, with CuffFly parameter. It resulted in 49,992 unique sequences (assembled transcripts) and 42,917 unigenes (as defined by Trinity output). Gene expression was quantified in Fragments per Kilobase per Million mapped reads (FPKM). Overall results of Illumina sequencing and assembly output are shown in Table 1.

**Table 1:** Summary of RNA-sequencing statistics and assembly results.

**Total raw reads**	75,620,396
**High quality reads**	36,448,250
**Total unique sequences**^**a**^	49,992
**Total unigenes**^**b**^	42,917
**Contig N50**	1284 bp
**Median transcript size**	573 bp
**Average transcript size**	926 bp
**Total assembled bases**	46,283,445

^a^ Assembled transcripts (including isoforms from unigenes)

^b^ Representative transcripts putatively from the same locus

To evaluate the quality and the coverage of *P*. *nigrivinter* transcriptome assembly, we used the CEGMA pipeline, which showed that 213 (85.89%) proteins from the Core Eukaryotic Genes (CEGs) were identified in the transcriptome. Using the BUSCO core gene set, which is based on orthologous genes from OrthoDB, 932 (95.29%) proteins were identified from the 978 core gene set, indicating a high completeness of the transcriptome.

**Fig 1:**
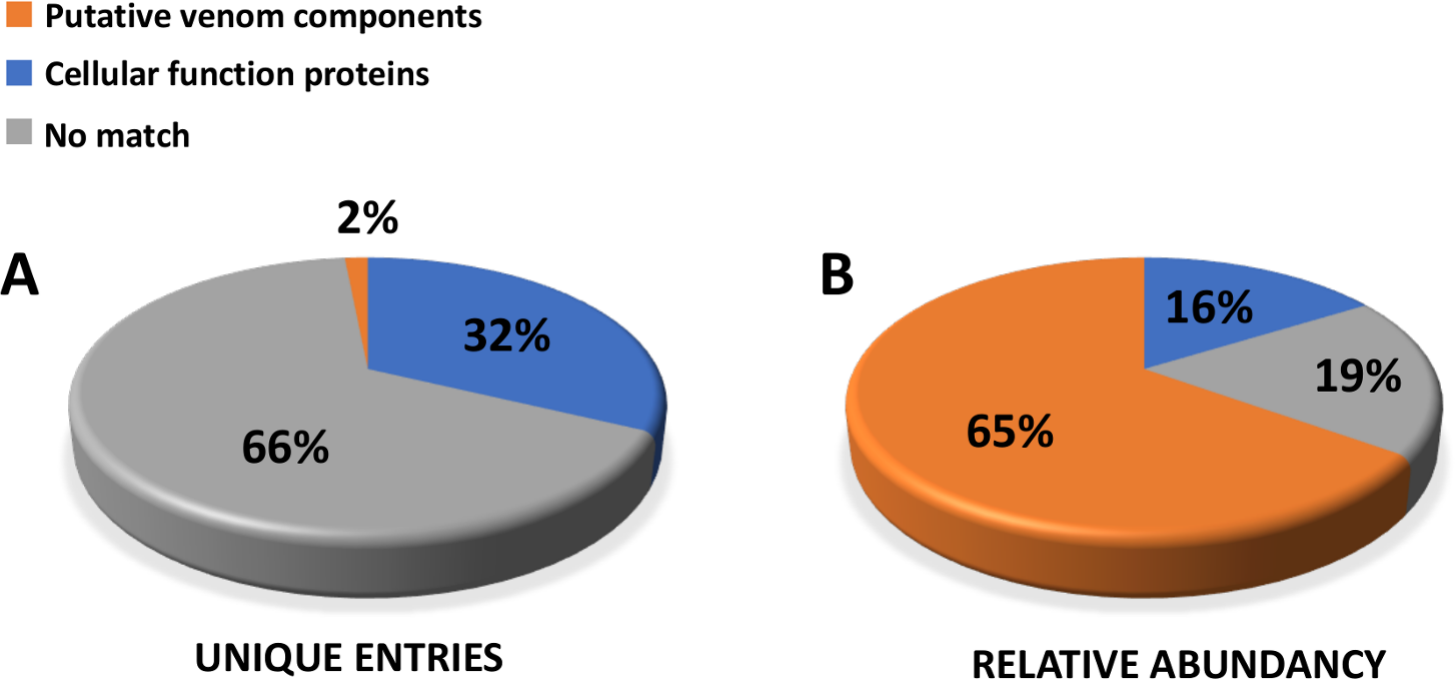
General composition of *P*. *nigriventer* venom gland transcriptome sequenced by NGS. Unique sequences were searched against UniProt database and classified as ‘putative venom components’ or ‘cellular function proteins’. Left graph shows relative proportions expressed as percentages of unique sequences. Most of the unique sequences (66%) did not match any sequence from UniProt database (e-value < 1e^-5^). Right graph shows relative proportions expressed as percentages of abundance (FPKM) of transcripts belonging to each category.

To investigate toxins and other venom proteins expressed in *P*. *nigrivinter* venom glands, the unique sequences (assembled transcripts) were searched against UniProt/Swissprot, Animal Toxin Database and TSA database, using BLASTx with cut-off e-value < 1e^-5^. For the scope of this work, only UniProt/Swissprot database was adopted as reference to analyze unique sequences. Sequences that matched to proteins deposited in UniProt were manually classified according to their function in two main categories: 1) ‘cellular function proteins’, which correspond to cellular components and metabolism proteins; and 2) ‘putative venom components’, corresponding to sequences that had match in Uniprot to proteins described for a venomous animal species and were similar to previously suggested venom components in other venom transcriptomic/proteomic analyses [47–62]. From the unique sequences with FPKM value higher than 1, 32% corresponded to ‘cellular function proteins’, 2% matched ‘putative venom components’ and 63% did not align to any protein deposited in UniProt (No Match) (Fig 1A). It is worth mentioning that, although ‘putative venom components’ corresponded to only 2% of the unique sequences, when considering the sum of their relative abundance, measured as FPKM, for each unique sequence, this category corresponded to 65% of the gene expression in the venom glands (Fig 1B). This abundance of ‘putative venom components’ over ‘cellular function proteins’ confirms that most of the proteins produced in the venom glands are related to venom functions. UniProt annotation of the one hundred most abundant unique sequences according to FPKM values is listed on S1 Table, confirming the predominance of venom components in *P*. *nigriventer* venom gland transcriptome.

**Fig 2:**
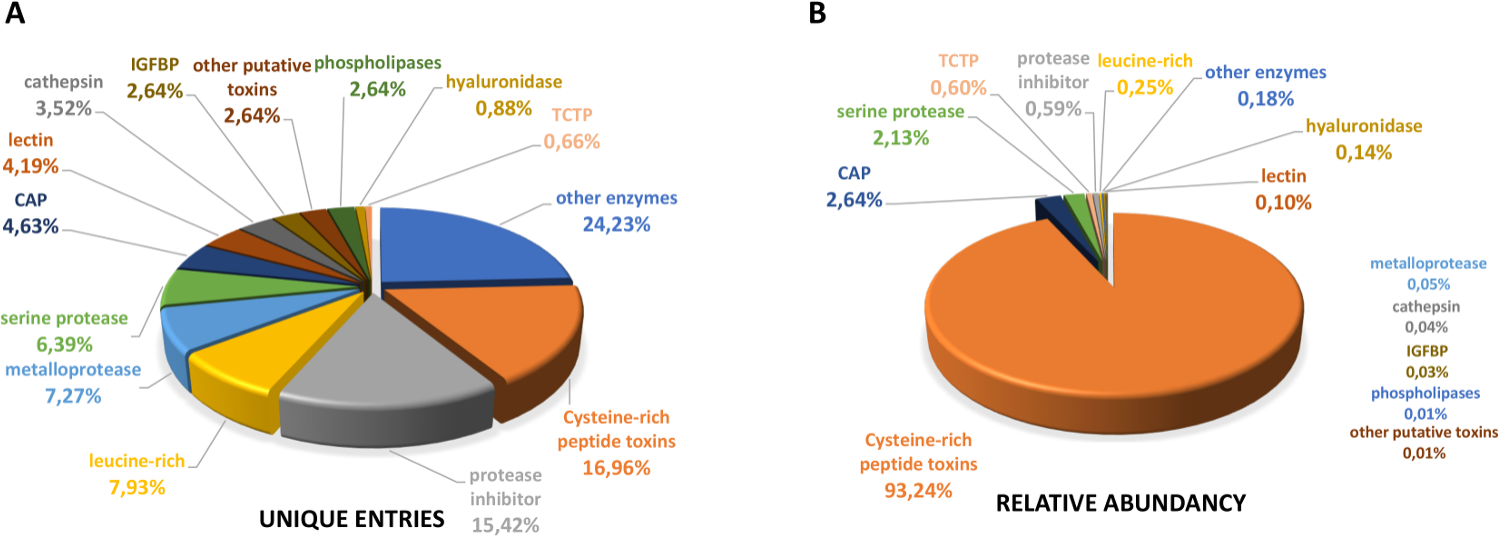
Diversity and abundance of putative venom components from *P*. *nigriventer* venom gland transcriptome sequenced by NGS. Unique sequences were searched against UniProt database and classified into known toxin subfamilies. Left graph shows relative proportions expressed as percentages of unique entries. Right graph shows relative proportions expressed as percentages of abundance (FPKM) of transcripts belonging to each subfamily.

According to putative venom functions, the sequences were separated into distinct categories (Fig 2A). Initially, 28 categories were found, based on molecular families previously described as venom components. Some of the least abundant families were further grouped into 14 broader categories (ex: other enzymes, protease inhibitors) as shown in Fig 2. Unique sequences corresponding to cysteine-rich peptide toxins account for 17% of the putative venom proteins. However, when we consider the sum of the relative abundance (FPKM) of each unique sequence, this class represents almost 94% of the venom components (Fig 2B).

**Fig 3:**
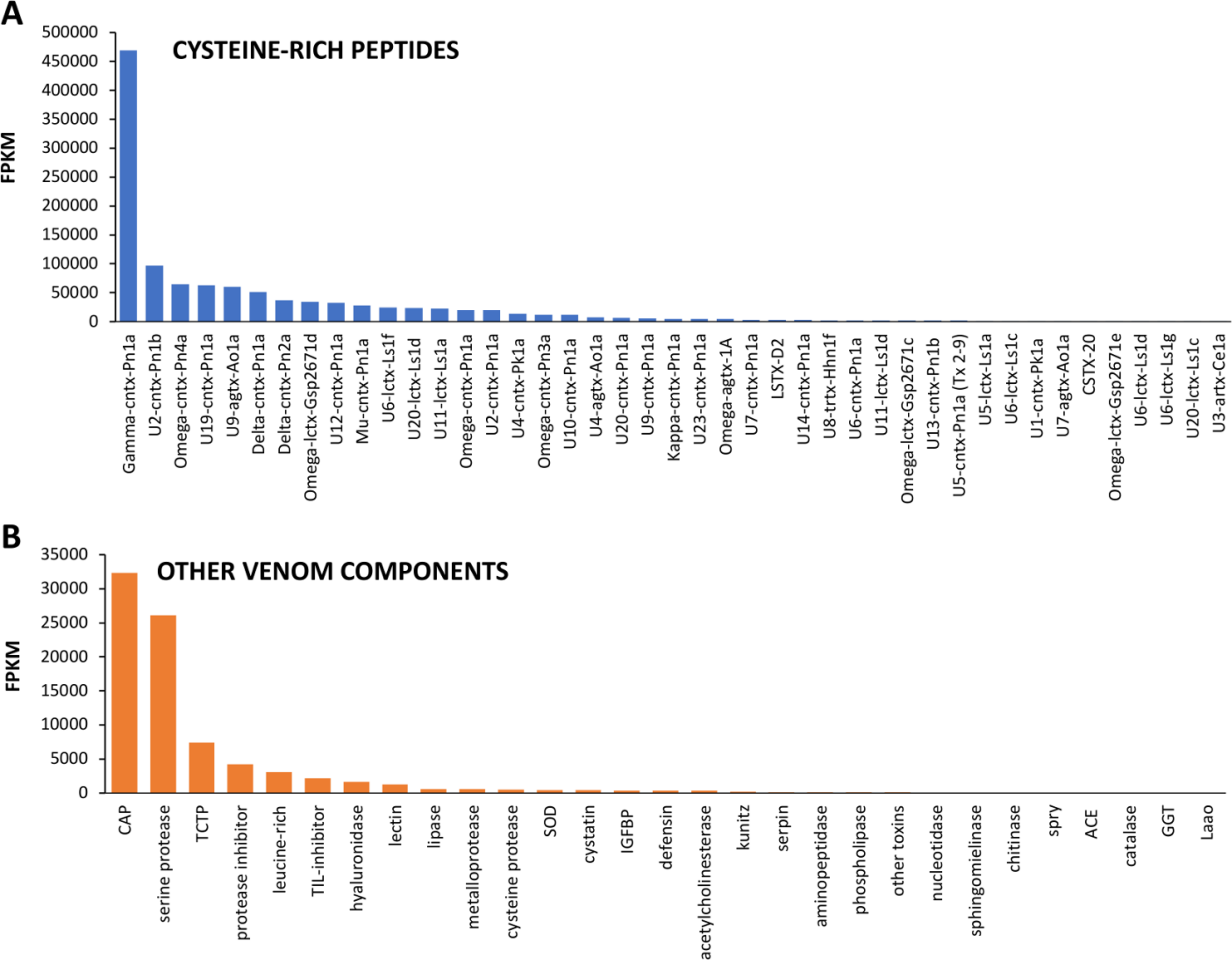
Relative abundance, expressed as FPKM, of subfamilies of the putative venom components found in the NGS analysis of *P*. *nigriventer* venom glands. A) Cysteine-rich peptide toxins and B) Other families of venom components. Unique sequences were classified into known toxin subfamilies according to UniProt database. Bars represent the sum of FPKM for each transcript belonging to the described groups.

Fig 3A and 3B show the relative abundance in FPKM of the cysteine-rich peptide toxin sequences and unique sequences of venom components, respectively, classified according to UniProt annotation.

Moreover, functional characteristics of the whole assembled transcriptome were analyzed using Gene Ontology (GO) annotations by the GOSlim, a subset of high-level view of each of the three GO ontologies. From the 49,992 assembled unique sequences, 21,882 were classified within the three namespaces of GO, namely ‘biological process’ (BP), ‘cellular component’ (CC) and ‘molecular function’ (MF), comprising 56 distinct categories for BP, 30 for CC and 39 for MF (S1 Fig). Biological processes, such as metabolic and translation processes are abundant, indicating that the venom gland is highly metabolically active and committed to intensive protein synthesis. Moreover, transcripts with molecular functions related to protein synthesis and processing, as DNA and RNA binding, transcription factor activity and oxidoreductase activity, required for toxin folding, are also overrepresented, confirming that most of the venom gland activity is dedicated to venom production.

#### Conventional cDNA sequencing (CS) transcriptome

**Fig 4:**
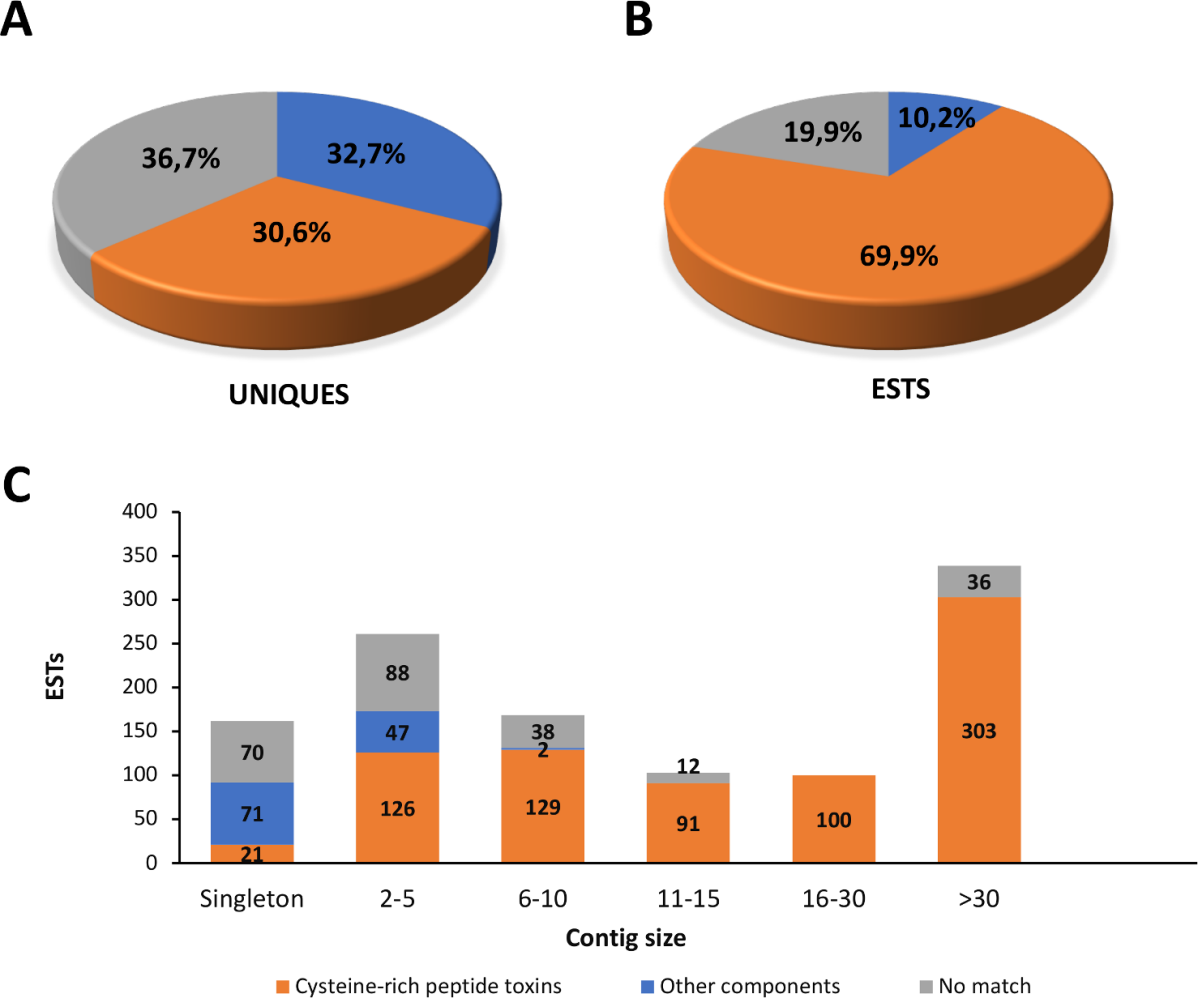
Conventional sequencing annotation. Annotation distribution of unique sequences (A) and ESTs (B) against UniProt. C) Distribution of EST annotation per Contig size (number of ESTs used to generate contig sequences). Numbers inside the bars are the raw numbers of ESTs per annotation class.

In addition to the NGS analysis of *P*. *nigriventer* venom glands transcriptome, we performed conventional sequencing (CS) transcriptome, primarily aiming at discovering novel cysteine-rich peptide toxins, using an optimized protocol. Furthermore, data from CS cDNA library were also used as a quality control for *de novo* sequence assembly in the NGS transcriptome. A standard and unidirectional library was generated using only cDNA fragments from 300 to 800 bp, which overlaps the size of virtually every known spider toxins belonging to the group of cysteine-rich peptide toxins [63]. Random sequencing of the cDNA library resulted in 1,476 electropherograms and, after editing, 1,224 good quality reads were obtained (mean length 383 bp). The reads were clustered and assembled into 294 unique sequences (mean length 428 bp) including 132 contigs (represented by more than 1 EST) and 162 singletons (1 EST). Although most of the unique sequences corresponded to singletons, they represent only 13.2% of the total ESTs, indicating that most of the unique sequences were assembled as contigs. Unique sequences were aligned against UniProt datababase (E-value cutoff < 1e^-5^), and the best hit was selected to annotate the sequences. According to UniProt annotation, they were divided into two categories: 1) Cysteine-rich peptide toxins: for sequences matching inhibitory cysteine knot toxins (32.7% of unique sequences and 69.3% of ESTs); 2) Other components, for sequences that matched proteins that have not been previously classified as cysteine-rich peptide toxins (30.6% of unique sequences and 10.2% of ESTs). Sequences with no match at UniProt corresponded to 36.7% of unique sequences and 20.9% of ESTs (Fig 4A and 4B). Clusters with more than one EST (Contigs) are more represented by cysteine-rich peptide toxin sequences than by ‘Other components’ or ‘No match’ sequences (Fig 4C). This evidence of the high redundancy of cysteine-rich peptide toxins is in accordance with the strategy employed in the cDNA library construction, but may also reflect the gland specialized function in producing these molecules.

### Proteomics

**Fig 5:**
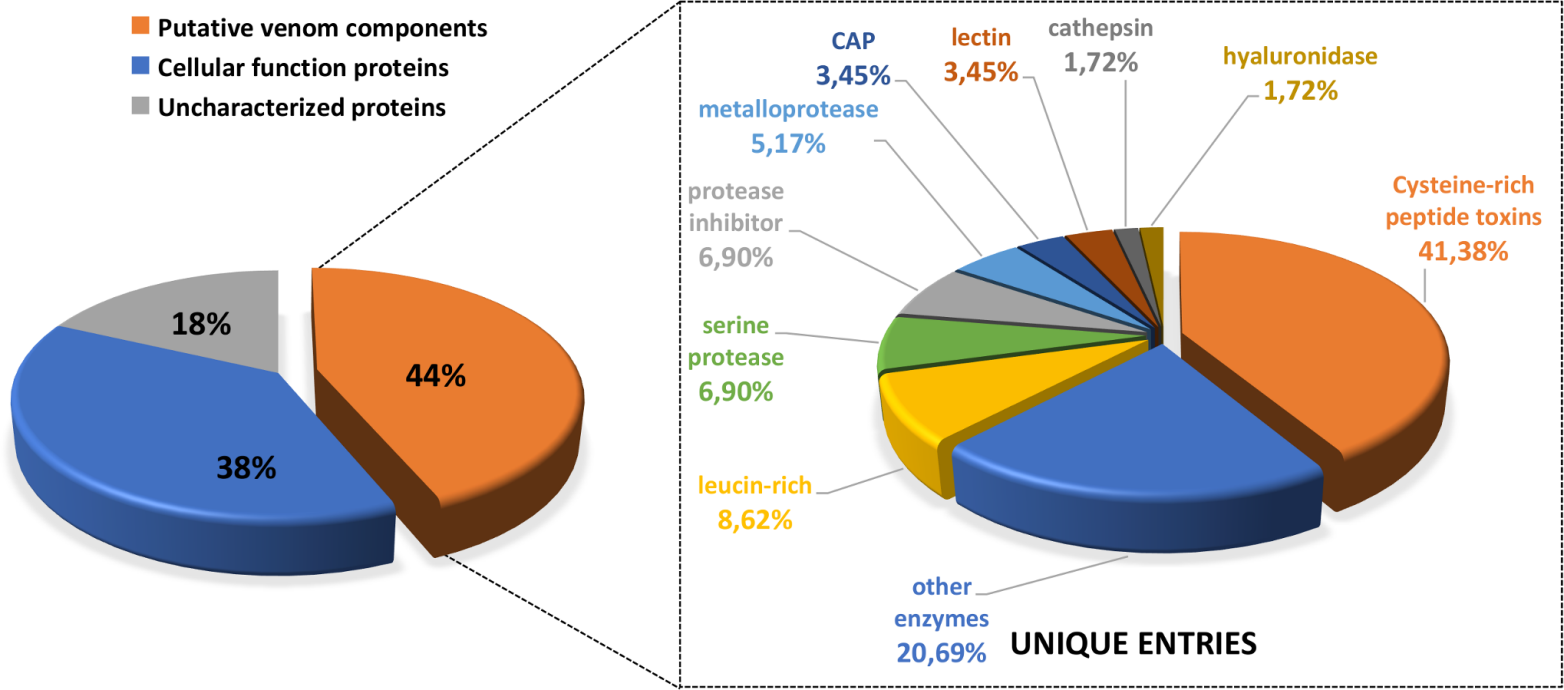
*P*. *nigriventer* venom composition analyzed by MudPIT proteomic technique. Left graph shows the proportion of components detected by venom analysis. The peptide sequences found were searched against the NGS transcriptomic database and classified according to their UniProt annotation as ‘putative venom components’ or ‘cellular functions’. Eighteen percent of the retrieved proteins did not match any sequence from the database. Right graph shows putative venom components divided into subfamilies of putative toxins. The proportion of each category was calculated by the sum of the emPAI.

*P*. *nigriventer* venom was also investigated by mass spectrometry analysis, using a MudPIT approach, to validate the proteins deduced from the transcriptome assembly and to acknowledge proteins that are indeed secreted in the venom. The resulting sequences deduced from the fragment spectra of *P*. *nigriventer* venom were searched using MASCOT search engine, against a database of predicted proteins from the NGS transcriptome. All proteins identified in the proteomic analysis had matches with the transcriptome dataset, which in turn validated the transcriptome assembly. Venom proteome analysis resulted in 586 peptide sequences that matched 194 unique sequences identified in the NGS transcriptome (corresponding to 176 different proteins deposited in Uniprot) (S2 Table). According to UniProt transcriptome annotation, proteins identified in the proteomic analysis were classified in three major groups: 1) ‘putative venom components’, comprising 85 different NGS-identified protein sequences (44%) matched by 313 peptides; 2) ‘cellular functions’ (metabolism and cellular components), comprising 74 different NGS-identified protein sequences (38%) with 174 matching peptides; and 3) no-match sequences, comprising 35 different NGS-generated protein sequences (18%) matched by 99 peptides (Fig 5). The proteins related to putative venom components were further classified in subcategories according to the class of the venom component, revealing that most of them, 24 sequences (41%), correspond to cysteine-rich peptide toxins (Fig 5). The proportion of each category was calculated by the sum of the emPAI (Exponentially modified protein abundance index) [44,64] of all the peptides that matched each category.

### *P*. *nigriventer* venom cocktail revealed by transcriptomic and proteomic approaches

#### Cysteine-rich peptide toxins as major venom components

**Fig 6:**
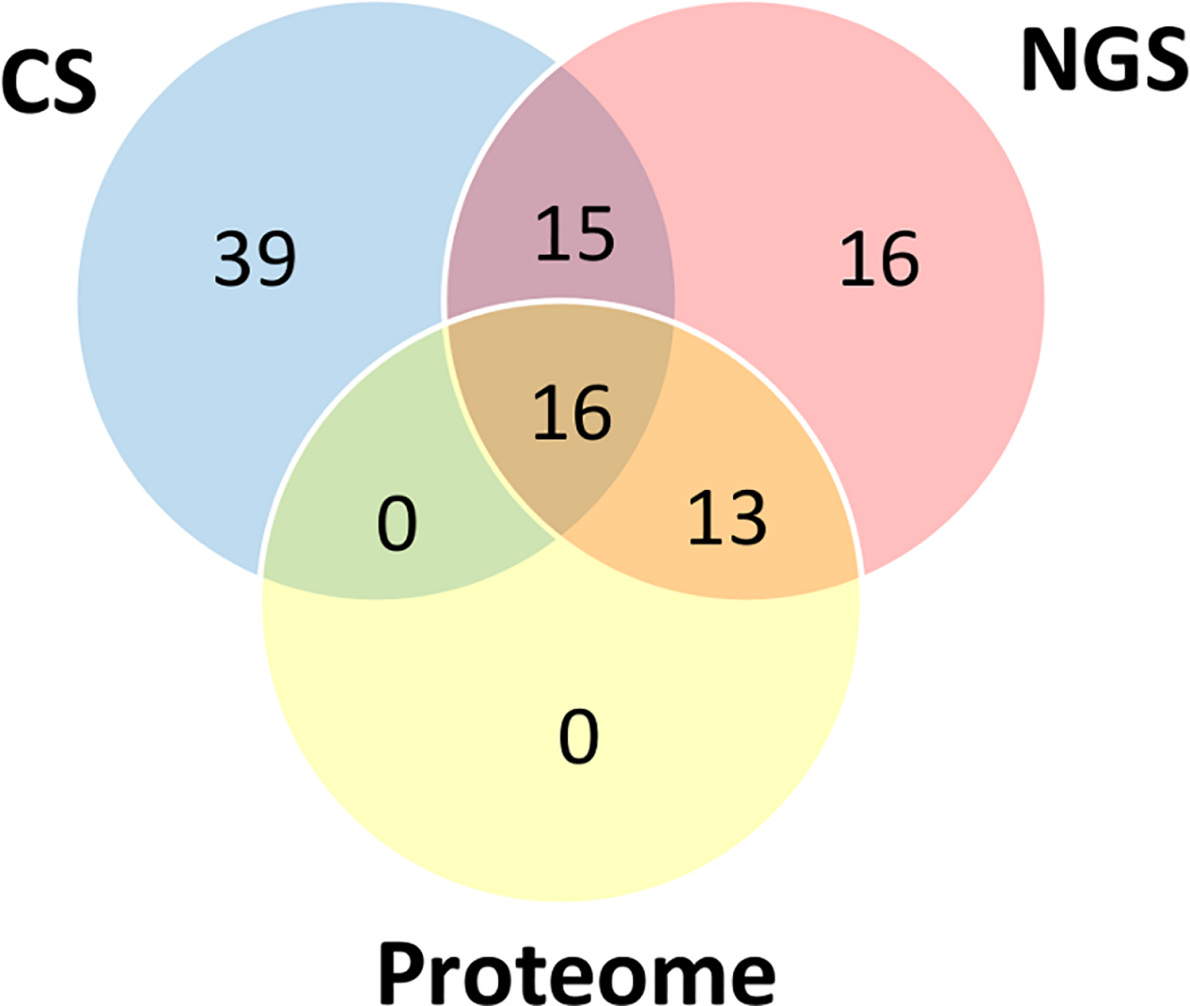
Venn diagram representing the total number of unique cysteine-rich peptide toxins found in *P*. *nigriventer* venom by each technique used.

Cysteine-rich peptide toxins are the most abundant component of *P*. *nigriventer* venom, accounting for 17% of the putative venom components in the NGS transcriptome (Fig 2A). Furthermore, when we consider the sum of the relative expression of each unique sequence (FPKM), this class represents 94% of the putative venom components (Fig 2B). This result was corroborated both by conventional cDNA sequencing and proteome results (Figs 5 and 6), confirming that most of the proteins expressed in *P*. *nigriventer* venom glands correspond to cysteine-rich peptide toxins.

By using three different approaches (NGS, CS and MudPIT), we identified 98 different cysteine-rich peptide toxin sequences expressed in *P*. *nigriventer* venom glands (S3 Table). Nineteen sequences have mature sequences that are identical to previously described *Phoneutria* toxins and 30 sequences show high similarity (> 90%). However, many of the toxin sequences identified have a low degree of similarity to previously described toxins and can be considered as novel sequences of putative toxins. For identification purposes, in this work, cysteine-rich peptide toxin sequences were identified by the initials PN in numerical order (e.g., PN045). Toxins to which they presented similarity were designated according to the nomenclature suggested by King and colleagues [65].

Thirty-nine cysteine-rich peptide toxin sequences were only identified by CS; 16 sequences only by NGS; and 15 were identified by both methodologies, without further proteomic identification. However, 13 sequences identified by NGS and 16 sequences identified by both transcriptomic methodologies had matches in the venom proteome, summing 29 confirmed cysteine-rich peptide toxins (Fig 6). The coverage in the sequences of the given peptides by proteomic data are highlighted in red in S3 Table.

According to UniProt/SwissProt database, cysteine-rich peptide toxins were classified in 14 different structural families and 14 sequences had no family classification (S3 Table). In addition, cysteine frameworks from these sequences were checked for similarity to toxins with ICK structural motif sequences and also through the Knottin database (http://knottin.cbs.cnrs.fr/) [66] to predict putative ICK structures. From the 98 cysteine-rich peptide toxins, 84 were predicted as ICK toxins. Classical ICK toxins are characterized by three disulfide bridges following connective C1-C4, C2-C5, C3-C6, where the two first disulfides form a loop, crossed by the third disulfide bond, forming a knot. It conforms as an anti-parallel, triple-stranded β-sheet stabilized by the cysteine knot, conferring an unusually high stability [67]. Many of those ICK toxins are active on ion channels and chemical receptors from vertebrates and invertebrates, being responsible for the neurotoxic symptoms of *P*. *nigriventer* envenoming. This class of toxins is well represented in most spider venoms [47,51,52,58,68–70], which demonstrates their great importance for spider survival.

Many sequences of the identified cysteine-rich peptide toxins, including ICKs, differ by a single or few amino acid substitutions, stressing the combinatorial fashion that the genes encoding these toxins were generated [58,68,71,72]. Although most toxin isoforms were detected only by CS, the chromatograms and contigs alignments were carefully checked, confirming that the existence of these variations is not due to assembly artifacts. Indeed, some *Phoneutria* toxins previously deposited in UniProt (δ-ctenitoxin-Pn2c (UniProt: O76199), U2-ctenitoxin-Pn1a (UniProt: P29423), U11-ctenitoxin-Pn1a (UniProt: P0C2S7), ω-ctenitoxin-Pn3a (UniProt: P81790) were only detected by CS (S3 Table). Therefore, the CS approach may be more accurate for detection of minor mutations due to the difficulty of *de novo* assembly algorithms in NGS in distinguishing real mutations from sequencing errors in the absence of a reference genome [60]. As a result, some sequences in NGS transcriptome are obtained only for the most expressed transcripts, which can underestimate the total number of coded toxins [53]. Besides that, in the proteomic approach we identified only 29 from the 98 sequences detected, which indicates that this technique may not be suitable for detecting lowly expressed isoforms as well. Therefore, the combination of Conventional and Next-generation sequencing was an efficient strategy for the discovery of lowly expressed isoforms in the transcriptome of *P*. *nigriventer* venom glands.

The cysteine-rich peptide toxins identified in this work were classified in nine groups according to their cysteine frameworks (Table 2).

**Table 2:** Classification of the cysteine-rich peptide toxins identified, according to their cysteine frameworks.

Group	Cysteine framework (predicted folding)	Predicted molecular function	Spider toxin family (# sequences)	Species toxin similarity
**I**	C-C-CC-C-C (ICK)	Ca^+2^ channel modulator/ Protease inhibitor/ Unknown	Spider neurotoxin 21C2 (9) Huwentoxin-1: PNTx27C4 subfamily (5); Tx2-9 subfamily (1) Proteinase inhibitor (1) Not in a family (1)	*Phoneutria / Ctenus*
**II**	C-C-CC-CXC-CXC (ICK)	Ca^+2^ channel modulator/ K^+^ channel modulator/ Unknown	Plectoxin superfamily: Tx3 family (16) Omega-lycotoxin (3) CSTX superfamily (2) U6-lycotoxin (4) U11-lycotoxin (2) Not in a family (3)	*Phoneutria / Lycosa / Cupiennius*
**III**	C-C-CC-C-CC-C-C-C (Unknown)	Protease inhibitor / antimicrobial activity / Unknown	Spider wap 1 (1) Spider wap 2 (4)	*Lycosa*
**IV**	C-C-CC-C-C-CXC-C-C (Unknown)	Unknown	Not in a Family (9)	*Phoneutria / Caerostris / Viridasius*
**V**	C-C-CXCC-CXC-CXC-C (ICK)	Na^+^ channel modulator / NMDA receptor modulator	Spider toxin Tx2 (18)	*Phoneutria*
**VI**	C-C-CXCC-CXC-CXC-C-C-C (ICK)	Ca^+2^ channel modulator/ Unknown	Type II/III omega-agatoxin (1) Not in a Family (1)	*Phoneutria/ Agelenopsis*
**VII**	C-C-CXCCXC-CXC-CXC-C-C (ICK)	Ca^+2^ channel modulator/ Unknown	Spider toxin Tx3-6 (4) Type II/III omega-agatoxin (1)	*Phoneutria*
**VIII**	C-C-CXCC-CXC-CXC-C-C-C-C-C (ICK)	Ca^+2^ channel modulator/ Na^+^ channel modulator	Omega-agatoxin superfamily: Type II/III omega-agatoxin family (9); Tx1 family (2)	*Phoneutria*
**IX**	C-CXC-CXC-CXC-C-C-C Unknown	Ca^+2^ channel modulator	Type I omega-agatoxin	*Agelenopsis*

#### Group I (C-C-CC-C-C)

**Fig 7:**
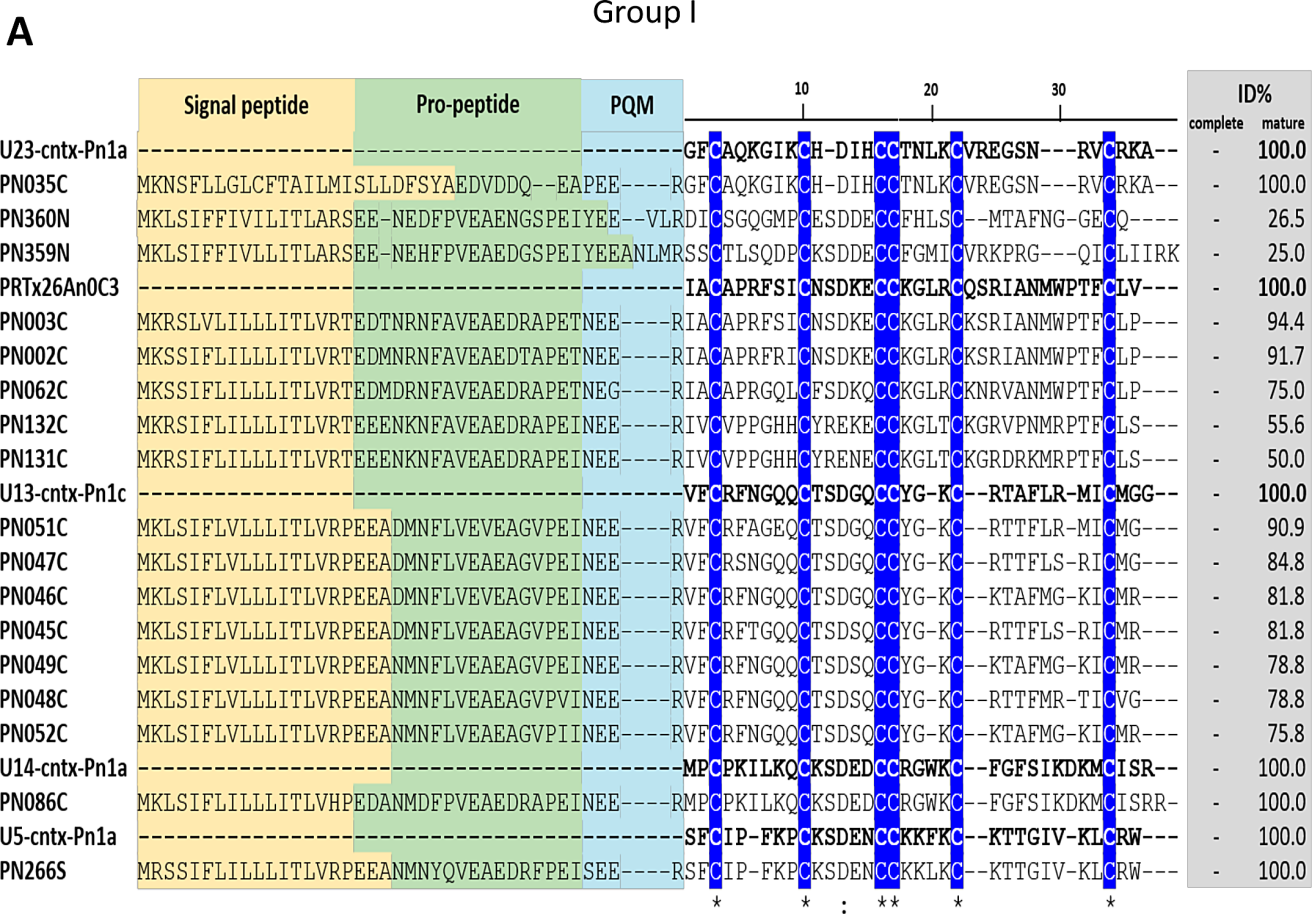
Sequence alignments of cysteine-rich peptide toxin precursors from group I. Alignment was performed with MUSCLE, Signal peptide is highlighted in yellow, propeptide is highlighted in green and processing quadruplet motif (PQM) is highlighted in cyan. Conserved cysteines are marked in blue. Percentage of identity (ID%) with the reference protein was calculated using the tool EMBOSS Stretcher for pairwise sequence alignment using either the complete or processed mature sequence. U23-cntx-Pn1a (UniProt: P84015), PRTx26An0C3 (UniProt: P86418), U13-cntx-Pn1a (UniProt: P83894), U14-cntx-Pn1a (UniProt: P83998) and U5-cntx-Pn1a (UniProt: P29426), from *P*. *nigriventer*, were used as references.

A total of 17 putative cysteine-rich peptide toxin sequences were described for this group of sequences with 6 cysteine residues (S3 Table, Fig 7). Most of the sequences in this group have similarity to toxins from *Phoneutria* species and, according to their cysteine framework, they probably adopt the classical ICK conformation. Despite sharing the same putative structural conformation, they belong to five different structural toxin families (Table 2) and present different pharmacological activities. Toxins PRTx26An0C3 (UniProt: P86418) and U4-ctenitoxin-Pr1a (UniProt: P83892) can cause spastic paralysis and death in mice and are moderate inhibitors of L-Cav1/CACNA1 type calcium channels. On the other hand, toxin U6-ctenitoxin-Pk1a (UniProt: P83910), despite causing spastic paralysis and death in mice, has no detectable action on those channels [73]. In contrast, toxins U23-ctenitoxin-Pn1a (UniProt: P84015) and U13-ctenitoxin (UniProt: P83894) have no toxic effect on mice. The isoforms U13-ctenitoxin-Pn1b (UniProt: P84017) and U13-ctenitoxin-Pn1c (UniProt: P84018) are lethal to flies [18]. U14-ctenitoxin-Pn1a (UniProt: P83998) has no toxic effect on mice or on insects, but it has a striking similarity in its N-term amino acid sequence with various serine protease inhibitors from cucurbitaceaes, suggesting that it may also perform this function. U5-ctenitoxin-Pn1a (UniProt: P29426) is toxic to mice and flies, and causes similar effects on mice as observed with δ-ctenitoxin-Pn2a (UniProt: P29425), which inhibits the inactivation of voltage-gated sodium channels [74,75], although being much less toxic. Furthermore, two putative mature toxins (PN359 and PN360) showed sequence identity (44 and 68% respectively) to toxin U21-ctenitoxin-Co1a (UniProt: P85032) from *Ctenus ornatus* venom, which is not toxic to mice. The putative polypeptides encoded by sequences PN035 and PN086 are identical to toxins U23-ctenitoxin-Pn1a and U14-ctenitoxin-Pn1a, respectively. These two toxins, despite having been verified in another proteomic approach (18), were not detected in our proteomic analysis.

#### Group II (C-C-CC-CXC-CXC)

**Fig 8:**
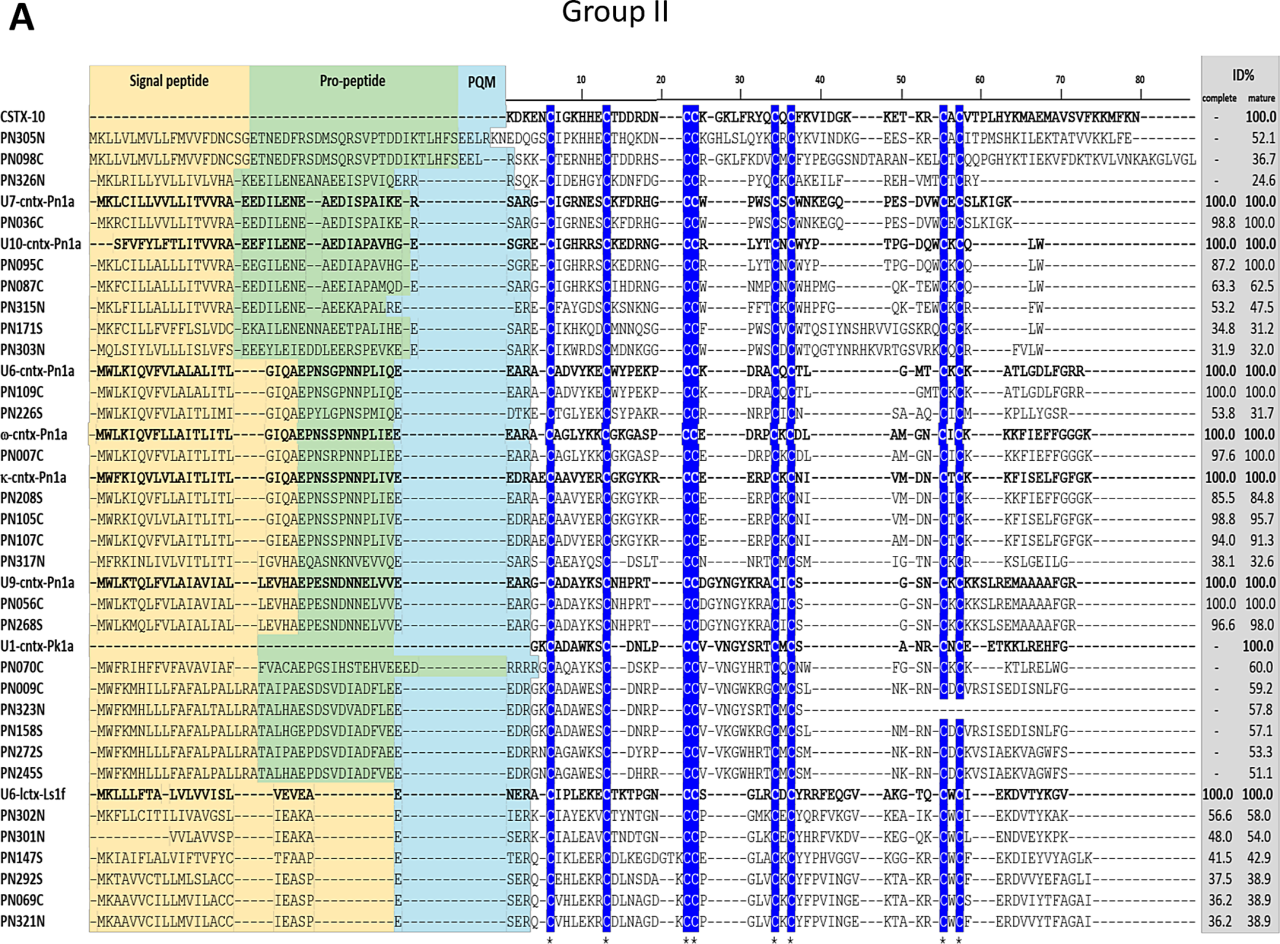
Sequence alignments of cysteine-rich peptide toxin precursors from group II. Alignment was performed with MUSCLE, Signal peptide is highlighted in yellow, propeptide is highlighted in green and processing quadruplet motif (PQM) is highlighted in cyan. Conserved cysteines are marked in blue. Percentage of identity (ID%) with the reference protein was calculated using the tool EMBOSS Stretcher for pairwise sequence alignment using either the complete or processed mature sequence. CSTX-10 (UniProt: B3EWT0), from *C*. *salei* spider; U7-cntx-Pn1a (UniProt: P81791), U10-cntx-Pn1a (UniProt: P0C2S9), U6-cntx-Pn1a (UniProt: P81793), ω-cntx-Pn1a (UniProt: O76201), κ-cntx-Pn1a (UniProt: O76200), U9-cntx-Pn1a (UniProt: P0C2S6), from *P*. *nigriventer* spider; and U1-cntx-Pk1a (UniProt: P83895), from *P*. *keyserlingi* spider were used as references.

Thirty sequences were classified in this group of 8 cysteine-residue sequences (S3 Table, Fig 8). They belong to five different structural families and are similar to toxins from venoms of *Phoneutria* species and also have sequence similarity with toxin sequences from *Cupiennius* and *Lycosa* species (Table 2). κ-ctenitoxin-Pn1a (UniProt: O76200) inhibits potassium channels [76] and ω-ctenitoxin-Pn1a (UniProt: O76201) and U9-ctenitoxin-Pn1a (UniProt: P0C2S6) are active on calcium channels [77–79]. ω-lycotoxin-Gsp2671c (UniProt: A9XDG1) from *Lycosa kazakhstanicus* spider is also a calcium channel inhibitor [80]. U7-ctenitoxin-Pn1a (UniProt: P81791) and U9-ctenitoxin-Pn1a showed antinociceptive activity on mice [81,82]. Sequences PN069 and PN321 have 45% identity to U11-lycotoxin-Ls1d (UniProt: B6DD10) and U6-lycotoxin-Ls1f (UniProt: B6DCV6) from *Lycosa singoriensis*. Although these lycotoxins have experimental evidences only at transcript level [52], sequences PN069 and PN321 were confirmed in our proteome analysis, which is an evidence that these are toxin-coding sequences. Toxins CSTX-12 (UniProt: B3EWS6) and CSTX-10 (UniProt: B3EWT0) from *C*. *salei* have experimental evidence at protein level [54]. CSTX-12 is cleaved into two chains, which are connected via ICK fold. Although sequence PN098 has 66% identity to this entire primary sequence, there is no evidence that this toxin precursor is also processed into two separate mature chains. Sequence PN305N has 53% identity to toxin CSTX-10, which has only one ICK chain motif. This sequence was confirmed by CS and validated by proteomic analysis. It is important to emphasize that some of the sequences found in this group show low similarity to the above described proteins and may have distinct functions.

#### Groups III (C-C-CC-C-CC-C-C-C) and IV (C-C-CC-C-C-CXC-C-C)

**Fig 9:**
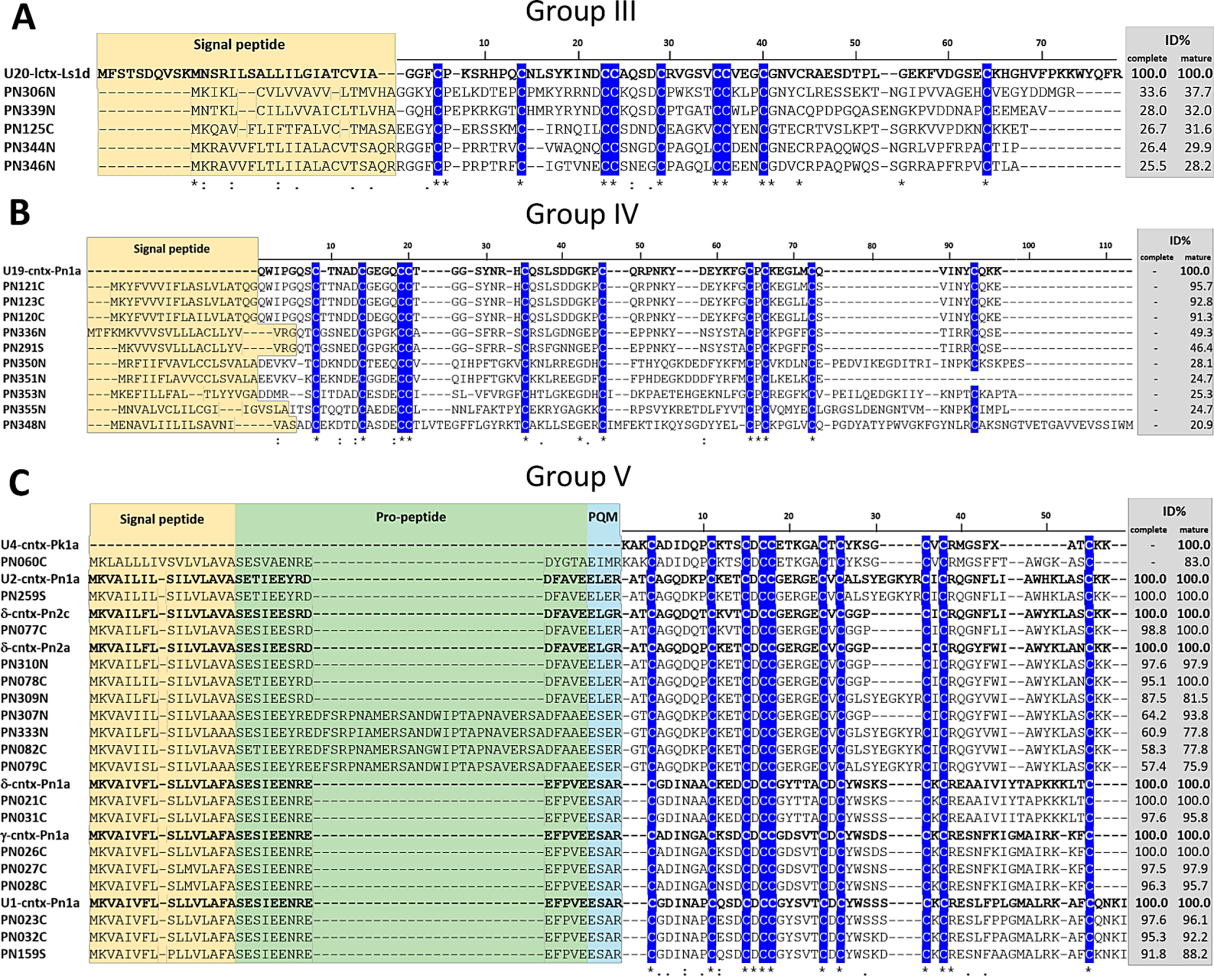
Sequence alignments of cysteine-rich peptide toxin precursors from groups III-V. Alignment was performed with MUSCLE, Signal peptide is highlighted in yellow, propeptide is highlighted in green and processing quadruplet motif (PQM) is highlighted in cyan. Conserved cysteines are marked in blue. Percentage of identity (ID%) with the reference protein was calculated using the tool EMBOSS Stretcher for pairwise sequence alignment using either the complete or processed mature sequence. A) Group III alignment, using U20-lctx-Ls1d (UniProt: B6DCY1), from *L*. *singoriensis* spider, as reference. B) Group IV alignment, using U19-cntx-Pn1a (UniProt: P83997), from *P*. *nigriventer* spider, as reference. C) Group V alignment, using U2-cntx-Pn1a (UniProt: P29423), δ-cntx-Pn2c (UniProt: O76199), δ-cntx-Pn2a (UniProt: P29425), δ-cntx-Pn1a (UniProt: P59368), γ-cntx-Pn1a (UniProt: P59367), U1-cntx-Pn1a (UniProt: P61229), from *P*. *nigriventer* spider, and U4-cntx-Pk1a (UniProt: P83896), from *P*. *keyserlingi* spider, as references.

Sequences from groups III and IV present a framework of 10 cysteines and differ from the other groups for not having propeptide sequences (S3 Table, Fig 9A and 9B). Normally, spider toxins are reported as having a conserved gene structure as well as precursor organization; being composed of three segments that include signal peptide, propeptide, and mature peptide [83]. Although it is not common, toxin precursors lacking propeptide have been reported in some spider transcriptomes [49,52,84,85]. In addition, according to their cysteine framework, sequences from groups III and IV are not predicted to adopt the ICK structural motif, presenting an unknown folding (Table 2).

Group III comprises 5 sequences presenting 40–45% identity to putative lycotoxins from spider *Lycosa singoriensis*, which also do not have propeptide and are classified in Spider wap 1 and 2 toxin families (Table 2). According to UniProt/Interproscan, these lycotoxins have structural domains of peptidase inhibitors and putative antimicrobial activity, but there is no experimental confirmation of their molecular targets or function. It is noteworthy that the unique sequences that are similar to lycotoxins are the second most abundant cysteine-rich peptide toxins expressed in the NGS transcriptome (Fig 3A). This indicates that, although *P*. *nigriventer* venom has been widely studied, there are some classes of toxins that, despite being abundantly expressed, are still unknown.

Group IV has 9 sequences and none of them have family classification yet. Three of them presented punctual differences from the non-toxic peptide U19-ctenitoxin-Pn1a (UniProt: P83997) from *P*. *nigriventer* [18]. Two very similar sequences (PN291, PN336) have 55 and 58% identity to non-toxic U9-ctenitoxin-Pr1a (UniProt: P83893), from *Phoneutria reidyi* spider, found in the Brazilian Amazonian region. Three sequences were similar to the sequence of the putative U3-aranetoxin-Ce1a (UniProt: Q8MTX1) (36–41% identity) from *Caerostris extrusa* spider, which has the MIT-like atracotoxin domain with no attributed function. Sequence PN353 is 80% similar to the putative U2-ctenitoxin-Vf2 (UniProt: A0A1V0FW55) from *Viridasius fasciatus* spider, which presents a prokinectin domain. This structural domain includes proteins related to the circadian clock in mammals and also to the Hainantoxins (HNTX), neurotoxins from *Haplopelma hainanum* Chinese bird spider, which specifically inhibit tetrodotoxin-sensitive voltage-gated sodium channels [86].

It is important to mention that some of these cysteine-rich peptide toxin sequences classified in families III and IV, which lack propetide and were not predicted to adopt ICK conformation, were confirmed by proteomic analysis—PN123, PN339, PN350 (S3 Table). Thus, they can be considered totally novel putative toxins found in *P*. *nigriventer* venom.

#### Group V (C-C-CXCC-CXC-CXC-C)

This group has a cysteine framework with 10 cysteine residues, represented by 18 sequences, all belonging to Tx2 toxin family (Table 2). It comprises identical sequences or isoforms of toxins from *P*. *nigriventer* venom fractions PhTx2 and PhTx4 [15,87] (S3 Table, Fig 9C). Four sequences corresponded to δ-ctenitoxin-Pn2a (UniProt: P29425) and Pn2c (UniProt: O76199), which are the two most toxic peptides from *P*. *nigriventer* venom. These toxins inhibit voltage-gated sodium channel inactivation, prolonging action potentials [75,88,89]. Five sequences were similar to U2-ctenitoxin-Pn1b (UniProt: O76198), which was the third most expressed toxin in the NGS transcriptome (Fig 3A) and, considering sequence similarity, it probably has effect on sodium channels. Two sequences have identity to toxins from *P*. *keyserlingi;* PN159 has 96% identity to U2-ctenitoxin-Pk1a (UniProt: P83905), with unknown function, and PN060 is identical to U4-ctenitoxin-Pk1a (UniProt: P83896), which is very toxic to mice and house-flies [18], with possible action on ion channels. Group V also comprises sequences that are identical or similar to δ-ctenitoxin-Pn1a (UniProt: P59368) and γ-ctenitoxin-Pn1a (UniProt:P59367), which have high insecticidal activity and no macroscopic behavioral effects when intracerebrally injected in mice [15,16]. Another similar toxin sequence identified in this group, U1-ctenitoxin-Pn1a (UniProt: P61229), was obtained using a δ-ctenitoxin-Pn1a probe from a *P*. *nigriventer* cDNA library [90]. δ-ctenitoxin-Pn1a showed antinociceptive activity [12].

The γ-ctenitoxin-Pn1a, along with its isoforms, is by far the most abundantly expressed toxin in the NGS transcriptome (Fig 3A). This relative abundance was confirmed by CS (high number of ESTs: 149) and by proteomics, where it also showed a high relative abundance, measured as emPAI (S2 Table). This toxin has high insecticidal activity (LD_50_ = 50ng/g in houseflies), but is also able to inhibit the NMDA subtype of ionotropic glutamate receptors of cultured rat hippocampal neurons [16]. When expressed in *E*. *coli*, the recombinant toxin presented a remarkable effect on insect sodium channel, completely inhibiting channel inactivation, but had a minor effect on mammalian sodium channels isoforms, slightly reducing the current peaks [91]. This important effect on insect channel could explain its high expression level, since insects constitute the main prey of *P*. *nigriventer*.

#### Groups VI (C-C-CXCC-CXC-CXC-C-C-C) and VII (C-C-CXCCXC-CXC-CXC-C-C)

**Fig 10:**
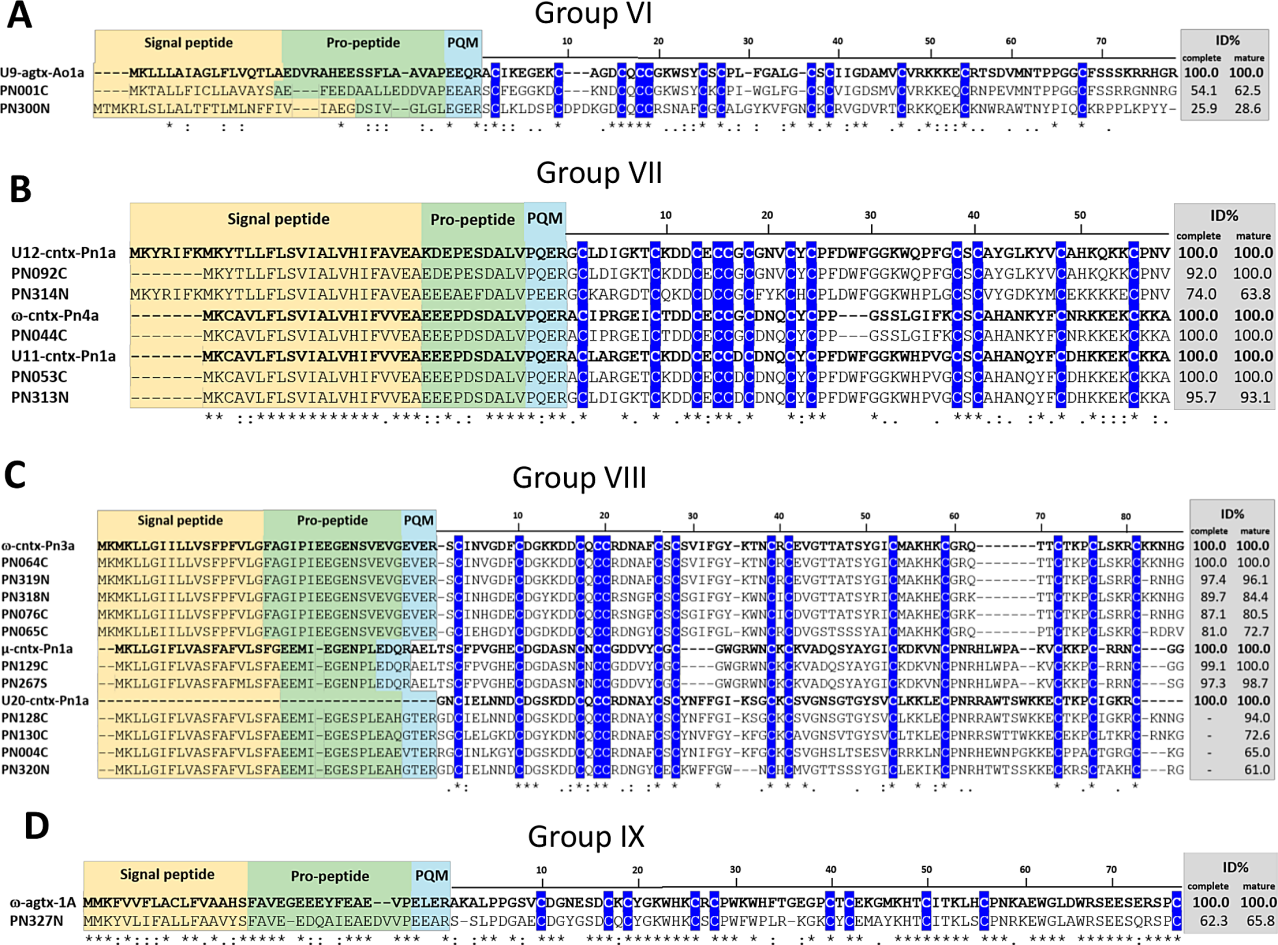
Sequence alignments of cysteine-rich peptide toxin precursors from groups VI-IX. Alignment was performed with MUSCLE, Signal peptide is highlighted in yellow, propeptide is highlighted in green and processing quadruplet motif (PQM) is highlighted in cyan. Conserved cysteines are marked in blue. Percentage of identity (ID%) with the reference protein was calculated using the tool EMBOSS Stretcher for pairwise sequence alignment using either the complete or processed mature sequence. A) Group VI alignment, using U9-agtx-Ao1a (UniProt: Q5Y4U3), from *A*. *orientalis* spider, as reference. B) Group VII alignment, using U12-cntx-Pn1a (UniProt: P0C2S8), ω-cntx-Pn1a (UniProt: O76201), U11-cntx-Pn1a (UniProt: P0C2S7), from *P*. *nigriventer* spider, as references. C) Group VIII alignment, using ω-cntx-Pn3a (UniProt: P81790), μ-cntx-Pn1a (UniProt: P17727), U20-cntx-Pn1a (UniProt: P84093) from *P*. *nigriventer* spider, as references. D) Group IX alignment, using ω-agtx-1A (UniProt: P15969), from *A*. *aperta* spider, as reference.

Groups VI and VII comprise sequences with 12 cysteine residues. Only two sequences were described for group VI (PN001 and PN300) and it is noteworthy that their cysteine framework has never been reported for *P*. *nigriventer* toxins (S3 Table, Fig 10A). Sequence PN001 has 64% identity to putative U9-agatoxin-Ao1a (UniProt: Q5Y4U3) from *Agelena orientalis* spider. Its sequence was confirmed by proteome, indicating it can be a novel toxin found in the venom. PN300 was classified in Type II/III omega-agatoxin family and is 43% similar to U20-ctenitoxin-Pn1a (UniProt: P84093), which is predicted to inhibit calcium channels.

Group VII is represented by 5 sequences, four of which belonging to Tx3-6 family. They are similar to ω-ctenitoxin-Pn4a (UniProt: P81792) from *P*. *nigriventer* and its isoforms U11-ctenitoxin-Pn1a (UniProt: P0C2S7) and U12-ctenitoxin-Pn1a (UniProt: P0C2S8) (S3 Table, Fig 10B). ω-ctenitoxin-Pn4a is a potent blocker of high voltage-activated calcium channels [92,93] and has demonstrated potent antinociceptive activity in mice [13,94,95]. PN313 has 76% identity with U12-ctenitoxin-Pn1a and was confirmed by proteomic approach, indicating that it is probably a novel toxin from the venom.

#### Group VIII (C-C-CXCC-CXC-CXC-C-C-C-C-C)

Eleven sequences were identified in this group of 14 cysteine residues and all of them matched (63–100% identity) with toxins from *P*. *nigriventer* (S3 Table, Fig 10C). As far as we know, no toxins with action on ion channels that exhibit such a high number of cysteine residues have been identified yet in the venom of any other spider. Sequences from this group were classified in the omega-agatoxin superfamily: Tx1 family and Type II/III omega-agatoxin family. ω-ctenitoxin-Pn3a (UniProt: P81790) is a calcium channel blocker and shows neuroprotective properties [9] and antinociceptive activity [11]. U20-ctenitoxin-Pn1a (UniProt: P84093) is also a predicted calcium channel inhibitor toxin and μ-ctenitoxin-Pn1a (UniProt: P17727) is a potent sodium channel inhibitor [96,97].

#### Group IX (C-C-CXCC-CXC-CXC-C)

This group comprises only one putative toxin sequence presenting a unique cysteine framework and was classified in Type I omega-agatoxin family. PN327 has 68% identity to ω-agatoxin-1A (UniProt: P15969) from *Agelenopsis aperta* spider, which is a blocker of L-type calcium channels (Cav/CACNA1) [98] (S3 Table, Fig 10D). This toxin has an unusual heterodimeric structure. Its precursor is cleaved to yield a major fragment that is covalently linked via disulfide bond to a smaller fragment of 3 residues (Ser-Pro-Cys) [99]. Sequence PN327 presents the same amino acids in the C-terminal portion and a similar cleavage motif flanked by two Arg residues. Thus, it may probably adopt the same heterodimeric structure. As this sequence was confirmed by proteome, it can be considered as a novel structural toxin sequence from *P*. *nigriventer* venom.

It is worthy to mention that the DDH motif, which is considered an ancestral ICK motif [100] as well as the Kunitz-type motif, which are present in mygalomorph spiders toxins [19,52], were not identified among the cysteine-rich peptides of *P*. *nigriventer*. It has been suggested that over the course of evolution, ICK polypeptides became predominant in spiders, reaching a large variety of structures, while the development of non-ICK polypeptide diversity was eliminated [63].

During the evolution of spiders, changes in venom composition allowed adaptation to different environments and the enormous diversity of toxins enabled them to become generalist predators. The results from this work confirmed that, as in other spider venoms, *Phoneutria nigriventer* venom is composed of an arsenal of cysteine-rich peptide toxins. It is noteworthy that these toxins are characterized by their sequence singularity, presenting low similarity with amino acid sequences of toxins previously described for other spider genera (S3 Table). Even toxins isolated from the spider species *Ctenus ornatus* and *Cupiennius salei* [54], which also belong to the Ctenidae family, have less than 70% identity when compared to sequences from *P*. *nigriventer* (S3 Table) Furthermore, the cysteine-rich peptide toxins identified in this work presented 6–14 cysteines in their sequence, showing a large diversity of cysteine frameworks when compared to other genera of Araneomorpha spiders such as *Lycosa or Dolomedes*, which present 8–12 cysteines [51,52,55,85]. Many of the cysteine-rich peptide toxins from *P*. *nigriventer* have predicted or characterized action on ion channels. As this spider is one of the few of medical importance in the world, the toxicity of its venom may be directly related to the great diversity of toxin sequences, which can have a synergistic effect on various types of ion channels, contributing to the toxicity of the venom.

#### Other protein families in *P*. *nigriventer* venom

In addition to cysteine-rich peptide toxins, *P*. *nigriventer* venom also comprises other components contributing to its toxic effects. The precise determination of whether a unique sequence identified by venom gland transcriptomic analysis represents indeed a venom component is a challenging task. For instance, digestive fluid is very often released during spider milking and hemolymph can also contaminate samples during venom gland extraction. Moreover, an overlap between venom proteins and some components of the spider’s digestive fluid has been reported [101].

It has been pointed out that venom toxins can originate from duplication of ordinary protein genes that undergo neofunctionalization in the venom gland but are still very similar to metabolism-acting molecules [50], making it difficult to distinguish whether a protein belongs to the venom or if it exerts cellular functions. Therefore, we considered as putative venom components from *P*. *nigriventer* venom glands those unique sequences that are similar to molecules that have already been identified in other venom composition analyses available in the literature; but comparative expression analyses with different tissues may be required for further confirmation [68].

The names of the families of molecules described as venom components were used to search among our annotated unique sequences from the NGS transcriptome database to retrieve the sequences that possibly belonged to these families. Using this methodology, we found 146 complete putative venom component sequences, divided into 27 different families. The presence of 16 of these putative toxin families was confirmed by proteomic analysis (Table 3), which does not necessarily mean they exert a toxic function. From these 16 families, three (lectins, serpins and chitinases) were validated by peptides matching incomplete sequences found in NGS. The unique sequences found were further manually analyzed and aligned with previously described toxins. The sequence alignments and the coverage of the peptides from the proteome are shown in S1 Appendix.

**Table 3:** List of the main families of molecules for the venom components found in NGS transcriptomic analysis.

	NGS			MudPIT		
Protein family	N° of sequences		FPKM	N° of sequences		PEPTIDE COVERAGE %*
	*complete*	*fragment*		***complete***	***fragment***	
CAPs	6	16	32329.30	3	1	56.1–44.0
Serine proteinases	13	16	26102.72	7	3	71.4–9.3
TCTP	2	1	7380.48	0	0	—-
Thyreoglobulin-domain inhibitors	5	4	4147.42	3	0	38.6–11.1
Leucin-rich repeat proteins	7	29	3085.13	2	3	26.3–18.0
TIL-domain inhibitors	9	2	2206.73	0	0	—-
Hyaluronidases	1	3	1679.34	1	0	42.5
Lectins	3	17	1258.76	0	1	—-
Lipases	16	18	594.70	2	0	3.9–2.0
Metalloproteinases	12	21	555.50	4	2	26.9–5.3
Cathepsins	5	14	474.63	1	0	1.6
Superoxide dismutases	2	3	416.42	2	0	7.5–6.6
Cystatins	3	1	393.72	1	0	9.4
ILGFB domain proteins	9	3	387.09	0	0	—-
Acetylcholinesterase	2	8	330.91	1	0	35.0
Kunitz inhibitors	5	7	221.82	0	0	—-
SERPINs	16	16	157.86	0	2	—-
Aminopeptidases	10	10	153.81	0	0	—-
Phospholipases	6	1	122.07	0	0	—-
Dopamine beta-hydroxylases	2	0	97.97	1	0	7.6
5' Nucleotidases	2	6	62.15	0	0	—-
Sphingomyelinases	1	4	58.91	0	0	—-
Waprins	1	0	57.54	0	0	—-
Chitinases	5	3	51.81	0	1	—-
Angiotensin-converting enzymes	1	1	37.44	1	0	14.1
Catalases	1	0	18.56	0	0	—-
Gamma-glutamyl transpeptidases	2	0	7.24	0	0	—-
TIMP inhibitors	1	0	4.77	0	0	—-

*% of peptide coverage refers only to complete sequences.

It is noteworthy that the proteomic analysis retrieved much less putative venom components then NGS: from the 146 complete sequences found in NGS, only 29 were also identified in MudPIT and from the 204 incomplete sequences of putative venom component sequences found in NGS, only 13 were confirmed by MudPIT. The percentage of peptide coverage of the sequences confirmed by MudPIT varied, but most of the sequences had less than 40% covered by peptides found in the proteomic analysis. Some groups (lipases, cathepsins, superoxide dismutases, cystatins, dopamine beta-hydroxilases) had less than 10% of peptide coverage, indicating that this proteomic technique, although capable of detecting a large number of peptides in complex mixtures, is limited. As MudPIT analyzes fragmented proteins, isoforms can generate similar peptides and the molecular diversity can be underestimated by this technique [102]. Similarly, other venomic studies have shown a small degree of overlapping between venom transcriptomic and proteomic analysis [68,103–107]. It is important to mention that, although several putative toxin transcripts were found in the venom gland by NGS, it is likely that not all of them are translated into proteins, since these two processes (transcription and translation) are subjected to different regulation and dynamics[105]. In addition, post-translational modifications can compromise the identification of the peptides obtained in the proteome [106].

After cysteine-rich peptide toxins, the members of CAP (Cysteine Rich Secretory Protein—CRiSP, antigen 5 and Pathogenesis-Related 1—PR-1) superfamily were the most abundant components found, representing 2.64% of putative venom components and 4.63% of unique venom component sequences found in *P*. *nigriventer* venom glands (Fig 2). Four CAP complete sequences had identity with a CAP described for *Trittame loki* barychelid spider (UniProt: W4VS53) [47], with high expression. Three of these sequences had fragments found in the proteomic analysis. The related peptides covered more than 40% of the sequences.

From the CAP family, CRISPs have been described as the most common members in venoms. A CRISP has already been identified in *P*. *keyserlingi* venom and a fragment of its sequence was submitted to UniProt (P85860), confirming the presence of this class of toxins in *Phoneutria* venoms. The contribution of CRISPs to venom toxicity, their exact molecular targets and mechanism of action remain unknown. The SCP (sperm-coating protein) domain, typical in these molecules and present in the identified sequences, may function as endopeptidases, which have been initially confirmed for Tex31, a CRISP found in *Conus textile* cone snail venom [108]. However, a subsequent work with a CRISP from *Conus marmoreus*, Mr30, which is highly similar to Tex31 but has low proteolytic activity, showed that this residual activity was due to contamination, making the initial results with Tex31 questionable [109,110]. This domain can also have a Ca^2+^ chelating function, acting on signaling processes and impairing channels and receptors that are sensitive to this ion. Using experimental approaches to unveil possible CRISPs actions in envenoming, smooth muscle contraction [111], inflammation [112], induction of expression of vascular endothelial cell adhesion molecules [113] and inhibition of angiogenesis [114] were observed, indicating that these molecules, besides having a role in envenoming, can also have a potential for biotechnological applications in the development of new drugs.

Serine proteases are the third most represented toxin family, accounting for 2.13% of abundancy among putative toxins and representing 6.39% of unique venom component sequences in *P*. *nigriventer* venom glands. Forty-two unique sequences were annotated as members of this family, 13 of them with complete sequences. Nine sequences presented high identity with U21-ctenitoxin-Pn1a (UniProt: P84033), a serine protease already described for *P*. *nigriventer* venom [18]; but none of them presented 100% of identity with it. The most abundant unique sequence (c21139_g1_i1) was also the most similar to U21-ctenitoxin-Pn1a, being 95.1% identical when excluding the signal peptide, which was not initially described for U21-ctenitoxin-Pn1a. Six sequences from these nine were confirmed by proteomic analysis, with variable peptide sequence coverage.

Serine proteases are very ubiquitous components in several venoms. Although they are vastly studied in snake venoms [115], they are also found in many arachnid venoms, as detected by both -omics and experimental approaches [42,116,117]. There are several hypotheses for the role of serine proteases in spider venoms. They may act in toxin maturation, prey digestion, hemostasis impairment [118] and in direct tissue damage [119], but further studies are needed to elucidate their exact role in *P*. *nigriventer* venom. It is not completely ruled out that these enzymes can also be part of digestive secretions or hemolymph components, present as contaminants in the venom gland transcriptome, but their relatively high expression detected in the present work points otherwise.

Translationally controlled tumor proteins (TCTP) were also found in our transcriptomic analysis in noticeable amounts. Although only three sequences matching this class of putative toxins were found, they represented 0.60% of the total venom components. *P*. *nigriventer* TCTPs sequences present high similarity (over 80%) with other molecules from this class, previously described for *Loxosceles intermedia* [120] and *Grammostola rosea* [121] spiders. Despite their remarkable presence in the venom gland transcriptome, TCTPs were not found in venom proteome.

TCTPs were initially described in human mammary carcinoma and seem to be related to histamine release and other physiological events, such as cell proliferation, cell death and tumor reversion [122]. TCTPs have also been widely detected in spider venoms and in transcriptomic analyses of other animal venom glands, but they have not been fully characterized [123]. A recombinant TCTP from *L*.*intermedia* spider venom is one of the few examples with a preliminary functional characterization. It induced paw edema when inoculated in mice and enhanced vascular permeability [120]. Indeed, venom TCTPs have been speculated to induce the local inflammatory reactions observed in envenomations, but further studies are required to confirm this evidence.

Altogether, the group of protease inhibitors accounted for 0.59% of the putative toxin abundance, and presented 15.42% of the unique venom component sequences from *P*. *nigriventer* venom gland. Different classes of protease inhibitors were found, such as inhibitors with thyroglobulin domain, TIL-type inhibitors, cystatins, kunitz-type inhibitors, and serpins. One unique sequence with high identity with whey acidic protein-type four-disulfide core domain proteins (WAP), known to act as elastase-specific inhibitors, and one unique sequence with high identity with tissue metalloprotease inhibitors (TIMP) were also identified in this work.

In addition to the previously described toxin families, other molecules that can potentially be part of *P*. *nigriventer* venom were found in the transcriptomic analysis, in smaller proportions. Leucin-rich repeat (LRR) proteins, hyaluronidases, lectins, metalloproteinases (including neprolysins, reprolysins and astacins), cathepsins, superoxide dismutases (SOD), insulin-like growth factor binding domain proteins (ILGFB), phospholipases, lipases, other putative toxins, such as defensins, SPRY domain-containing proteins, astakines, putative neurotoxins, acetylcholinesterase, aminopeptidases, angiotensin-converting enzyme, catalase, chitinase, gamma-glutamil transpeptidase, 5’ nucleotidase, sphingomyelinase, catalase, dopamine beta-hydroxylase are examples of annotations of other unique sequences found in the present analysis (S1 Appendix).

## Concluding remarks

Although the venom of the spider *P*. *nigriventer* has been studied for more than 40 years, this is the first study that provides a broad view of its components. In this work, conventional and next generation cDNA sequencing were combined with MudPIT proteomic analysis to unveil the molecular complexity of this venom. Transcriptomic and proteomic data showed that cysteine-rich peptide toxins are the most abundant component in this venom; several potential variants or isoforms of already described cysteine-rich peptide toxins, as well as novel ones of unknown function, were identified. The relative abundance of insecticide toxins is remarkable, suggesting that these toxins can have a significant role in the envenomation of natural preys. Moreover, many other components were identified in the venom, including CAPs, serine proteinases, proteinase inhibitors, metalloproteinases and hyaluronidases. It is noteworthy that a significant part of the unique sequences in the NGS transcriptome (63%) had no match with proteins deposited in UniProt. These sequences may constitute a valuable source of new molecules to be investigated and will require further efforts for functional validation. In summary, this study provided an overview of the composition of *P*. *nigriventer* spider venom, revealing a great venom complexity. These results can open new paths for further studies aiming at better understanding the molecular mechanisms of envenomation and unveiling novel molecules with potential biotechnological application.

## Supporting information

S1 Table Uniprot annotation of the top hundred FPKM values of unique sequences identified in *P*. *nigriventer* venom glands in NGS transcriptome. (PDF)

S2 Table Uniprot annotation of all peptides identified in *P*. *nigriventer* venom in MudPIT proteome. (XLSX)

S3 Table Summary of Cysteine-rich peptide toxins identified in the transcriptomic (CS and NGS) and proteomic analyses of venom glands and venom from *P*. *nigriventer*. (XLSX)

S1 Fig Gene Ontology annotation of all unique sequences identified in *P*. *nigriventer* venom glands NGS transcriptome. Unique sequences were placed in different categories, in the three GO namespaces. Graphs show the number of unique sequences annotated for each GO category (BP, CC, MF).
(TIF)

S1 Appendix Sequence alignments of ‘other venom components’ unique sequences identified in NGS transcriptome. (PDF)
