# Supplementary material for: An overview of Phoneutria nigriventer spider venom using combined transcriptomic and proteomic approaches
Source: PLoS One. 2018 Aug 1;13(8):e0200628. doi: 10.1371/journal.pone.0200628 (PMC6070231; doi:10.1371/journal.pone.0200628)
Supplement: S1 Table — (PDF) [file pone.0200628.s001.pdf]

Cellular function protein
Venom component
No match
Uncharacterized spider protein

	FPKM	Uniprot.blastx	Organism	Uniprot ID	E-value
**1**	325098,27	ɣ-ctenitoxin-Pn1a	*Phoneutria nigriventer*	P59367	2E-50
**2**	144132,72	ɣ-ctenitoxin-Pn1a	*Phoneutria nigriventer*	P59367	7E-50
**3**	70294,28	U2-ctenitoxin-Pn1b	*Phoneutria nigriventer*	O76198	1E-72
**4**	64930,74	ω-ctenitoxin-Pn4a	*Phoneutria nigriventer*	P81792	2E-57
**5**	62699,96	U19-ctenitoxin-Pn1a	*Phoneutria nigriventer*	P83997	3E-36
**6**	59828,71	U9-agatoxin-Ao1a	*Agelena orientalis*	Q5Y4U3	6E-32
**7**	59228,83	putative neurotoxin LTDF S-05	*Dolomedes fimbriatus*	A0A0K1D9C4	1.5E-45
**8**	51574,28	δ-ctenitoxin-Pn1a	*Phoneutria nigriventer*	P59368	2E-33
**9**	50035,59	Uncharacterized protein	*Dolomedes mizhoanus*	S5MFK1	6.5E-20
**10**	34692,12	δ-ctenitoxin-Pn2a	*Phoneutria nigriventer*	P29425	2E-49
**11**	34267,04	ω-lycotoxin-Gsp2671d	*Lycosa kazakhstanicus*	A9XDG2	3E-11
**12**	26641,87	u-ctenitoxin-Pn1a	*Phoneutria nigriventer*	P17727	2E-75
**13**	26622,61	U2-ctenitoxin-Pn1a	*Phoneutria nigriventer*	O76198	7E-64
**14**	25200,89	Uncharacterized protein	*Dolomedes sulfureus*	A0A0P0D5G5	9E-22
**15**	23351,8	U20-lycotoxin-Ls1d	*Lycosa singoriensis*	B6DCY1	2E-09
**16**	23053,16	U11-lycotoxin-Ls1d	*Lycosa singoriensis*	B6DD12	1E-09
**17**	22418,68	U12-ctenitoxin-Pn1a	*Phoneutria nigriventer*	P0C2S8	3E-66
**18**	21871,01	U21-ctenitoxin-Pn1a	*Phoneutria nigriventer*	P84033	9E-167
**19**	20119,75	ω-ctenitoxin-Pn1a	*Phoneutria nigriventer*	O76201	2E-51
**20**	19918,62	U2-ctenitoxin-Pn1b	*Phoneutria nigriventer*	P29423	6E-49
**21**	19728,49	U6-lycotoxin-Ls1f	*Lycosa singoriensis*	B6DCV6	1E-09
**22**	19354,24	no match	*x*	no match	null
**23**	15598,8	Venom allergen 5	*Lycosa singoriensis*	A9QQ26	3E-134
**24**	14132,62	Clone 939 transcribed RNA sequence	*Plectreurys tristis*	A0A0C4W9T3	9E-06
**25**	13933,08	U4-ctenitoxin-Pk1a (Fragment)	*Phoneutria keyserling*	P83896	2E-18
**26**	11322,17	BLTX179	*Nephila pilipes*	A0A076KZ31	6E-39
**27**	9803,23	U7-ctenitoxin-Pn1a	*Phoneutria nigriventer*	P81791	3E-03
**28**	9511,18	Actin 5C	*Lycosa singoriensis*	A9QQ31	0E+00
**29**	9291,31	Uncharacterized protein	*Stegodyphus mimosarum*	A0A087TLL1	4E-25

**30**	8548,47	CRISP/Allergen/PR-1	*Trittame loki*	W4VS53	2E-101
**31**	8509,12	U11-ctenitoxin-Pn1a	*Phoneutria nigriventer*	P0C2S8	8E-49
**32**	8069,09	Uncharacterized protein	*Cerapachys biroi*	A0A131XBK2	3E-01
**33**	8027,26	CRISP/Allergen/PR-1	*Trittame loki*	W4VS53	3E-97
**34**	7533,39	U4-agatoxin-Ao1a	*Agelena orientalis*	Q5Y4U5	8E-16
**35**	7532,87	Stress-associated endoplasmic reticulum protein 2	*Mus musculus*	Q6TAW2	5E-27
**36**	7531,31	U7-ctenitoxin-Pn1a	*Phoneutria nigriventer*	P81791	3E-04
**37**	6796,38	no match NO ORF	*x*	null	null
**38**	6672,74	Uncharacterized protein	*Stegodyphus mimosarum*	A0A087TUH2	1E-81
**39**	6590,6	Translationally-controlled tumor protein homolog	*Grammostola rosea*	M5B4R7	1E-106
**40**	6501,91	Calcyphosin-like protein	*Mus musculus*	Q6P8Y1	1E-61
**41**	6062,37	Tropomyosin	*Dermatophagoides farinae*	Q23939	4E-159
**42**	6047,32	Uncharacterized protein	*Stegodyphus mimosarum*	A0A087TS45	6E-21
**43**	5171,47	ω-ctenitoxin-Pn3a	*Phoneutria nigriventer*	P81790	2E-62
**44**	5079,67	ω-ctenitoxin-Pn3a	*Phoneutria nigriventer*	P81790	3E-68
**45**	4982,7	Kappa-ctenitoxin-Pn1a	*Phoneutria nigriventer*	O76200	7E-42
**46**	4688,13	Putative neurotoxin LTDF S-19	*Dolomedes fimbriatus*	A0A0K1D8Z3	2E-06
**47**	4621,42	U6-lycotoxin-Ls1f	*Lycosa singoriensis*	B6DCV6	6E-09
**48**	4580,74	no match	*x*	null	null
**49**	4334,86	Transposable element Tc3 transposase	*Caenorhabditis elegans*	P34257	5E-33
**50**	4298,83	ω-agatoxin-1A	*Agelenopsis aperta*	P15969	9E-36
**51**	4273,26	Putative neurotoxin LTDF S-05	*Dolomedes fimbriatus*	A0A0K1D9C4	7E-55
**52**	4229,17	Uncharacterized protein	*Stegodyphus mimosarum*	A0A087TLL1	5.5E-16
**53**	4197,39	Soma ferritin	*Lymnaea stagnalis*	P42577	3E-88
**54**	4066,72	U24-ctenitoxin-Pn1a	*Phoneutria nigriventer*	P84032	1E-22
**55**	4021,75	U20-ctenitoxin-Pn1a (Fragment)	*Phoneutria nigriventer*	P84093	2E-20
**56**	3857,24	Arginine kinase	*Limulus polyphemus*	P51541	0E+00
**57**	3804,81	U10-ctenitoxin-Pn1a (Fragment)	*Phoneutria nigriventer*	P0C2S9	6E-42
**58**	3743,06	Orcokinin peptides type A	*Procambarus clarkii*	Q9NL83	9E-15
**59**	3481,69	Calcyphosin-like protein	*Mus musculus*	Q6P8Y1	1E-62
**60**	3325,84	60S acidic ribosomal protein P1	*Drosophila melanogaster*	P08570	2E-32
**61**	3301,51	no match	*x*	null	null
**62**	3176,55	U7-ctenitoxin-Pn1a	*Phoneutria nigriventer*	P81791	4E-52
**63**	3132,53	Nucleoside diphosphate kinase	*Drosophila melanogaster*	P08879	1E-82

**64**	3077,85	60S ribosomal protein L29	*Macaca fascicularis*	Q8HXB8	1E-19
**65**	3052,34	Actin-3	*Limulus polyphemus*	P41340	3E-113
**66**	3010,68	Toxin-like structure LSTX-D2	*Lycosa singoriensis*	B6DCU1	1E-31
**67**	2999,51	Techylectin-like protein (Fragments)	*Phoneutria nigriventer*	P85031	3E-138
**68**	2977,25	no match	*x*	null	null
**69**	2970,42	U14-ctenitoxin-Pn1a	*Phoneutria nigriventer*	P83998	6E-18
**70**	2949,59	Myosin regulatory light chain 2	*Bombyx mori*	Q1HPS0	8E-54
**71**	2945,68	U10-ctenitoxin-Pn1a (Fragment)	*Phoneutria nigriventer*	P0C2S9	9E-24
**72**	2831,44	ADP/ATP translocase 3	*Bos taurus*	P32007	0E+00
**73**	2830,24	Troponin I	*Astacus leptodactylus*	P05547	5E-44
**74**	2786,07	Protein translation factor SUI1 homolog	*Anopheles gambiae*	P42678	2E-54
**75**	2667,25	Muscle LIM protein 1	*Drosophila melanogaster*	Q24400	3E-33
**76**	2624,9	Leucine-rich repeat and immunoglobulin-like domain-containing nogo receptor-interacting protein 1	*Xenopus tropicalis*	A4IIW9	3E-12
**77**	2544,9	Glutathione peroxidase 3	*Homo sapiens*	P22352	5E-50
**78**	2477,58	U8-theraphotoxin-Hhn1f	*Haplopelma hainanum*	D2Y2E6	5E-19
**79**	2466,43	U20-ctenitoxin-Pn1a (Fragment)	*Phoneutria nigriventer*	P84093	3E-33
**80**	2449,05	Protein disulfide-isomerase	*Gallus gallus*	P09102	0E+00
**81**	2381,63	δ-ctenitoxin-Pn2a	*Phoneutria nigriventer*	P29425	8E-50
**82**	2380,78	U6-ctenitoxin-Pn1a	*Phoneutria nigriventer*	P81793	1E-50
**83**	2333,68	Uncharacterized protein	*Stegodyphus mimosarum*	A0A087T5I3	7E-11
**84**	2332,46	U9-ctenitoxin-Pn1a	*Phoneutria nigriventer*	P0C2S6	5E-11
**85**	2267,32	U9-ctenitoxin-Pn1a	*Phoneutria nigriventer*	P0C2S6	1E-44
**86**	2241,66	Nuclear protein 1	*Rattus norvegicus*	O54842	2E-06
**87**	2175,23	Transposable element Tcb2 transposase	*Caenorhabditis briggsae*	Q04202	5E-10
**88**	2164,77	U10-ctenitoxin-Pn1a (Fragment)	*Phoneutria nigriventer*	P0C2S9	7E-25
**89**	2146,66	Putative RNA-directed DNA polymerase from transposon BS	*Stegodyphus mimosarum*	A0A087SVY8	2E-15
**90**	2145,4	U11-lycotoxin-Ls1d	*Lycosa singoriensis*	B6DD10	5E-09
**91**	2059,21	U13-ctenitoxin-Pn1b	*Phoneutria nigriventer*	P84017	1E-11
**92**	1982,5	Serine/arginine-rich splicing factor 7	*Bos taurus*	Q3T106	8E-20
**93**	1957,18	Chymotrypsin-elastase inhibitor ixodidin	*Rhipicephalus microplus*	P83516	2E-10
**94**	1903,85	ω-lycotoxin-Gsp2671c	*Lycosa kazakhstanicus*	A9XDG1	6E-13
**95**	1881,65	U10-ctenitoxin-Pn1a (Fragment)	*Phoneutria nigriventer*	P0C2S9	5E-19

**96**	1865,32	Protein CDV3-like protein	*Stegodyphus mimosarum*	A0A087UFN6	1E-118
**97**	1846,83	no match	*x*	null	null
**98**	1804,88	Multiple coagulation factor deficiency protein 2 homolog	*Pongo abelii*	Q5R8Z6	2E-11
**99**	1766,92	Apolipoprotein D	*Cavia porcellus*	P51909	1E-23
**100**	1762,22	no match NO ORF	*x*	null	null
