# Supplementary figures and images for: An overview of Phoneutria nigriventer spider venom using combined transcriptomic and proteomic approaches
Source: PLoS One. 2018 Aug 1;13(8):e0200628. doi: 10.1371/journal.pone.0200628 (PMC6070231; doi:10.1371/journal.pone.0200628)

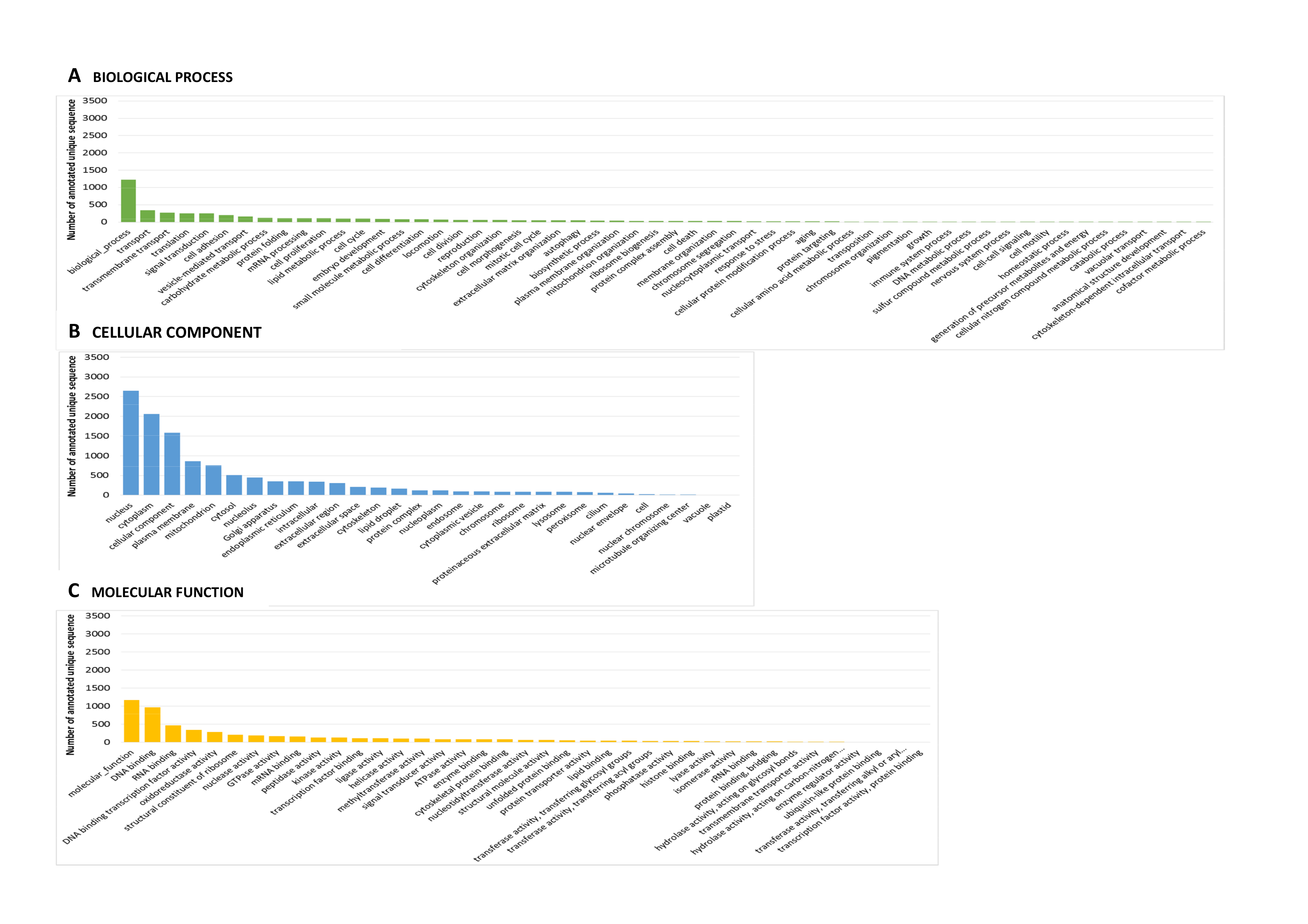

Supplement: S1 Fig — Unique sequences were placed in different categories, in the three GO namespaces. Graphs show the number of unique sequences annotated for each GO category (BP, CC, MF). (TIF) [file pone.0200628.s004.tif]
