# Supplementary material for: An overview of Phoneutria nigriventer spider venom using combined transcriptomic and proteomic approaches
Source: PLoS One. 2018 Aug 1;13(8):e0200628. doi: 10.1371/journal.pone.0200628 (PMC6070231; doi:10.1371/journal.pone.0200628)
Supplement: S1 Appendix — (PDF) [file pone.0200628.s005.pdf]

# SUPPORTING INFORMATION – APPENDIX

Other venom components alignment

# CAP

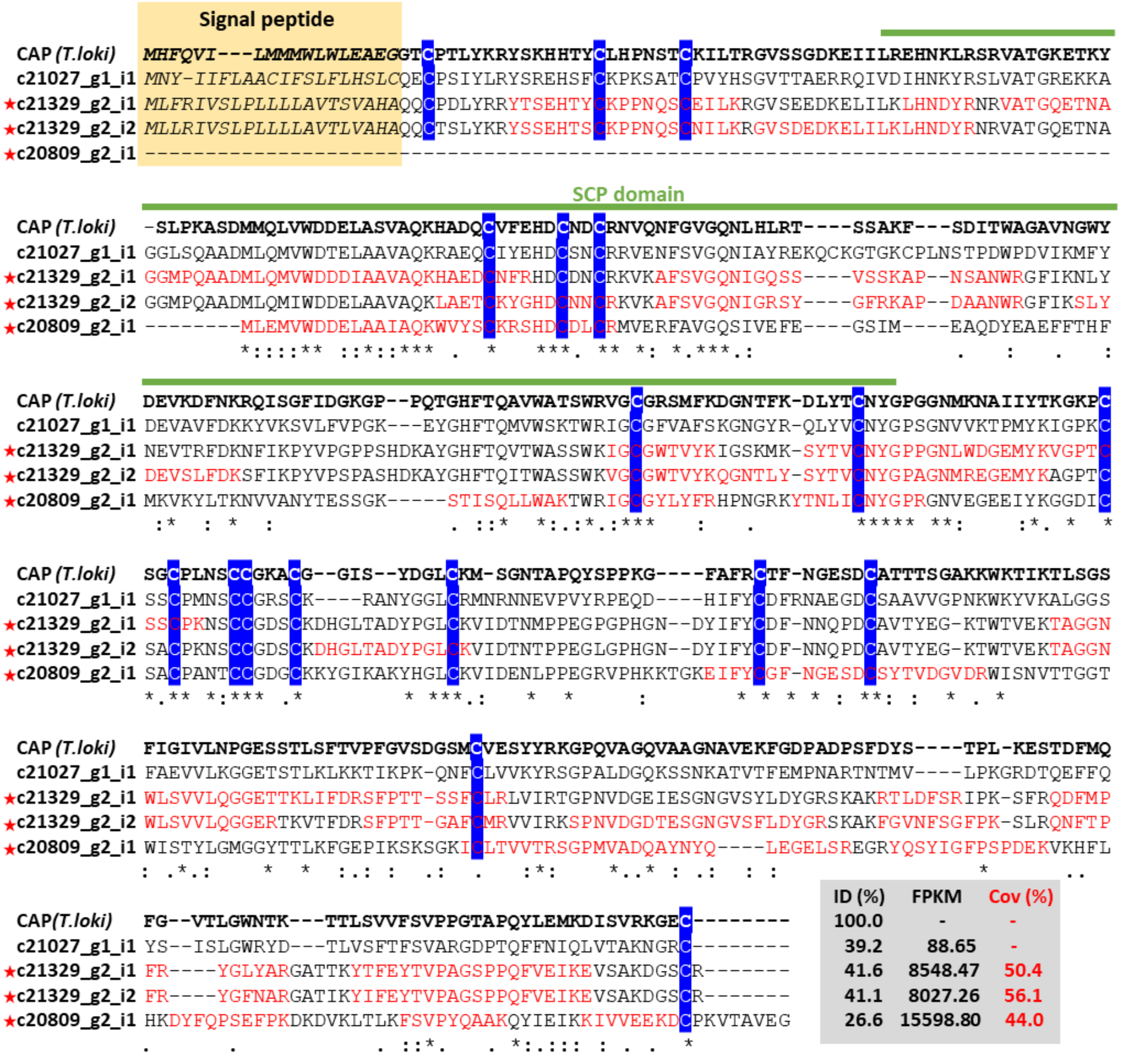


**Fig A1: Sequence alignment of transcripts pertaining to the CAP family.** Alignment was performed with MUSCLE, using CAP-1 protein, from *Trittame loki* spider (UNIPROT W4VS53), as a reference. Signal peptide is highlighted in yellow and conserved cysteines are marked in blue. The green line marks the conserved SCP domain. Sequences marked by red stars were confirmed in the proteome and amino acid residues in red correspond to the peptide sequences obtained by MudPIT analysis. Percentage of identity (ID%) with the reference protein was calculated using the tool EMBOSS Stretcher for pairwise sequence alignment. FPKM shows the transcript abundancy. Peptide coverage (Cov %) shows the percentage of the original transcript sequence confirmed by the proteome.

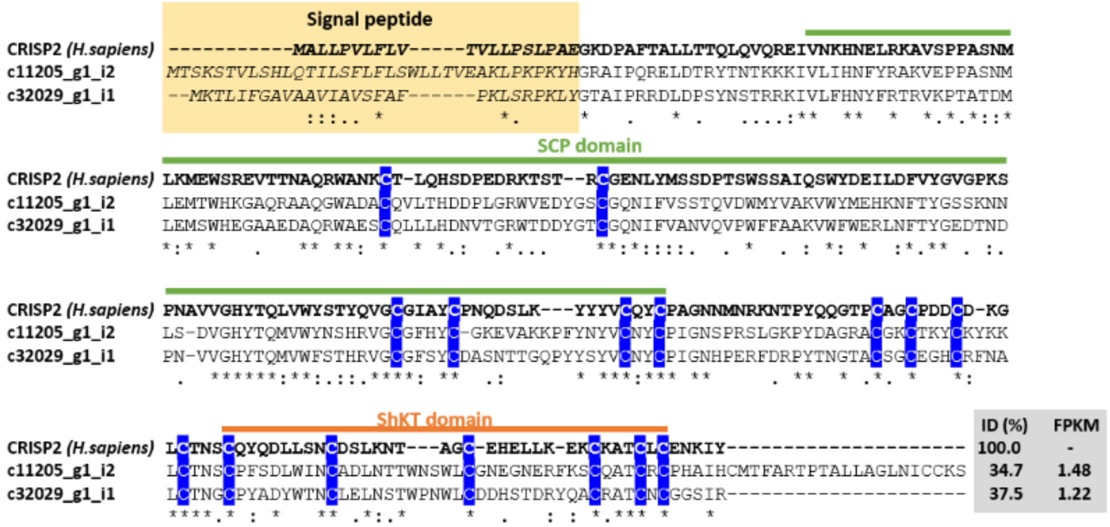


**Fig A2: Sequence alignment of transcripts similar to human CRISP2.** Alignment was performed with MUSCLE, using human CRISP2 protein (UNIPROT P16562), as a reference. Signal peptide is highlighted in yellow and conserved cysteines are marked in blue. The green line marks the conserved SCP domain and orange line marks th ShKT domain. Percentage of identity (ID%) with the reference protein was calculated using the tool EMBOSS Stretcher for pairwise sequence alignment. FPKM shows the transcript abundancy.

# SERINE PROTEINASE

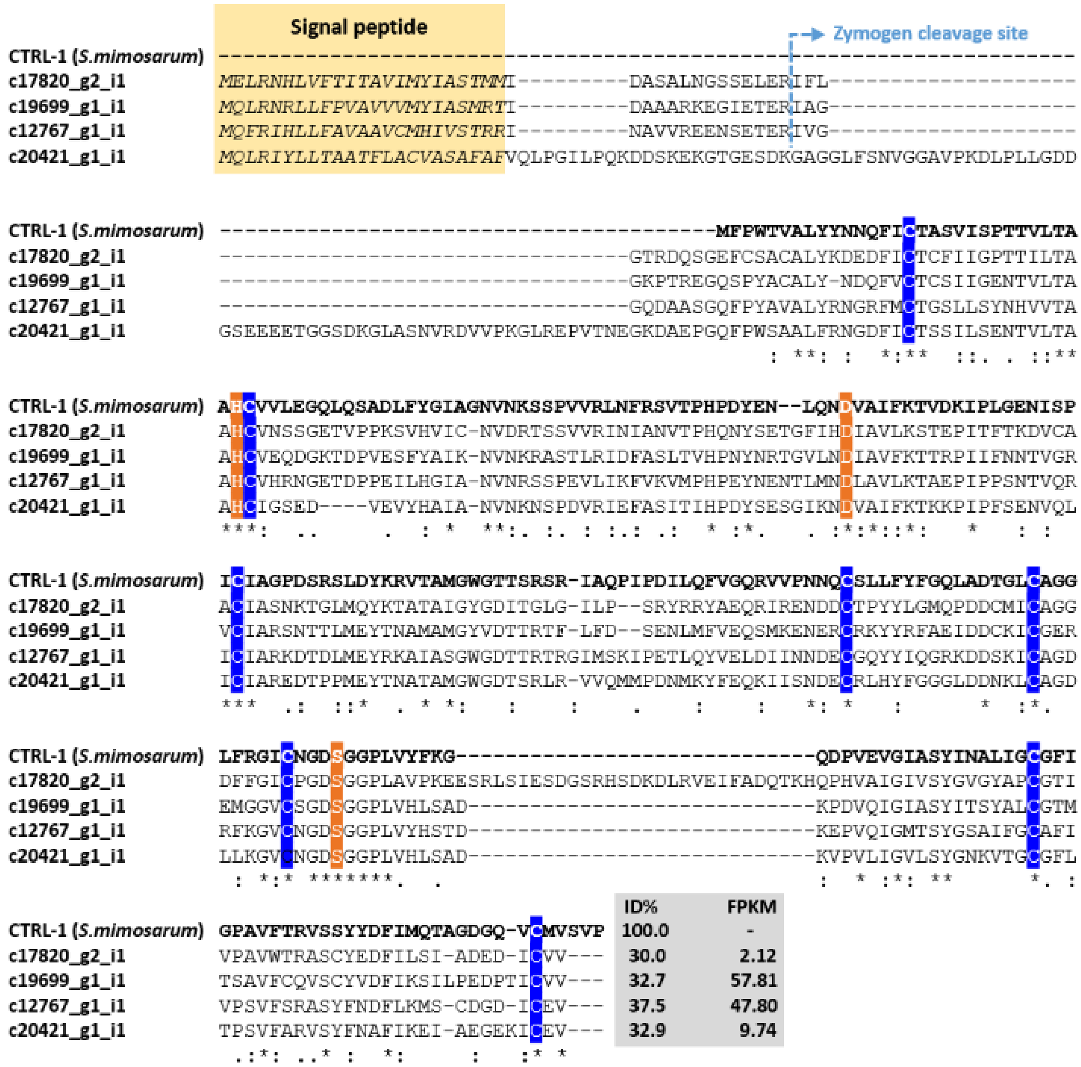


**Fig A3: Sequence alignment of transcripts similar to serine protease chymotrypsin-like 1.** Alignment was performed with MUSCLE, using chymotrypsin-like protease CTRL-1, from spider Stegodyphus mimosarum (UNIPROT A0A087U6A9), as a reference. Signal peptide is highlighted in yellow. The zymogen cleavage site is marked with a doted light blue line. Conserved catalytic triad is highlighted in orange and conserved cysteines are highlighted in blue. Percentage of identity (ID%) with the reference protein was calculated using the tool EMBOSS Stretcher for pairwise sequence alignment. FPKM shows the transcript abundancy.

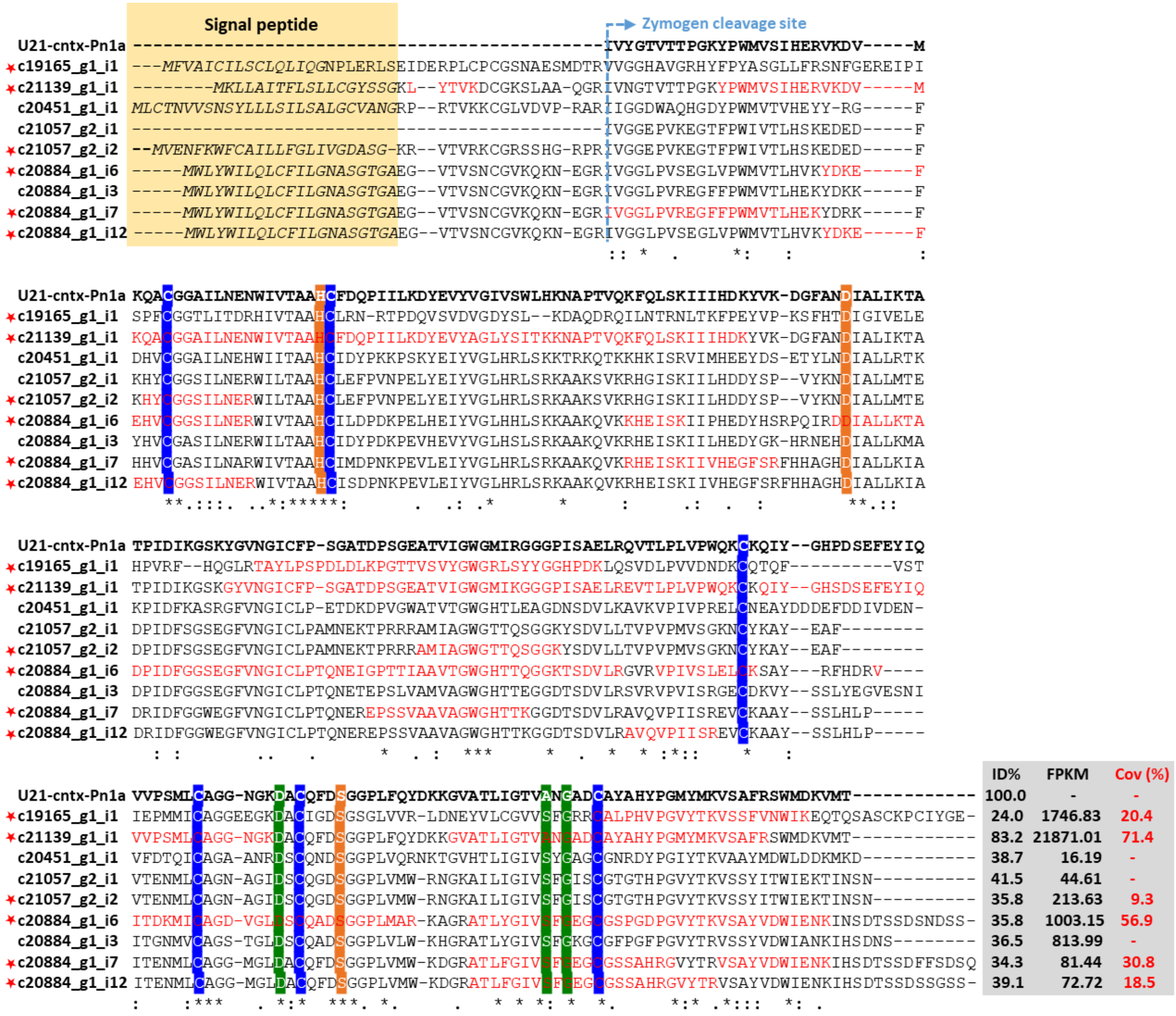

**Fig A4: Sequence alignment of transcripts similar to serine protease U21-ctenitoxin-Pn1a**. Alignment was performed with MUSCLE, using U21-ctenitoxin-Pn1a, from Phoneutria nigriventer (UNIPROT P84033), as a reference. Signal peptide is highlighted in yellow. The zymogen cleavage site is marked with a doted light blue line. Conserved catalytic triad is highlighted in orange and substrate binding site is highlighted in green. Conserved cysteines are highlighted in blue. Sequences marked by red stars were confirmed in the proteome and amino acid residues in red correspond to the peptide sequences obtained by MudPIT analysis. Percentage of identity (ID%) with the reference protein was calculated using the tool EMBOSS Stretcher for pairwise sequence alignment. FPKM shows the transcript abundancy. Peptide coverage (Cov %) shows the percentage of the original transcript sequence confirmed by the proteome.

# TRANSLATIONALLY CONTROLLED TUMOR PROTEIN

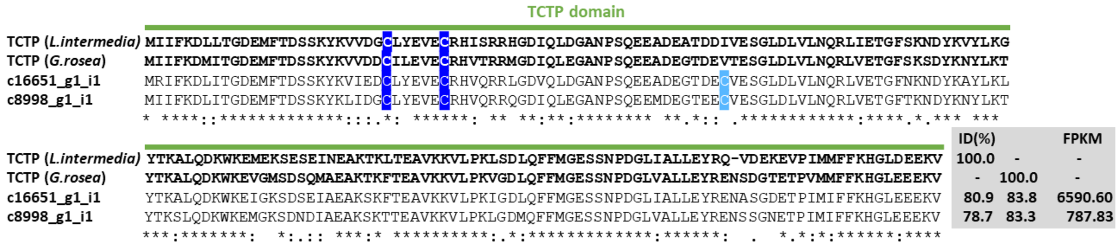


**Fig A5: Sequence alignment of transcripts similar to TCTPs.** Alignment was performed with MUSCLE, using TCTP from *Loxosceles intermedia* (UNIPROT: G3LU44*)* and *Grammostola rosea* (UNIPROT: M5B4R7), as references. The conserved cysteines are highlighted in blue and non-conserved cysteines are in cyan. The green line marks the conserved TCTP domain. Percentage of identity (ID%) with the reference proteins was calculated using the tool EMBOSS Stretcher for pairwise sequence alignment. FPKM shows the transcript abundancy.

# THYREOGLOBULIN-DOMAIN INHIBITORS

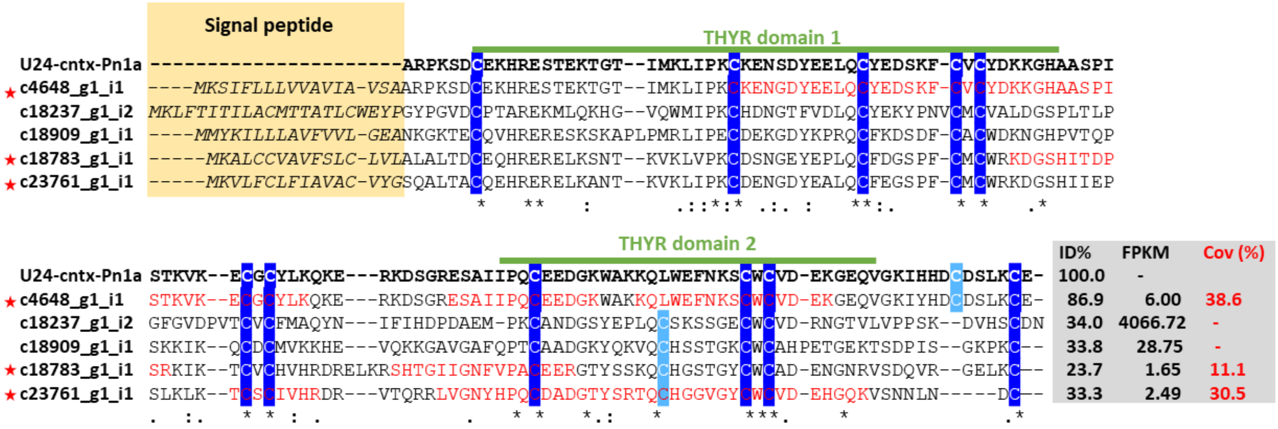


**Fig A6: Sequence alignment of transcripts similar to U24-ctenitoxin-Pn1a.** Alignment was performed with MUSCLE, using U24-ctenitoxin-Pn1a (UNIPROT P84032), from *Phoneutria nigriventer*, as references. Signal peptide is highlighted in yellow. The conserved cysteines are highlighted in blue and non-conserved cysteines are in cyan. The green line marks the conserved thyreoglobulin domain. Sequences marked by red stars were confirmed in the proteome and amino acid residues in red correspond to the peptide sequences obtained by MudPIT analysis. Percentage of identity (ID%) with the reference protein was calculated using the tool EMBOSS Stretcher for pairwise sequence alignment. FPKM shows the transcript abundancy. Peptide coverage (Cov %) shows the percentage of the original transcript sequence confirmed by the proteome.

# LEUCIN RICH REPEAT PROTEINS

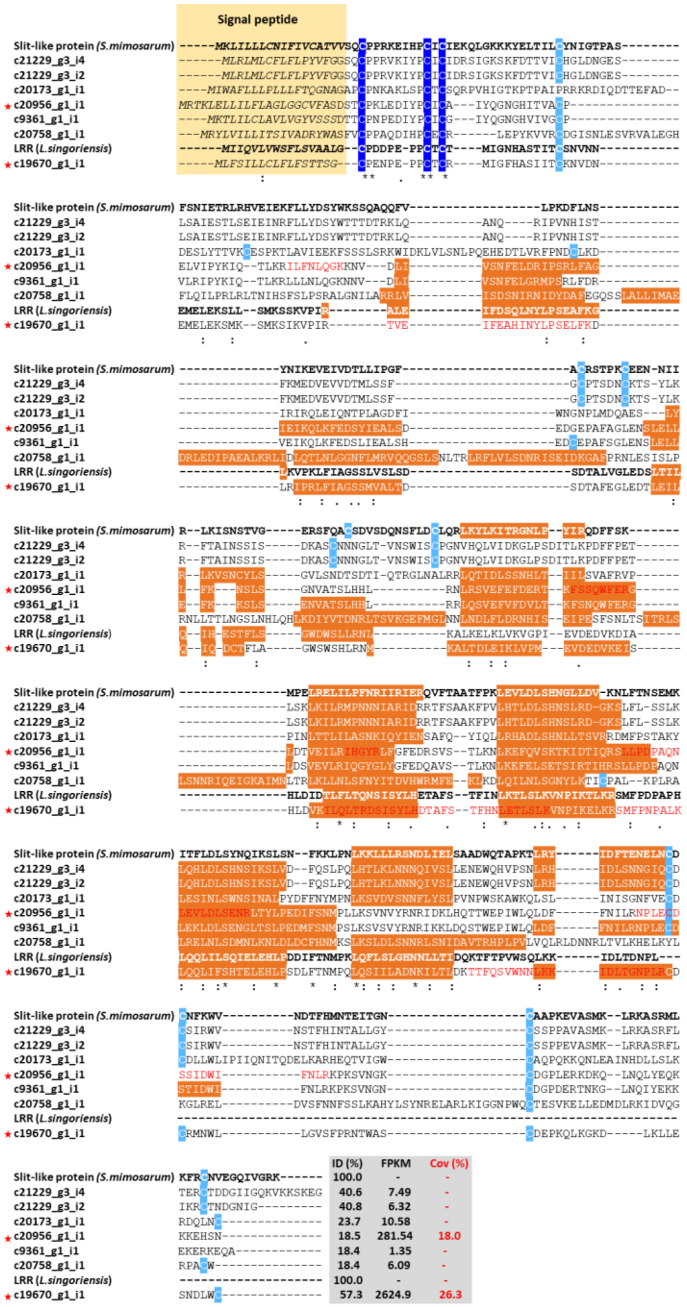


**Fig A7: Sequence alignment of transcripts containing leucin-rich repeats (LRR) domain.** Alignment was performed with MUSCLE, using Slit-like protein, from spider *Stegodyphus* (UNIPROT A0A087UVA6) and LRR from spider *Lycosa singoriensis* (UNIPROT A9QQ71) as references. Signal peptide is highlighted in yellow. Residues that compose LRR repeats are highlighted in orange. Conserved cysteines are highlighted in blue and non-conserved ones are in cyan. Sequences marked by red stars were confirmed in the proteome and amino acid residues in red correspond to the peptide sequences obtained by MudPIT analysis. Percentage of identity (ID%) with the reference protein was calculated using the tool EMBOSS Stretcher for pairwise sequence alignment. FPKM shows the transcript abundancy. Peptide coverage (Cov %) shows the percentage of the original transcript sequence confirmed by the proteome

# TIL-DOMAIN INHIBITORS

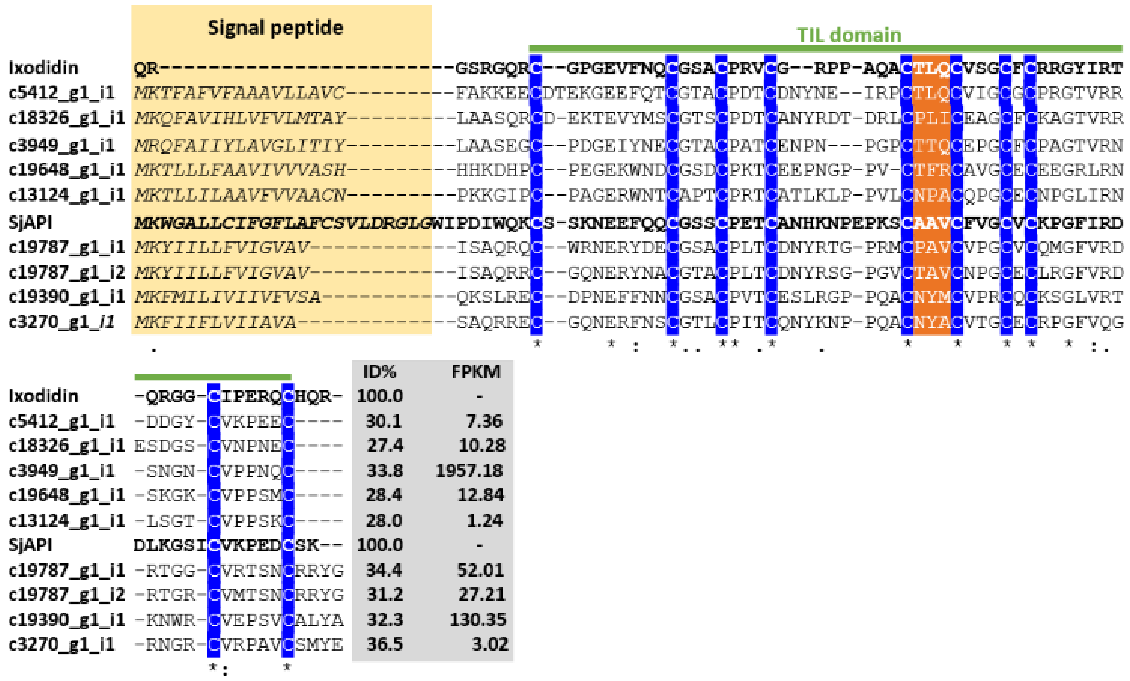


**Fig A9: Sequence alignment of transcripts with Trypsin Inhibitor-like (TIL) domain.** Alignment was performed with MUSCLE, using chymotrypsin-elastase inhibitor ixodidin (UNIPROT P83516), from *Rhipicephalus microplus* and SjAPI (UNIPROT P0DM55), from *Scorpiops jendeki* as references. Signal peptide is highlighted in yellow. The conserved cysteines are highlighted in blue. Protease binding loop is highlighted in orange The green line marks the conserved TIL domain. Percentage of identity (ID%) with the reference proteins was calculated using the tool EMBOSS Stretcher for pairwise sequence alignment. FPKM shows the transcript abundancy.

# HYALURONIDASE

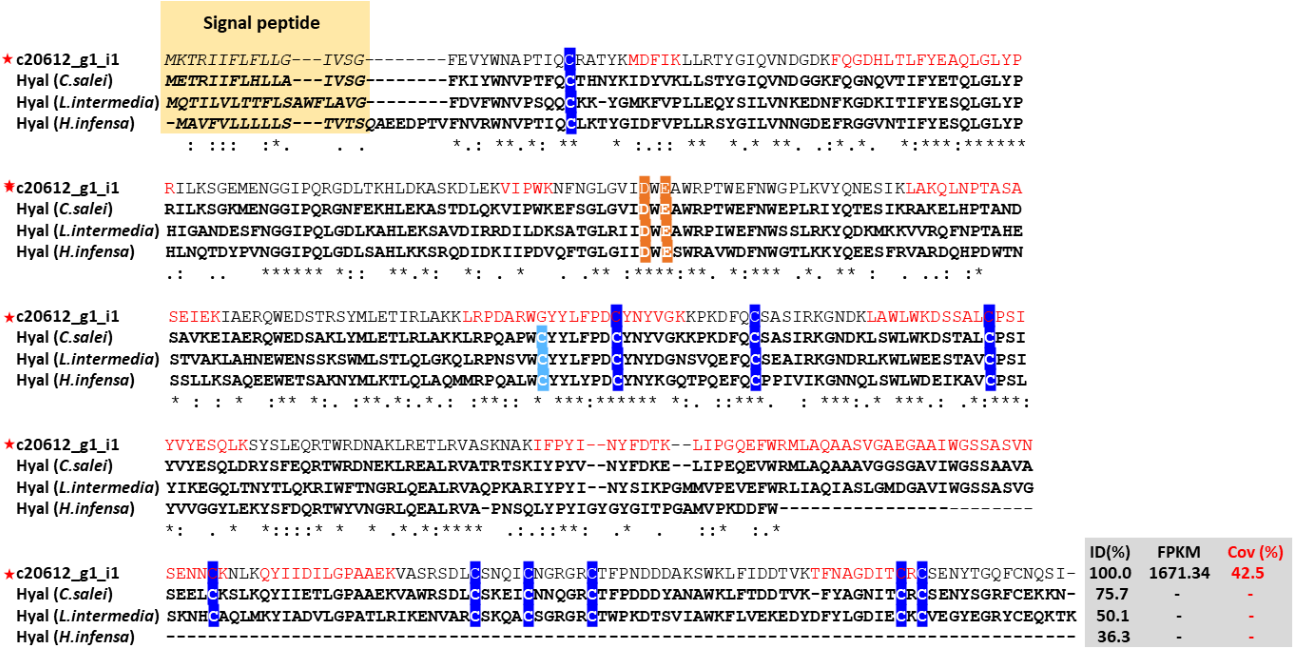


**Fig A10: Sequence alignment of transcripts similar to hyaluronidase.** Alignment was performed with MUSCLE, using hyaluronidases from spider *Cupiennius salei* (UNIPROT A0A0S4JYH2), *Loxosceles intermedia* (UNIPROT R4J7Z9) and *Hadronyche infensa* (UNIPROT A0A1D0C0T3) as references. Signal peptide is highlighted in yellow. Residues important for catalysis are highlighted in orange. Conserved cysteines are highlighted in blue and non-conserved ones are in cyan. Sequences marked by red stars were confirmed in the proteome and amino acid residues in red correspond to the peptide sequences obtained by MudPIT analysis. Percentage of identity (ID%) with the reference protein was calculated using the tool EMBOSS Stretcher for pairwise sequence alignment. FPKM shows the transcript abundancy. Peptide coverage (Cov %) shows the percentage of the original transcript sequence confirmed by the proteome.

# LECTINS

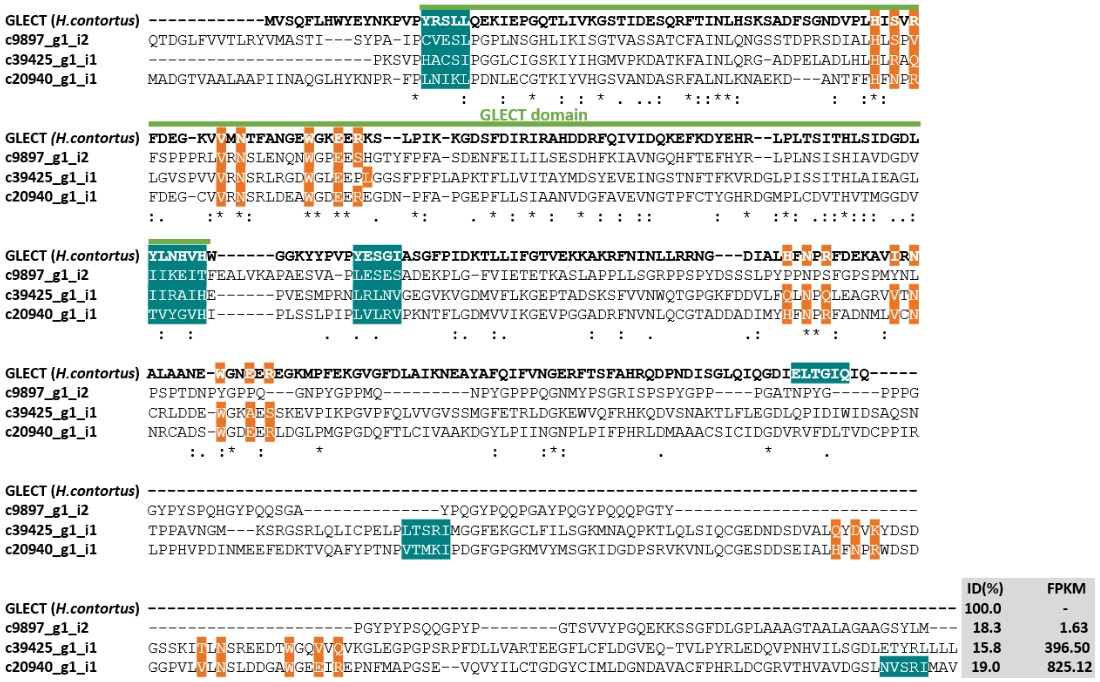


**Fig A11: Sequence alignment of transcripts similar to Galectin/galactose-binding lectins (GLECT).** Alignment was performed with MUSCLE, using galectin from nematode *Haemonchus contortus* (UNIPROT O44126), as reference. Residues composing the sugar binding pocket are highlighted in orange. Residues highlighted in dark green are part of dimerization swapping strand. The green line marks the common GLECT domain. Percentage of identity (ID%) with the reference proteins was calculated using the tool EMBOSS Stretcher for pairwise sequence alignment. FPKM shows the transcript abundancy.

# LIPASES

```
                         Signal peptide
ABH-DCP 11 (S.mimosarum) -------MFLPGVI-CFIFFLGISTSVPIDAPKITPVDLSYTCIQVTLGEQPEEKAPVILIHGVLA-----SKEIWR
c19801_g1_i1             -----------------METVQLAYDVYES-----------------GETQGAGKCPIILLHGLFW-----NKYMLR
c20557_g1_i1             MNVRYFFRISQKMRTCYKSTLLRPVCFSVSSQDFCSTKPVKLSFTEFQPDKISNSIPVVILHGFLG-----SKQNWR
c18622_g1_i2             -------MFLLGIVLAVLAFPDVTFSAPIEKEIIEPVDLYYQCYHV-VQEIESNKTPILLIHGLGG-----SHNAWR
c20594_g1_i1             -----------------------------------------------------------------------------
c20470_g1_i3             --------------------MKLAYETFL------------------PADEELDENPVIMLHCAFD-----SGKTWY
c18404_g1_i2             ------EELILSQLDYYSVQNKGQWVVPEH-----------------PGERYDGWKFCSFLARSLQLRIATSSSFVQ
c6245_g2_i1              -----------------------------------------------------------------------------
c19629_g1_i1             --------------------MKLAFDVYAP-----------------PGREDSGLSPVILLHGRLD-----SRKTWK

                                                    AB-hydrolase domain
ABH-DCP 11 (S.mimosarum) GFQEHLSLKTGRKVCAPDLRNHGDSSWSDRTDIAAMAVDSVELMDNLRMSKAVLLGLSLGGKVAVHVSLENPQRVEK
c19801_g1_i1             DLAKALCSATQRKVFCLDLRCHDESPFREDCDADRMAEDVKRFVRERKLEKVAFVCHSFSCTVAYLVVVDQPETVEK
c20557_g1_i1             GLGKAIAKKSGRQVFALDARNHGDSPHSDDFSYTTMCEDVKAFLSSQAISRAVLVGHSMGGRTAMKLSLTEKQMVNK
c18622_g1_i2             GVSQVLALQTARRVCAVDLRNHGDSPWNNRTDAEALAKDIGNFLDKQKIKKVNLVGHSLGGKTAIHYTLNNPDRVDS
c20594_g1_i1             -MPQHVANVTKRTVYTVDARNHGESEWSDHFNFDVNADDLMHFMDIRGIKKAVIFGHSMGGLTAIKTALRWPDRVDK
c20470_g1_i3             KVAPIIADITGRKVFALDARNHGDSSRSVNMSWSLLVEDLEEFLEDHTIQKVILIGHGMGGTTALAFALSKPERVEK
c18404_g1_i2             VVRRSETLLNYTTVYAVDARNHGESPWDDEMDVKVLIEDLDEFLTDRNVSKSVLVGHCFGGFIAMAYAIHKPDRVEK
c6245_g2_i1              -IAPNLSRNTGRIVYVVDERNHGSSPRSNGMDWAVLADDIGDFLRQHGIQKAVLVGHSMGGRASFTFALKHPEMVEK
c19629_g1_i1             KLAPRIAEKTGRKVYAYDARNHGESPWTPEMDLQILSNDLDQFLKDHAIEKAVLLGHSMGGKTAIVFSLQKPQKVEK
                          .       .   *   * * *..*      .      *   ::    :..  :.   :.   :    :   : *:.

ABH-DCP 11 (S.mimosarum) VIVGDMR--PNGVSKGALGNMKATVALLNESLKIIPENADDKSAKKAVL-AFINSQLKKANKTELLKESDAD----L
c19801_g1_i1             VVMIDHPPYPDYTDQFYDDNVHPQFVAQNRFLKTLDPSLSLTAAKKKIL--SLSKGASEESHRIFLRKI-------A
c20557_g1_i1             LIVVDVS--PIKMPESSSIFIPSYMVAMKEAIAFI-HDVGLVQARKLID-EYLSKTIPEVAIRQFL----------L
c18622_g1_i2             LVVEDMR--PNGATESSIILVKTVLKYLQEAIQTVPEGVNEQEAKSTII-KFLNKKTQELGSTDTIDENT------E
c20594_g1_i1             IVVEDIG--VRGTSKEITNMILTHFTRMRKAFEEAPSELDEEGVKMFVVKKMIAGITPEMKAMMAGKKMDADSNTRF
c20470_g1_i3             LVIDGVS--PNLLRTEEEENFIKLGNVMKESLKAVSPDTEESIAKEAIL-KYIEDKFEDQPSPLTL--FDAN----T
c18404_g1_i2             LVVEEMV--WEYTRKDRKTAVL-ILELLKMSLRTIPPQADETIARKAIH-KYLESYVPVTSSTSGFSVFVG-----E
c6245_g2_i1              LVVEDIA--ARAIRQKDGGIILNIIDLLKESLNVIPAGADELTAKKAVV-EYMKSFIPE-GQNMPISSFDMD----F
c19629_g1_i1             LFVEDMV--VEGFNPKAINTVLQVIDLLRKSLNVIPPDADEMAAKMAVY-EFMKSVLPPGSTTGFI--YDAD----T
                         :.:                 .       .  :           ..  :    :

ABH-DCP 11 (S.mimosarum) LPLTCSDGK-CRWKFNMKVLARIANNPLAQLTNS-TGVYNGPALFVYGTESPFKVREVEPEIKQLFPNAKLFPIKGA
c19801_g1_i1             YELTKVSGH-FKWKTDHEFLINKYREGA--FRPPKRGFSNHEILIIRCNNSLRVPDKKFAAVLRFNPNAKLISIHDA
c20557_g1_i1             TNLIEINEK-VKWKANIDVLHKTFQSEICDFSHF-TGCFMDETLFICGGESPYVKEDDHPLIKQIFPKAEFIIVPGA
c18622_g1_i2             LPIRLVNDK-WQWKMNLDLWQKKAENPDDLLNKS-TGLFEGPTLFIYGTASPFDVPGDKENIIKLFPNAQLVPVQGA
c20594_g1_i1             LKLRRDANGRYASVCNMEAIEKSLRNTDSLYSKP-EGKFDGPACFIYGKISPLLVGADEHHIKQFFPNAVFEGIEEV
c20470_g1_i3             LPIRKQDIG-FSWQTNIDVLLDFQLNEDKMLEISNRRAYRQDTLFLCGGKSSFQIKKD-PLIQKLFPRAVMMGLDER
c18404_g1_i2             LPLRKIGDQ-YTWQTNMDPTLNFLRNESSIRNST---VYTGEALFLSGKKSSFDMKND-VSISRYFPRAVKVSFEEA
c6245_g2_i1              LPMKKEGDR-FEWQANFDALEDFLSTEKLVHDVSKLGVYNGDAIFLYGTKSFFRLNKD-VLIEKLFPRSVKVAFEGG
c19629_g1_i1             LALKKDGNA-YAWQANLDVLEDMLRNRP---TQALSGVYDGDALFLYGNKSFFDVGKD-ESISKYFPRAAKFCVEGA
                           :            : .                         ::     *          : .  *.:    .

                                                        ID(%)   FPKM
ABH-DCP 11 (S.mimosarum) ------------------------------  100.0       -
c19801_g1_i1             THLLMFEKQEEFVAAVEDFMS---------   22.3    6.08
c20557_g1_i1             GHWVHSEKPTEFLEALCNFL----------   27.1   11.08
c18622_g1_i2             GHLIHGKYRQ-FEDEIIKFFNKIK------   37.7  235.71
c20594_g1_i1             GHAVHDSKPLEFIDILLKFLQ---------   22.0    1.65
c20470_g1_i3             SHVVRQENPQRYGKIVALFLKGLFPLAAKY   25.2   46.36
c18404_g1_i2             DHYLHYACPDGFLREVIKFVSV--------   22.7  207.84
c6245_g2_i1              THLLHHQFPQRFERIVTDFINKGLLPTSKI   24.3    1.26
c19629_g1_i1             GHLIHQDFPEVFLTEVLKFI----------   32.7    1.84
```

**Fig A12: Sequence alignment of transcripts similar to AB hydrolase containing proteins.** Alignment was performed with MUSCLE, using Abhydrolase domain-containing protein 11 from spider *Stegodyphus mimosarum* (UNIPROT A0A087TEG2), as reference. Signal peptide is highlighted in yellow. The green line marks the common AB hydrolase domain. Percentage of identity (ID%) with the reference proteins was calculated using the tool EMBOSS Stretcher for pairwise sequence alignment. FPKM shows the transcript abundancy.

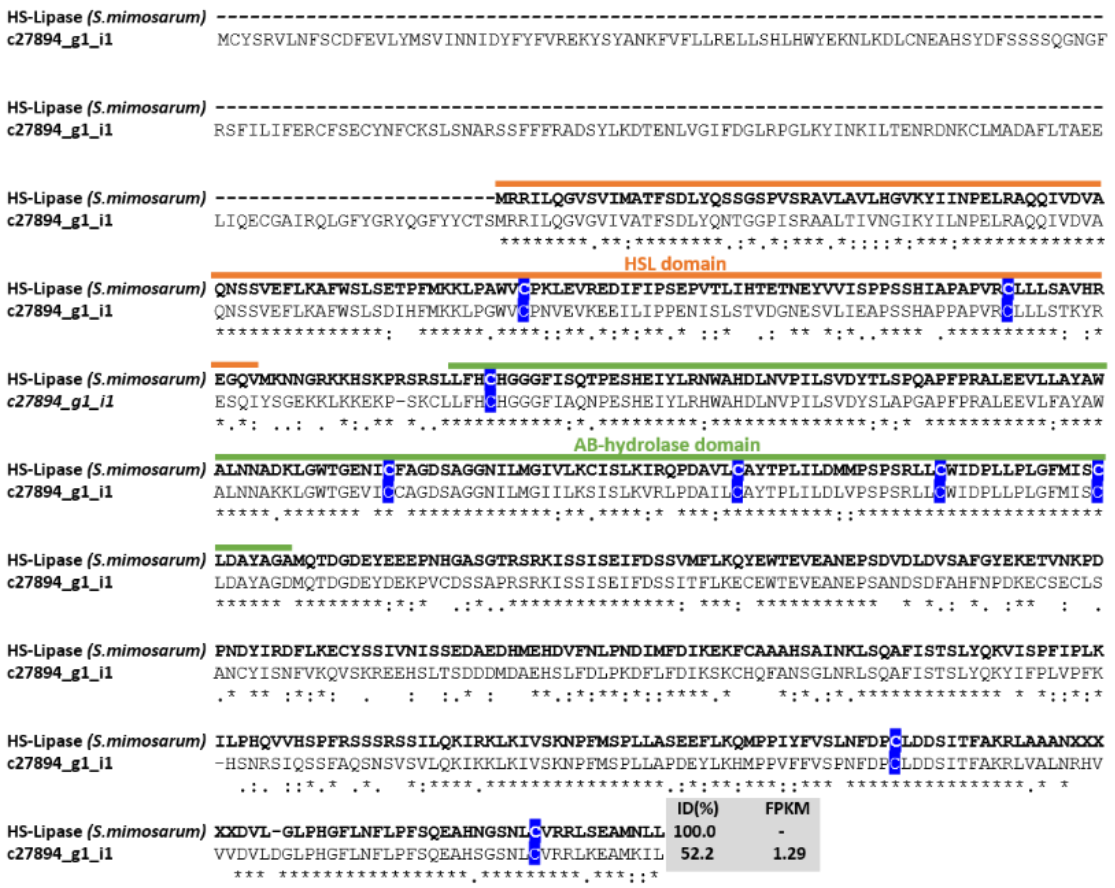


**Fig A13: Sequence alignment of transcripts similar to Hormone-sensitive lipase.** Alignment was performed with MUSCLE, using Hormone-sensitive lipase from spider *Stegodyphus mimosarum* (UNIPROT A0A087TCE8), as reference. Conserved cysteines are highlighted in blue. The green line marks the common AB hydrolase domain and the orange line marks common HSL domain. Percentage of identity (ID%) with the reference proteins was calculated using the tool EMBOSS Stretcher for pairwise sequence alignment. FPKM shows the transcript abundancy.

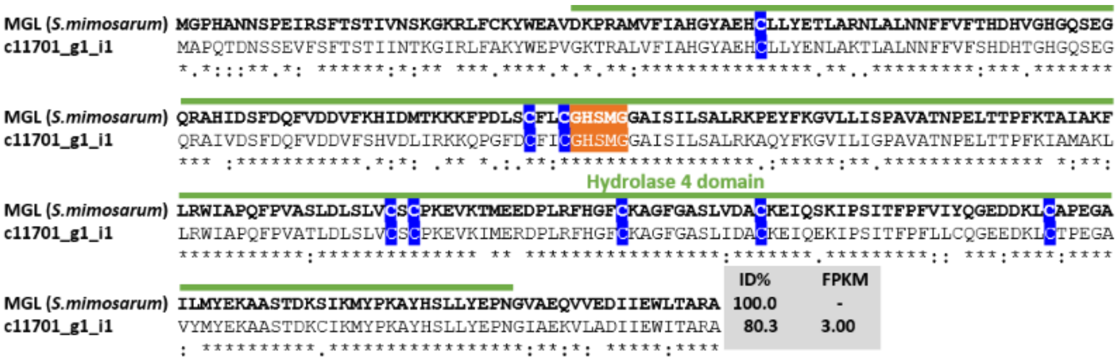


**Fig A14: Sequence alignment of transcripts similar to Monoglyceride lipase.** Alignment was performed with MUSCLE, using Monoglyceride lipase from spider *Stegodyphus mimosarum* (UNIPROT A0A087TCJ1), as reference. Conserved cysteines are highlighted in blue. Residues composing the nucleophilic elbow are highlighted in orange. The green line marks the common hydrolase-4 domain. Percentage of identity (ID%) with the reference proteins was calculated using the tool EMBOSS Stretcher for pairwise sequence alignment. FPKM shows the transcript abundancy.

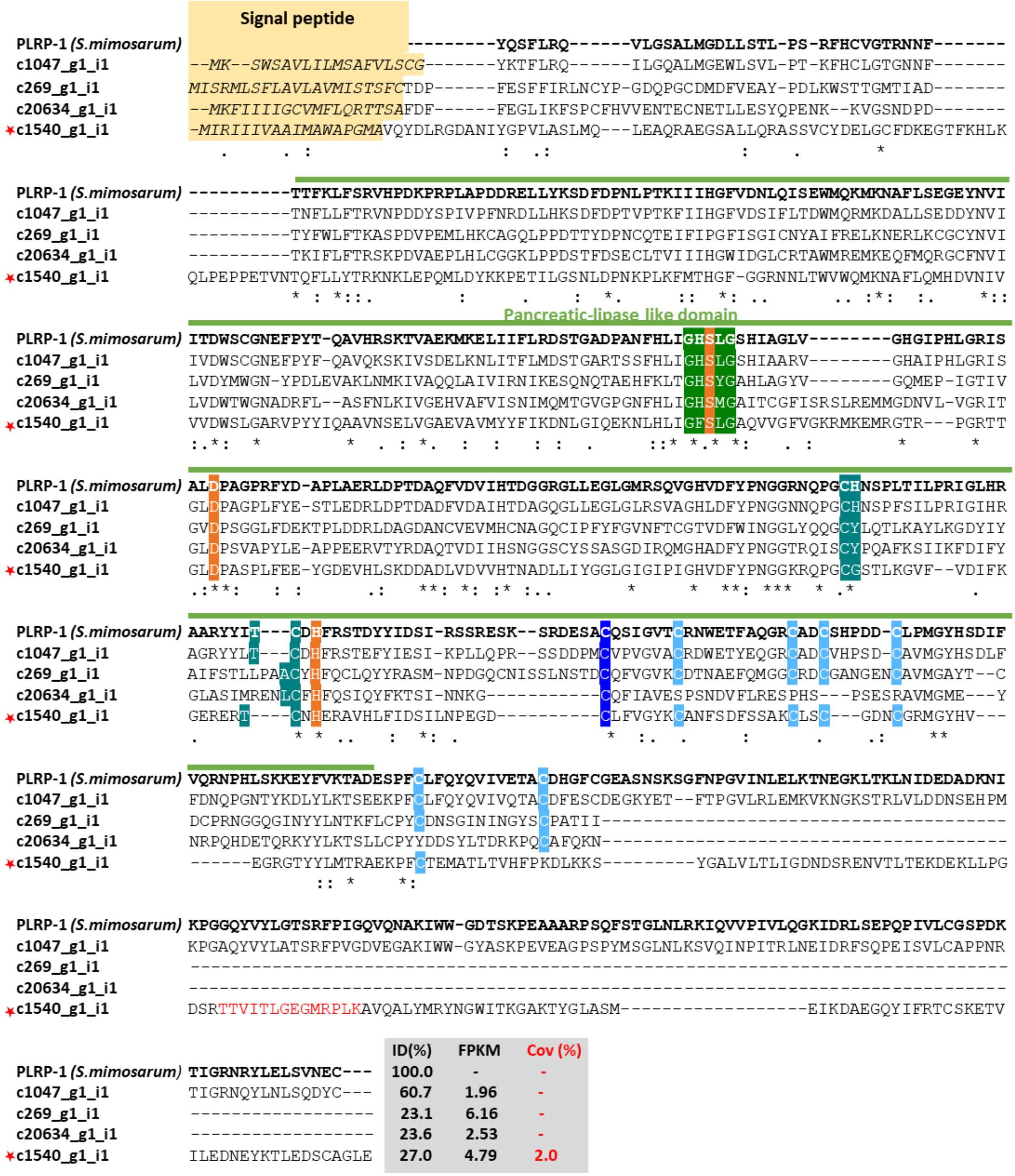


**Fig A15: Sequence alignment of transcripts similar to pancreatic lipase.** Alignment was performed with MUSCLE, using Pancreatic lipase-related protein 1 from spider *Stegodyphus mimosarum* (UNIPROT A0A087T896), as reference. Conserved cysteines are highlighted in blue and semi-conserved ones are in cyan. Residues composing the nucleophilic elbow are highlighted in green and residues composing the active site are in orange. Residues composing the active site lid are highlighted in dark cyan. The green line marks the common pancreatic lipase-like domain. Sequences marked by red stars were confirmed in the proteome and amino acid residues in red correspond to the peptide sequences obtained by MudPIT analysis. Percentage of identity (ID%) with the reference protein was calculated using the tool EMBOSS Stretcher for pairwise sequence alignment. FPKM shows the transcript abundancy. Peptide coverage (Cov %) shows the percentage of the original transcript sequence confirmed by the proteome.

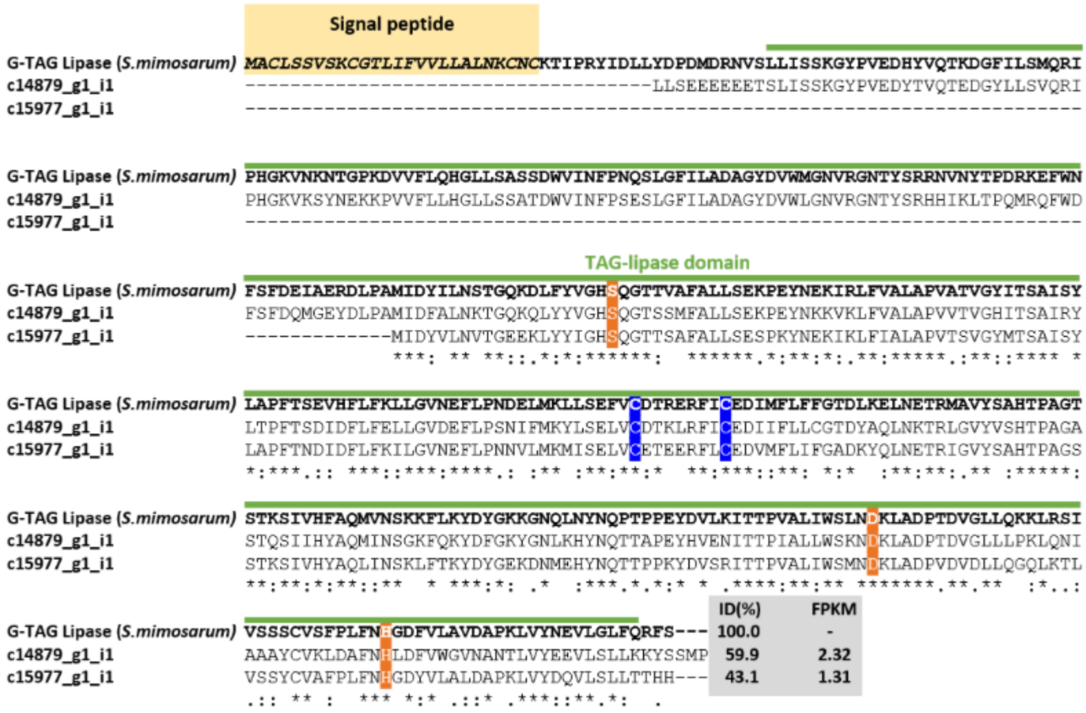


**Fig A16: Sequence alignment of transcripts similar to gastric triacylglycerol lipase.** Alignment was performed with MUSCLE, using Gastric triacylglycerol lipase from spider *Stegodyphus mimosarum* (UNIPROT A0A087UC58), as reference. Conserved cysteines are highlighted in blue. Residues composing the active site are highlighted in orange. The green line marks the common TAG-lipase domain. Percentage of identity (ID%) with the reference proteins was calculated using the tool EMBOSS Stretcher for pairwise sequence alignment. FPKM shows the transcript abundancy.

# METALLOPROTEINASES

```
                      Signal peptide                                       Propeptide
LALP1 (L.intermedia)  MIKYIGVFAFLVG--------------------------GFCHDFETVISNQDPIVDGMRLVE-------------GDMLFD
c14242_g1_i1 ★        --MIWAVLIISLGLASAR-----DTRNVSPQYLDSLRASGRDEEFARLLGV-DVSLVRPNTEERSEGSRYNFVSPLGDILLT
c20028_g1_i1          MSSFVALFSLLLAISSGFSYAENEDSGISPEFLDMLLATKQYERFAEVMEIPDVNLVRPNGERNEYPMFNKALEFGDILPAE
c15061_g1_i1          MPFPWSLVVLAMCHAAFA-----RRPAVTPEYLGLLQLSGQDEELARVLGLEDVSLLRPNEVS-SDGYMFTNVSPLGDILLT
c1763_g1_i1           MSALLGYFLFALCHVAF---------SVEPEYLDMLRATGQDEEFASIMGL-DVSLVRPQEGRTEEELMYTTVSPLGDILLT
c17892_g1_i2 ★        ------------------------------------------------LGV-------------------------------
c39533_g1_i1 ★        MFTAVGFLLLTAG----------SSLATSRVYLG------------------DLPIQNPDLFG-------------GDILGV

LALP1 (L.intermedia)  DGP--LFTERNAVKYDQQ---LWPNGEIVYEISP--GLRQYEQIIREAMRTYEDNT-CIKFRRR-TNEADYVNIHVGDRCYS
c14242_g1_i1 ★        -----EEQERSVIDFNKYGGSKWQGGIIPIHVTD-DFTDAERDFIYRACIDWNERCDKVQIVLC-TYQEDCLYIFSGQGCWS
c20028_g1_i1          KGQPLREESRQAIRFDRYPGSKWPNG-VPFTISD-EFTSSEKRTIEAGIRRWNSNITCVKLRPR-NGEKDYVHVFPGQGCWS
c15061_g1_i1          KEQ--LEQGRQGIDFDRHPESKWPNNEIPYTFAWFGHSSSEKSFIEDCINYLNSRT-NVKFRPRVRGDDDYIRFTNGNGCYA
c1763_g1_i1           PEQ--EEQARQGIRFDKYPGSRWPNARIPYAISNSDFTASQQRTIQEAINEFNQRTEVAKLVLR-NRESDFCYVFSGSGCWS
c17892_g1_i2 ★        -----EVVDRNVIPHSHL---RWTGRTVPYTIDP--AIAGYTSLINSAIQDYHSRT-CVRFTPR-RFEQNYIRIFAGQGCYS
c39533_g1_i1 ★        E----DDEDRNAIVNKIL---LWPGGIVPYEEDPGLKANVFKLTLQGAFDQYKRDT-CIKFVPR-TNEKDYIRLFSGEGCYS
                               *. :  .      * .  :                 :         .:       : :   .  *. *::

LALP1 (L.intermedia)  RVGKSFRGGPQPLSL-GRGCTDFGTILHELGHSVGFDHEHSRADRDEFLIIHKENIKNGSEHNFDKLWENNTRTIGPFDYDS
c14242_g1_i1 ★        YLGRV--GGKQALSLQKNGCLSNGTIQHEIMHACGFAHEHNRIDRDKNVEVLWDNIQSNWHSQYEIVSDEHFVTLGEYDYGS
c20028_g1_i1          YMGRV--GDAQSLSLQRRGCVHIPTILHEFMHAIGFHHEHNRLDRDDSVEVLWGNIEEDWHSQYEKTDSRYFRDHCGYDYYS
c15061_g1_i1          NLGRI--GGAQTVSLQSRGCLNPGTVIHELMHVLGIIHEHNRPDRDNHVIVRWENIPSDWQSQYEKVSEEDFGIQGPYQHIS
c1763_g1_i1           YLGRI--GRNQSISLQRNGCLRKGTIQHEFLHAFGFTHAHNSPQRDNYVRVLWGNIPSDWHSQYEKVSSNDFESQEGYDYYS
c17892_g1_i2 ★        NVGMI--GGQQPVSL-GQGCMFKGTIVHELGHAIGFFHEQNRSDRDQYLTIYWQNIQQGMDTQFFLLKPHENLLLTTFDYNS
c39533_g1_i1 ★        HVGRT--GGQQPVSL-GQGCGWMGTIVHELGHALGFYHEQNRSDRDDWLIIFWDNVKEGMEGQFFKLKPHQNQLLTPFDYSS
                       :*     *  *.:**   .**     *: **: *   *: * :.   :**. : : . *: ..    ::         ::: *

LALP1 (L.intermedia)  IMLYGAYAFSKD-TRKFKTMEPVEPGLPMKSVIQKGKLSYYDIVKVNKLYKCPPVNPYPGGIRPYVNV
c14242_g1_i1 ★        VMHYPMWSPGQPWLDAFKIL---DEGVDRSTLGQDNGLSPGDIAKLNEAYA-----------------
c20028_g1_i1          IMHYRSQAPGSQ-KDAFRVL---KPNINTRRMGNGDGFTQTDIDKIHILYCQGTKPNCTQP-------
c15061_g1_i1          VMHYPYEAPRTR-KPAFEIL---NKTIDSSSLGQRDGPTDIDIEKINTLYP-----------------
c1763_g1_i1           VMHYGMNAPGTG-KPAFEIL---NNSVDKNKIGQRNGPTDTDVKNVDTLYS-----------------
c17892_g1_i2 ★        IMLYGNTAFSKDGRSNTMVA---KTGQRLLETYDKPGLSASDVQRVQKMYGC----------------
c39533_g1_i1 ★        IMLYGSYTFSKD-RKKLKTMVG-KNNEFLQEVISKYRLSKSDIERVNTLYNCKM--------------
                      :*  *    :                .          .     :   *:  .:    *
```

| | ID(%) | FPKM | Cov (%) |
|---|---|---|---|
| LALP1 (L.intermedia) | 100.0 | - | - |
| ★ c14242_g1_i1 | 23.2 | 139.47 | 6.8 |
| c20028_g1_i1 | 27.3 | 88.84 | - |
| c15061_g1_i1 | 26.1 | 16.94 | - |
| c1763_g1_i1 | 23.3 | 29.32 | - |
| ★ c17892_g1_i2 | 31.4 | 14.29 | 26.9 |
| ★ c39533_g1_i1 | 41.0 | 1.98 | 5.3 |

**Fig A17: Sequence alignment of transcripts similar to Astacins.** Alignment was performed with MUSCLE, using Loxosceles astacin like protease 1 (LALP1) from spider *Loxosceles intermedia* (UNIPROT A0FKN6), as reference. Signal peptide is highlighted in yellow. Propeptide is highlighted in light blue. Residues composing the conserved catalytic site are highlighted in orange. Residues highlighted in dark green are part of a structurally important Met-turn. Conserved cysteines are highlighted in blue. Sequences marked by red stars were confirmed in the proteome and amino acid residues in red correspond to the peptide sequences obtained by MudPIT analysis. Percentage of identity (ID%) with the reference protein was calculated using the tool EMBOSS Stretcher for pairwise sequence alignment. FPKM shows the transcript abundancy. Peptide coverage (Cov %) shows the percentage of the original transcript sequence confirmed by the proteome.

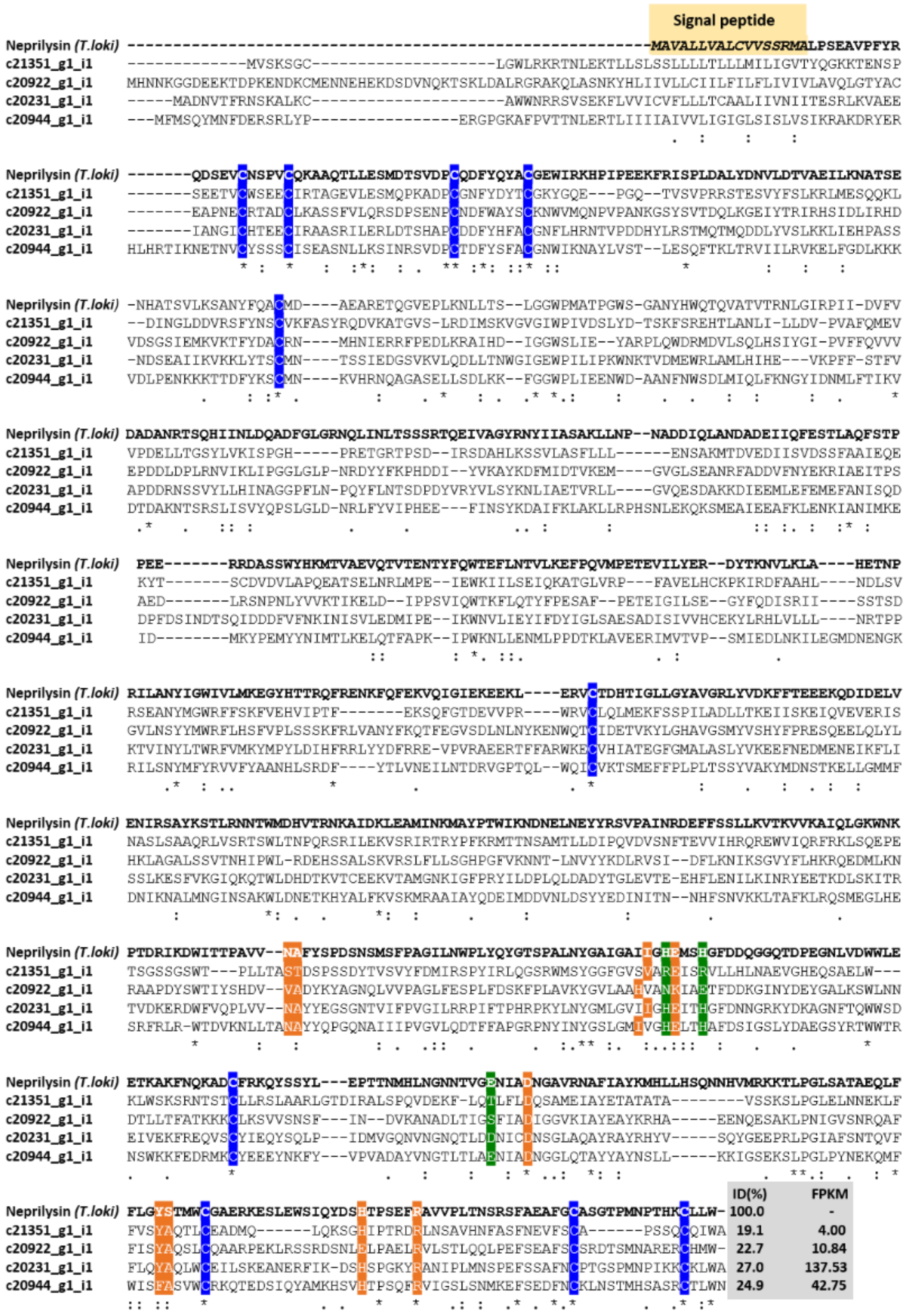


**Fig A18: Sequence alignment of transcripts similar to Neprilysins.** Alignment was performed with MUSCLE, using Neprilysin from spider *Trittame loki* (UNIPROT W4VS99), as reference. Signal peptide is highlighted in yellow. Residues composing the active site are highlighted in orange. Residues highlighted in dark green coordinate with the catalytic zinc ion. Conserved

cysteines are highlighted in blue. Percentage of identity (ID%) with the reference proteins was calculated

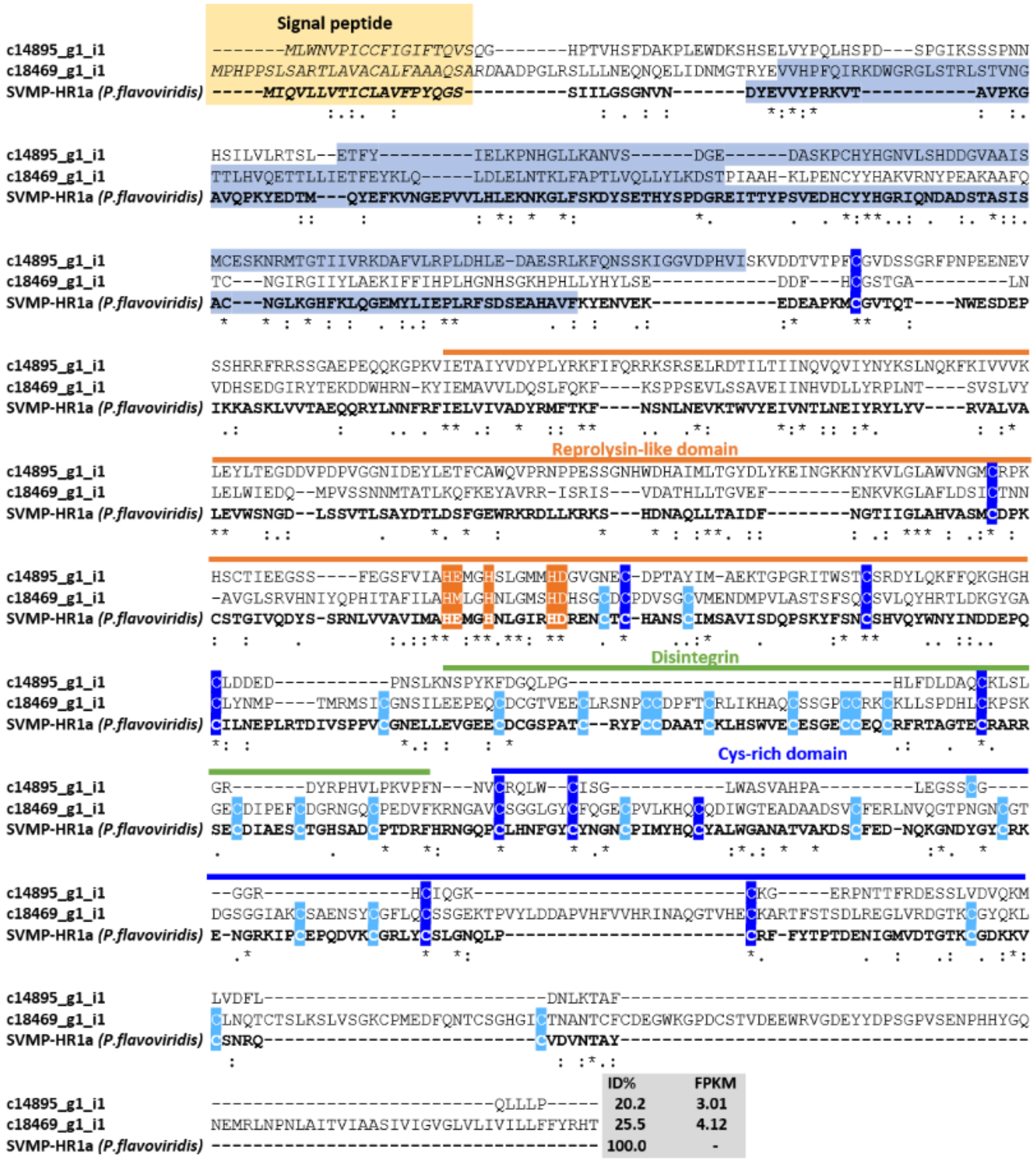


**Fig A19: Sequence alignment of transcripts similar to Reprolysins.** Alignment was performed with MUSCLE, using zinc metalloprotease/disintegrin-like HR1a from snake *Protobothrops flavoviridis* (UNIPROT Q8JIR2), as reference. Signal peptide is highlighted in yellow. Propeptide is highlighted in light blue. Residues that compose zinc binding motif are highlighted in orange, and the conserved reprolysin-like domain is marked by an orange line. The green line marks the disintegrin domain. Conserved cysteines are highlighted in blue, non-conserved ones are in cyan and the cysteine-rich domain is marked also by a blue line. Percentage of identity (ID%) with the reference proteins was calculated using the tool EMBOSS Stretcher for pairwise sequence alignment. FPKM shows the transcript abundancy.

# CATHEPSINS

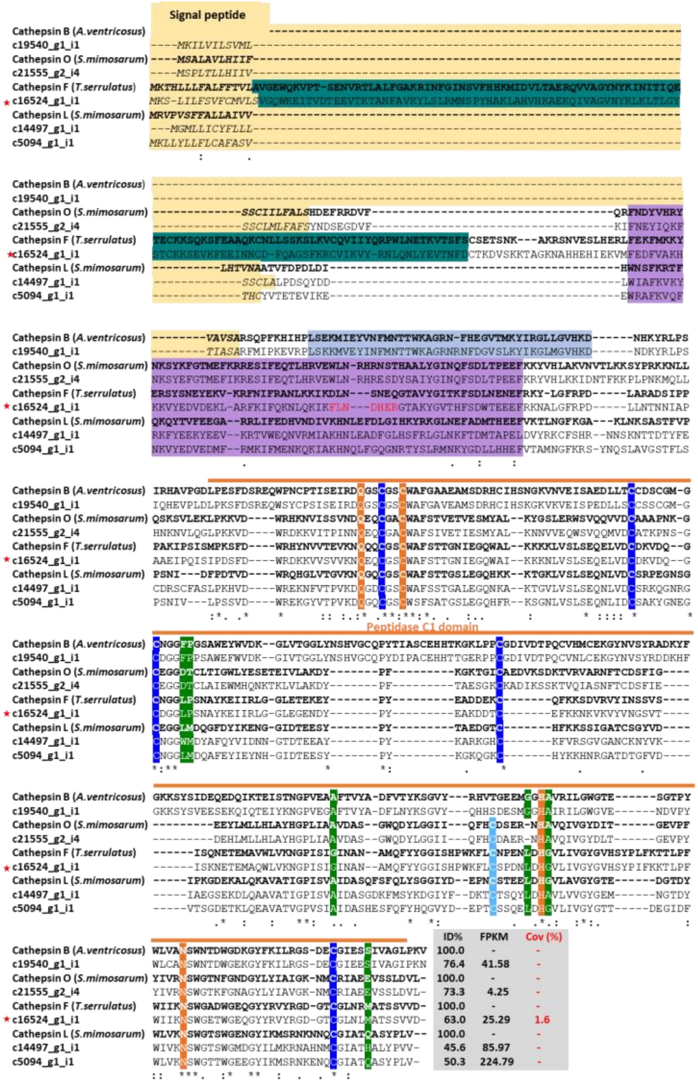


**Fig A20: Sequence alignment of transcripts similar to Cathepsins.** Alignment was performed with MUSCLE, using cathepsin B from spider *Araneus ventricosus* (UNIPROT Q7Z0Z2), cathepsin O and cathepsin L from spider *Stegodyphus mimosarum* (UNIPROT A0A087V0D1 and A0A087UR18) and cathepsin F from scorpion *Tityus serrulatus* (UNIPROT U6JPB2) as references. Signal peptide is highlighted in yellow. Propeptide from peptidase C1 family is highlighted in light blue. Cathepsin propeptide inhibitor domain I29 is highlighted in light purple. Cystatin-like domain is highlighted in light red. Residues composing the conserved catalytic site are highlighted in orange. Residues from the S2 subsite of substrate specificity are highlighted in dark green. Conserved cysteines are highlighted in blue and non-conserved ones are in cyan. Conserved Peptidase C1 domain is marked by an orange line. Sequences marked by red stars were confirmed in the proteome and amino acid residues in red correspond to the peptide sequences obtained by MudPIT analysis. Percentage of identity (ID%) with the reference protein was calculated using the tool EMBOSS Stretcher for pairwise sequence alignment. FPKM shows the transcript abundancy. Peptide coverage (Cov %) shows the percentage of the original transcript sequence confirmed by the proteome.

# SUPEROXIDE DISMUTASE

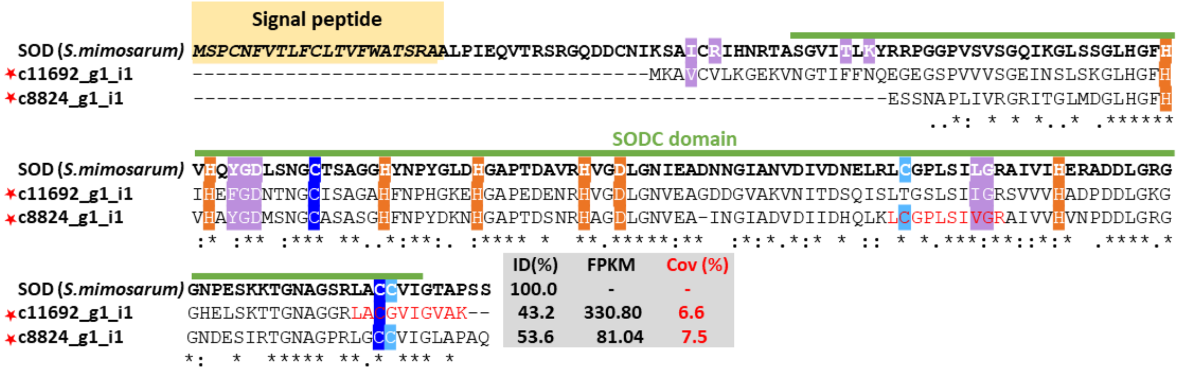


**Fig A21: Sequence alignment of transcripts similar to Superoxide dismutase [Cu-Zn].** Alignment was performed with MUSCLE, using Superoxide dismutase [Cu-Zn] (UNIPROT A0A087V0A2), from *Stegodyphus mimosarum* as reference. Signal peptide is highlighted in yellow. The conserved cysteines are highlighted in blue and semi-conserved ones are in cyan. Active site is highlighted in orange. Residues highlighted in lavender compose the dimeric interface. The green line marks the conserved SODC domain. Sequences marked by red stars were confirmed in the proteome and amino acid residues in red correspond to the peptide sequences obtained by MudPIT analysis. Percentage of identity (ID%) with the reference protein was calculated using the tool EMBOSS Stretcher for pairwise sequence alignment. FPKM shows the transcript abundancy. Peptide coverage (Cov %) shows the percentage of the original transcript sequence confirmed by the proteome.

# CYSTATINS

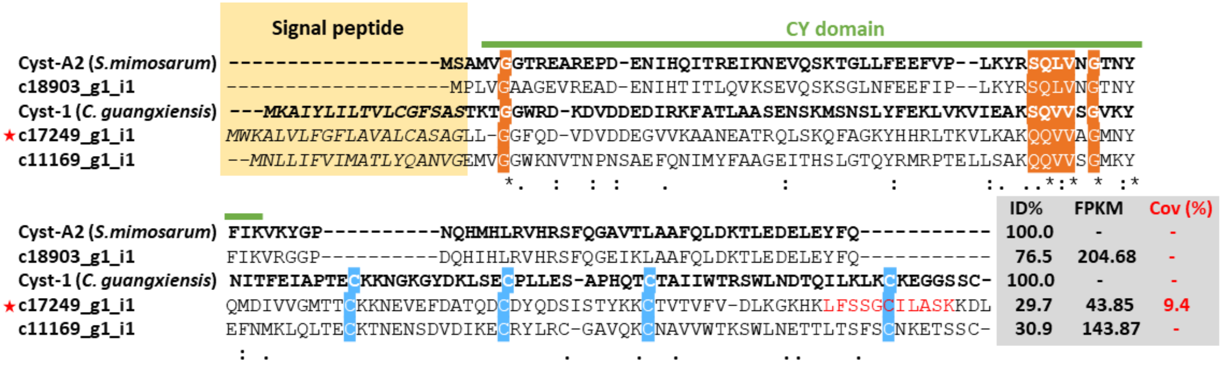


**Fig A22: Sequence alignment of transcripts with Cystatin-like (CY) domain.** Alignment was performed with MUSCLE, using Cystatin-A2 (UNIPROT A0A087UXH2), from *Stegodyphus mimosarum* and Cystatin-1 (UNIPROT B1P1J3), from *Chilobrachys guangxiensis,* as references. Signal peptide is highlighted in yellow. The conserved cysteines are highlighted in cyan. Putative inhibition site is highlighted in orange. The green line marks the conserved CY domain. Sequences marked by red stars were confirmed in the proteome and amino acid residues in red correspond to the peptide sequences obtained by MudPIT analysis. Percentage of identity (ID%) with the reference protein was calculated using the tool EMBOSS Stretcher for pairwise sequence alignment. FPKM shows the transcript abundancy. Peptide coverage (Cov %) shows the percentage of the original transcript sequence confirmed by the proteome.

# ILGFBP-LIKE

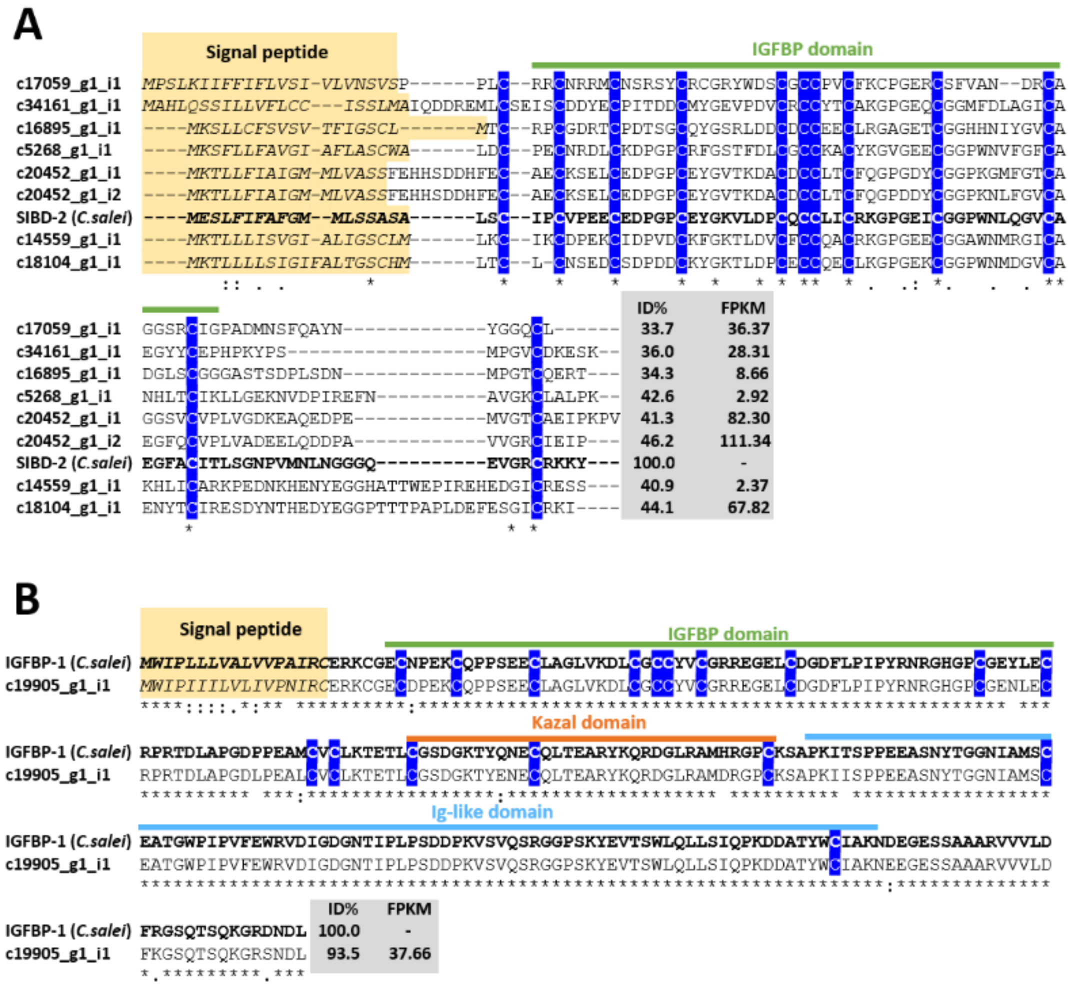


| | ID% | FPKM |
|---|---|---|
| c17059_g1_i1 | 33.7 | 36.37 |
| c34161_g1_i1 | 36.0 | 28.31 |
| c16895_g1_i1 | 34.3 | 8.66 |
| c5268_g1_i1 | 42.6 | 2.92 |
| c20452_g1_i1 | 41.3 | 82.30 |
| c20452_g1_i2 | 46.2 | 111.34 |
| SIBD-2 (C.salei) | 100.0 | - |
| c14559_g1_i1 | 40.9 | 2.37 |
| c18104_g1_i1 | 44.1 | 67.82 |

| | ID% | FPKM |
|---|---|---|
| IGFBP-1 (C.salei) | 100.0 | - |
| c19905_g1_i1 | 93.5 | 37.66 |

**Fig A23: Sequence alignment of transcripts similar to insulin-like growth factor-binding domain protein (IGFBP).** Alignment was performed with MUSCLE, using SIBD-2 from spider *Cupiennius salei* (UNIPROT G4V4G0), as reference for single domain sequences (A) and IGFBP-1, also from spider *Cupiennius salei* (UNIPROT G4V4G1), for longer sequences. Signal peptide is highlighted in yellow. Conserved cysteines are highlighted in blue. Conserved IGFBP domain is marked by an orange line. In the longer sequence, a kazal inhibitor domain is marked by a green line and immunoglobulin- like (Ig-Like) domains is marked by a cyan line. Percentage of identity (ID%) with the reference proteins was calculated using the tool EMBOSS Stretcher for pairwise sequence alignment. FPKM shows the transcript abundancy.

# ACETYLCHOLINESTERASE

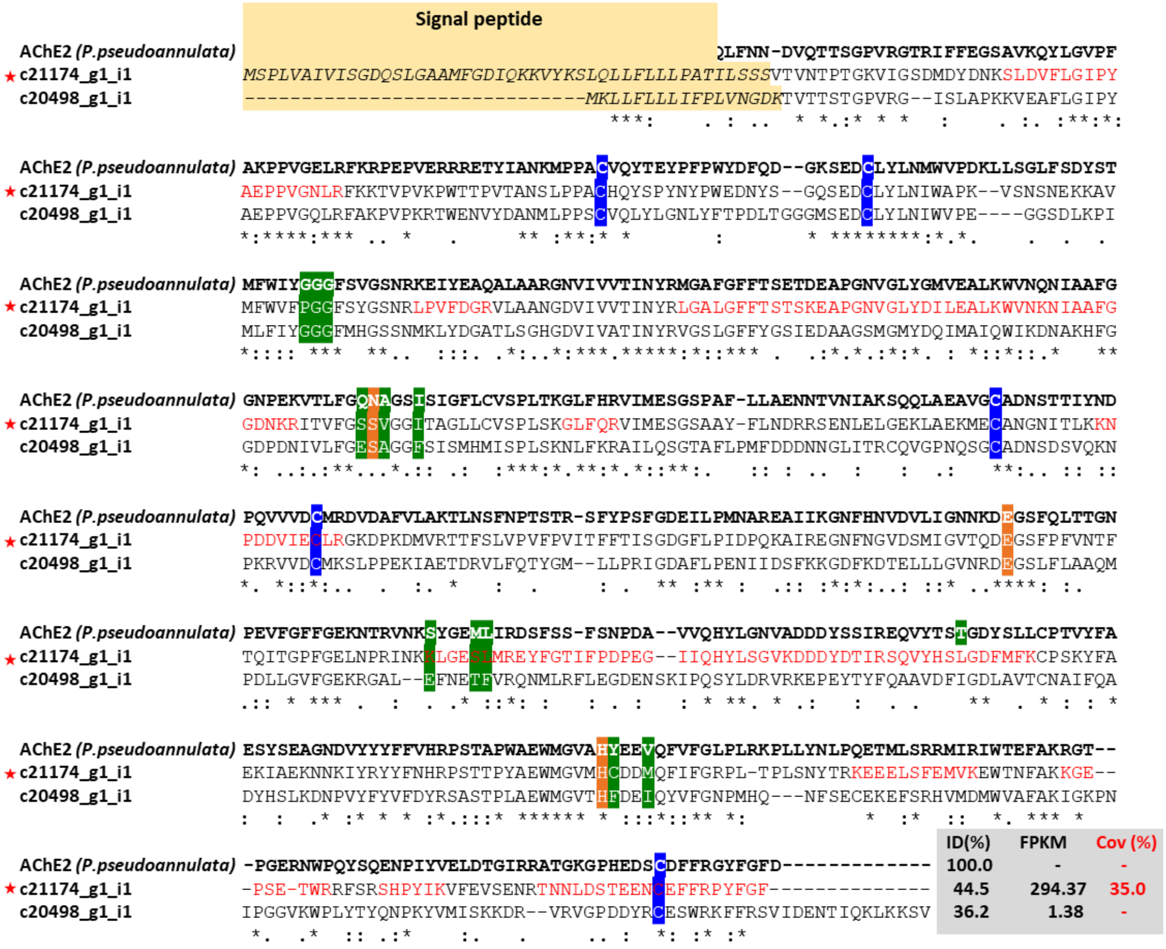


**Fig A24: Sequence alignment of transcripts similar to acetylcholinesterase.** Alignment was performed with MUSCLE, using Acetylcholinesterase type 2 (UNIPROT V5QQR1), from *Pardosa pseudoannulata* spider as reference. Signal peptide is highlighted in yellow. The conserved cysteines are highlighted in blue. Putative active site is highlighted in orange. Residues composing the substrate binding pocket are highlighted in green. Sequences marked by red stars were confirmed in the proteome and amino acid residues in red correspond to the peptide sequences obtained by MudPIT analysis. Percentage of identity (ID%) with the reference protein was calculated using the tool EMBOSS Stretcher for pairwise sequence alignment. FPKM shows the transcript abundance. Peptide coverage (Cov %) shows the percentage of the original transcript sequence confirmed by the proteome.

# KUNITZ-TYPE INHIBITORS

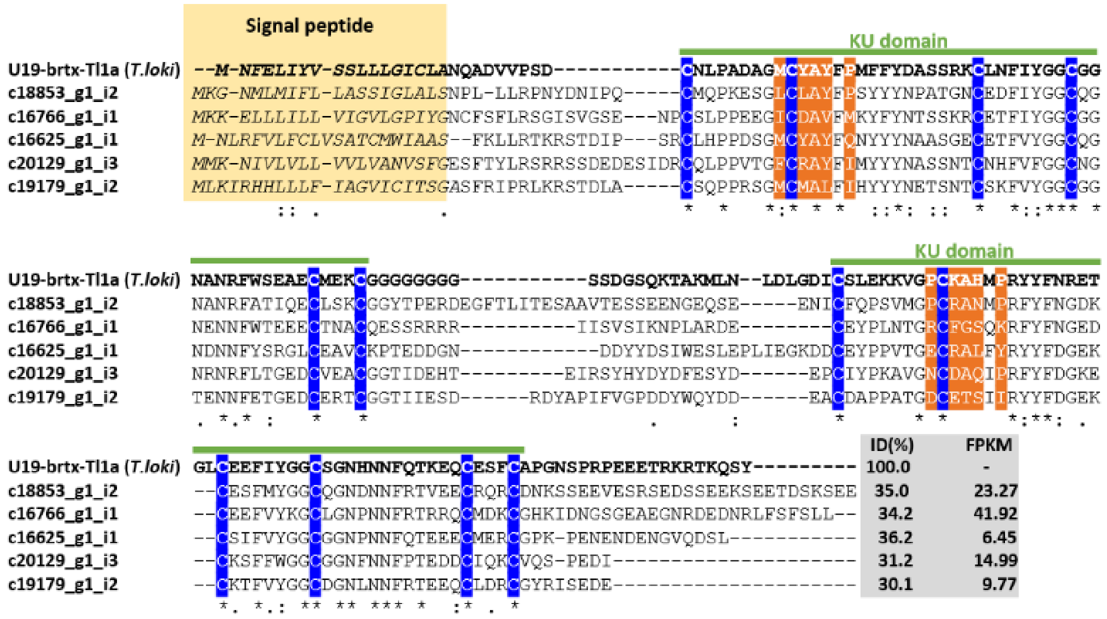


**Fig A25: Sequence alignment of transcripts similar to Kunitz-type inhibitors.** Alignment was performed with MUSCLE, using Kunitz-type U19-barytoxin-Tl1a (UNIPROT W4VSH9), from *Trittame loki* as reference. Signal peptide is highlighted in yellow. The conserved cysteines are highlighted in blue. Putative trypsin binding loop is highlighted in orange. The green line marks the conserved KU domain. Percentage of identity (ID%) with the reference proteins was calculated using the tool EMBOSS Stretcher for pairwise sequence alignment. FPKM shows the transcript abundancy.

# SERPINs

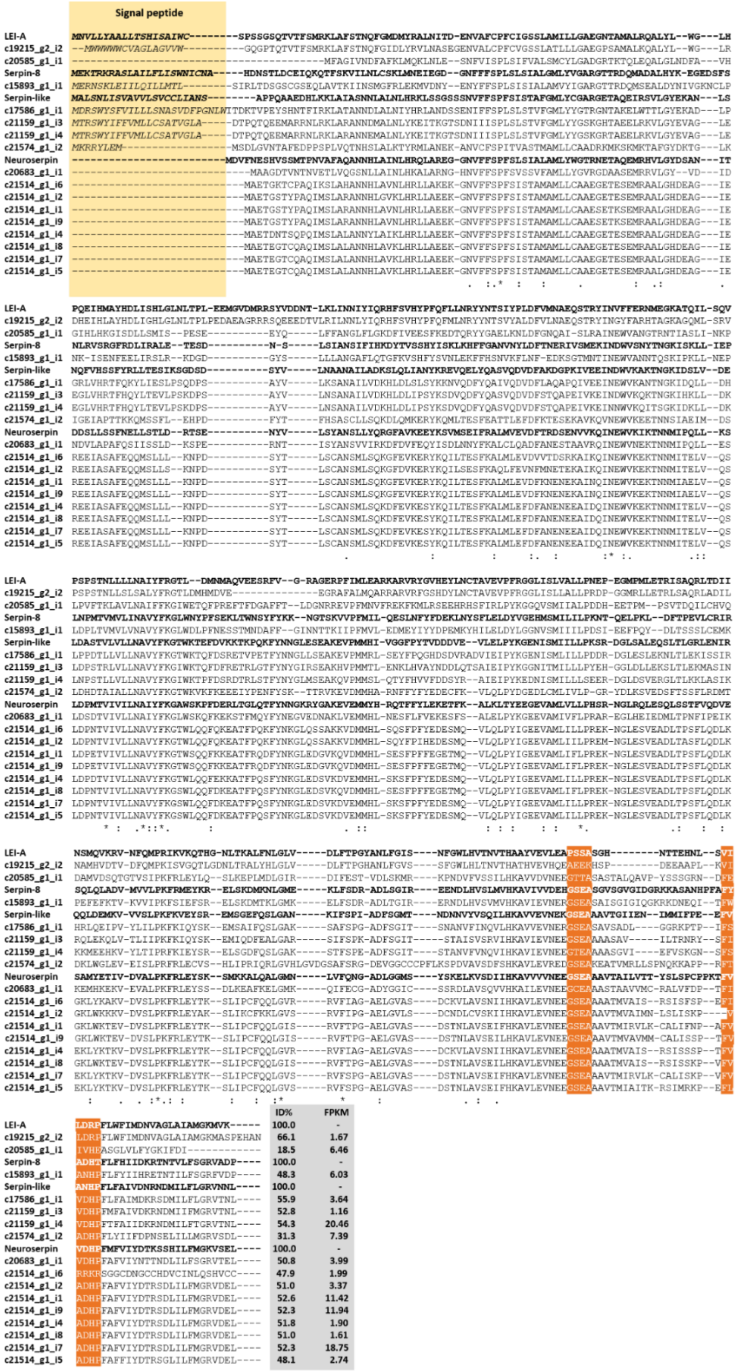


**Fig A26: Sequence alignment of transcripts similar to SERPINs.** Alignment was performed with MUSCLE, using leukocyte elastase inhibitor A (UNIPROT A0A087UU21), serpin B8 (UNIPROT A0A087TC37), putative serpin-like protein (UNIPROT A0A087SZK7) and neuroserpin (UNIPROT A0A087SZS9), all from spider *Stegodyphus mimosarum*, as references. Signal peptide is highlighted in yellow. The reactive center loop (RCL) is highlighted in orange. Percentage of identity (ID%) with the reference proteins was calculated using the tool EMBOSS Stretcher for pairwise sequence alignment. FPKM shows the transcript abundancy.

# AMINOPEPTIDASES

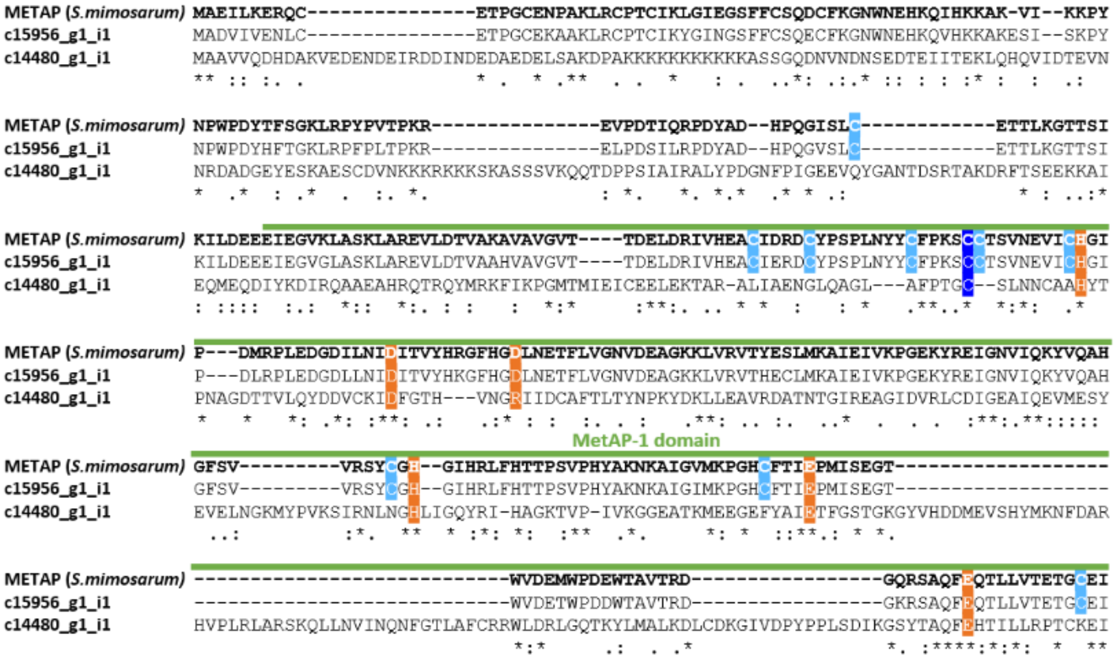


| | ID(%) | FPKM |
|---|---|---|
| METAP (S.mimosarum) | 100.0 | - |
| c15956_g1_i1 | 86.2 | 16.16 |
| c14480_g1_i1 | 16.9 | 8.82 |

**Fig A27: Sequence alignment of transcripts similar to Methionine aminopeptidase.** Alignment was performed with MUSCLE, using Methionine aminopeptidase from spider *Stegodyphus mimosarum* (UNIPROT A0A087T485), as reference. Residues from the active site are highlighted in orange. The conserved cysteines are highlighted in blue and semi-conserved ones are in cyan. Conserved MetAP domain is marked by a green line. Percentage of identity (ID%) with the reference proteins was calculated using the tool EMBOSS Stretcher for pairwise sequence alignment. FPKM shows the transcript abundancy.

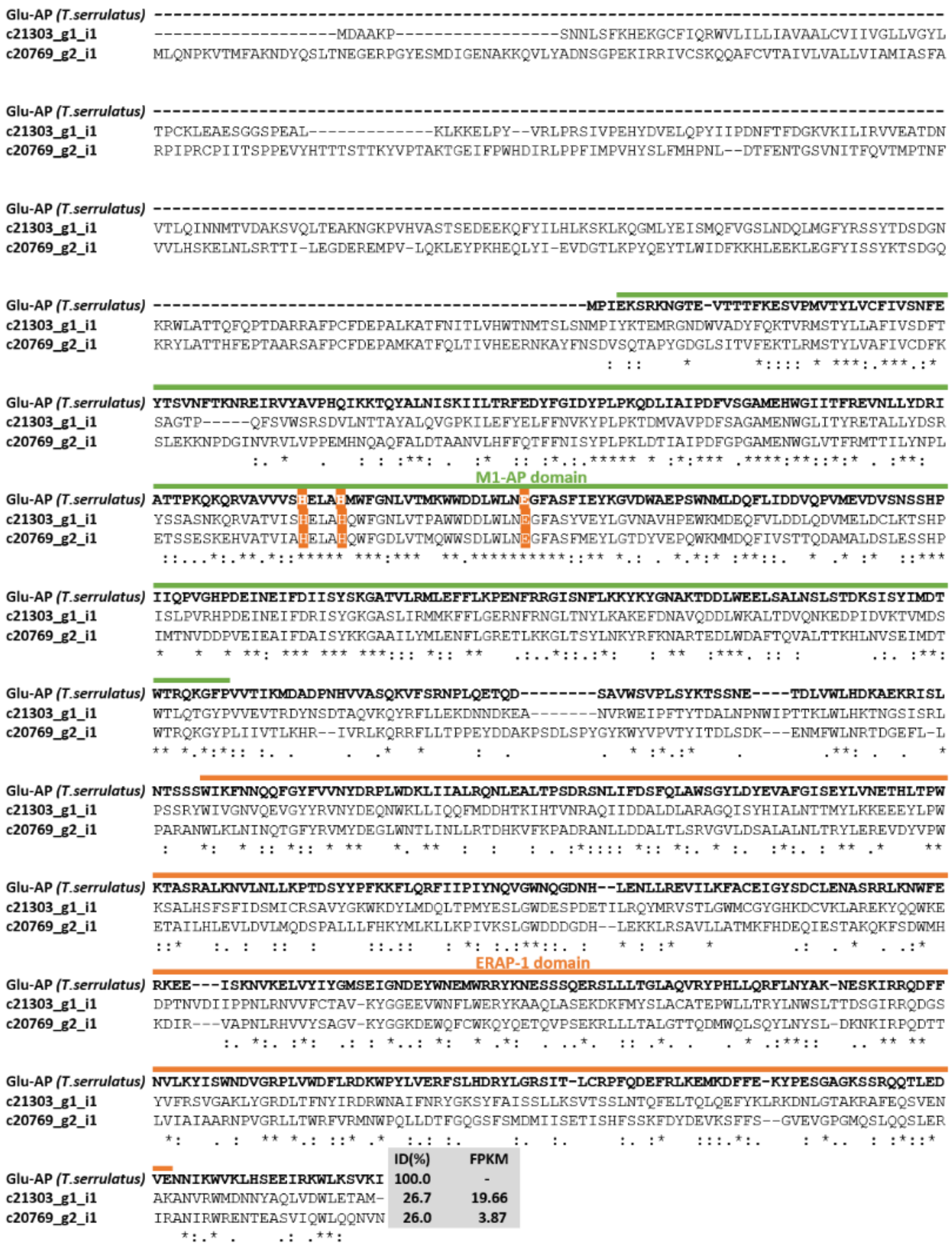


**Fig A28: Sequence alignment of transcripts similar to Glutamyl aminopeptidase.** Alignment was performed with MUSCLE, using Glutamyl aminopeptidase from scorpion *Tityus serrulatus* (UNIPROT A0A1S5QN53), as reference. Residues from the active site are highlighted in orange. Conserved M1AP domain is marked by a green line and ERAP-1 domain is marked by an orange line. Percentage of identity (ID%) with the reference proteins was calculated using the tool EMBOSS Stretcher for pairwise sequence alignment. FPKM shows the transcript abundancy.

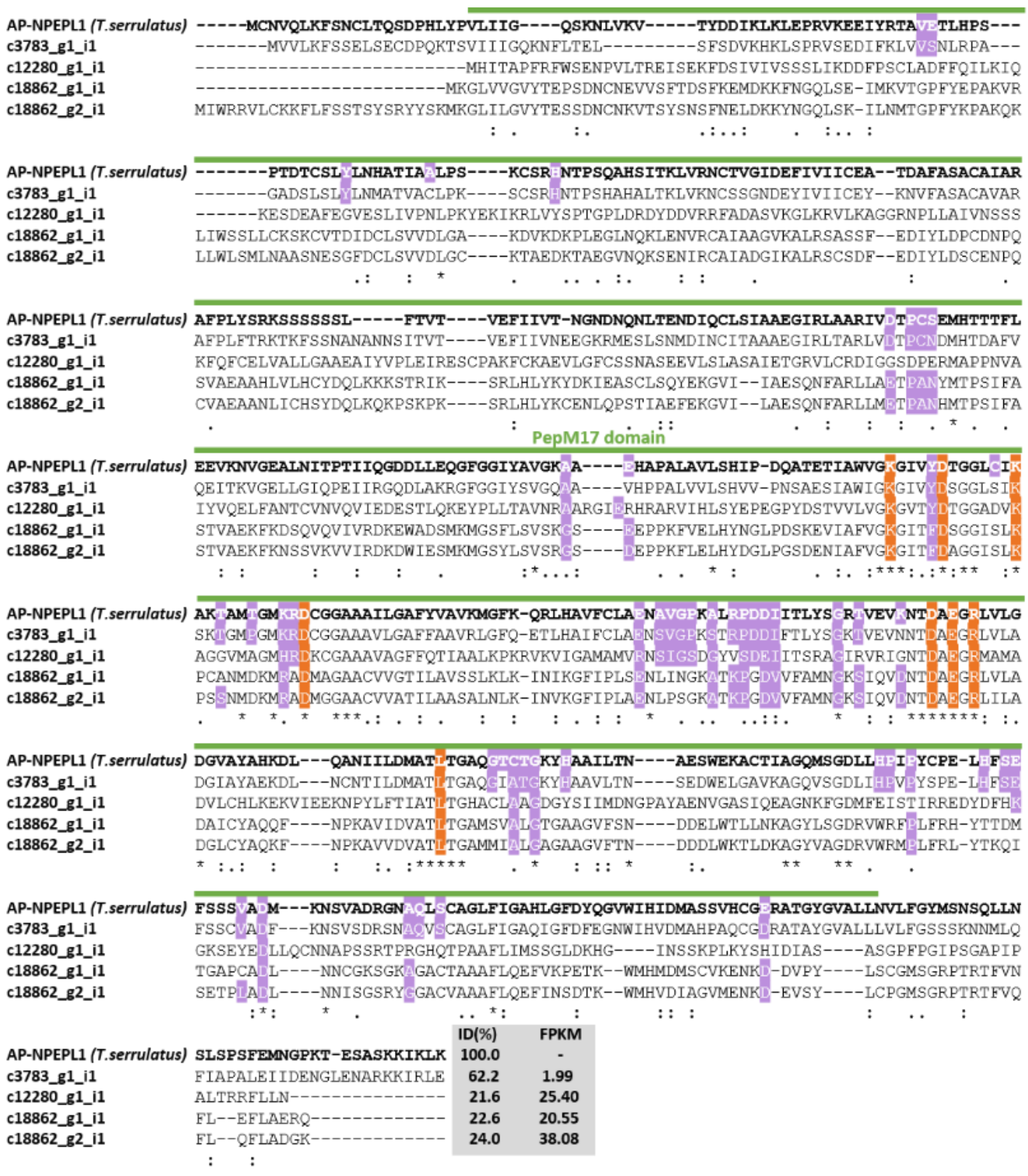


**Fig A29: Sequence alignment of transcripts similar to cytosolic aminopeptidases.** Alignment was performed with MUSCLE, using Aminopeptidase NPEPL1 from scorpion *Tityus serrulatus* (UNIPROT A0A1S5QN31), as reference. Residues from the active site are highlighted in orange and residues composing the trimer interface are in lavender. Conserved PepM17 domain is marked by a green line. Percentage of identity (ID%) with the reference proteins was calculated using the tool EMBOSS Stretcher for pairwise sequence alignment. FPKM shows the transcript abundancy.

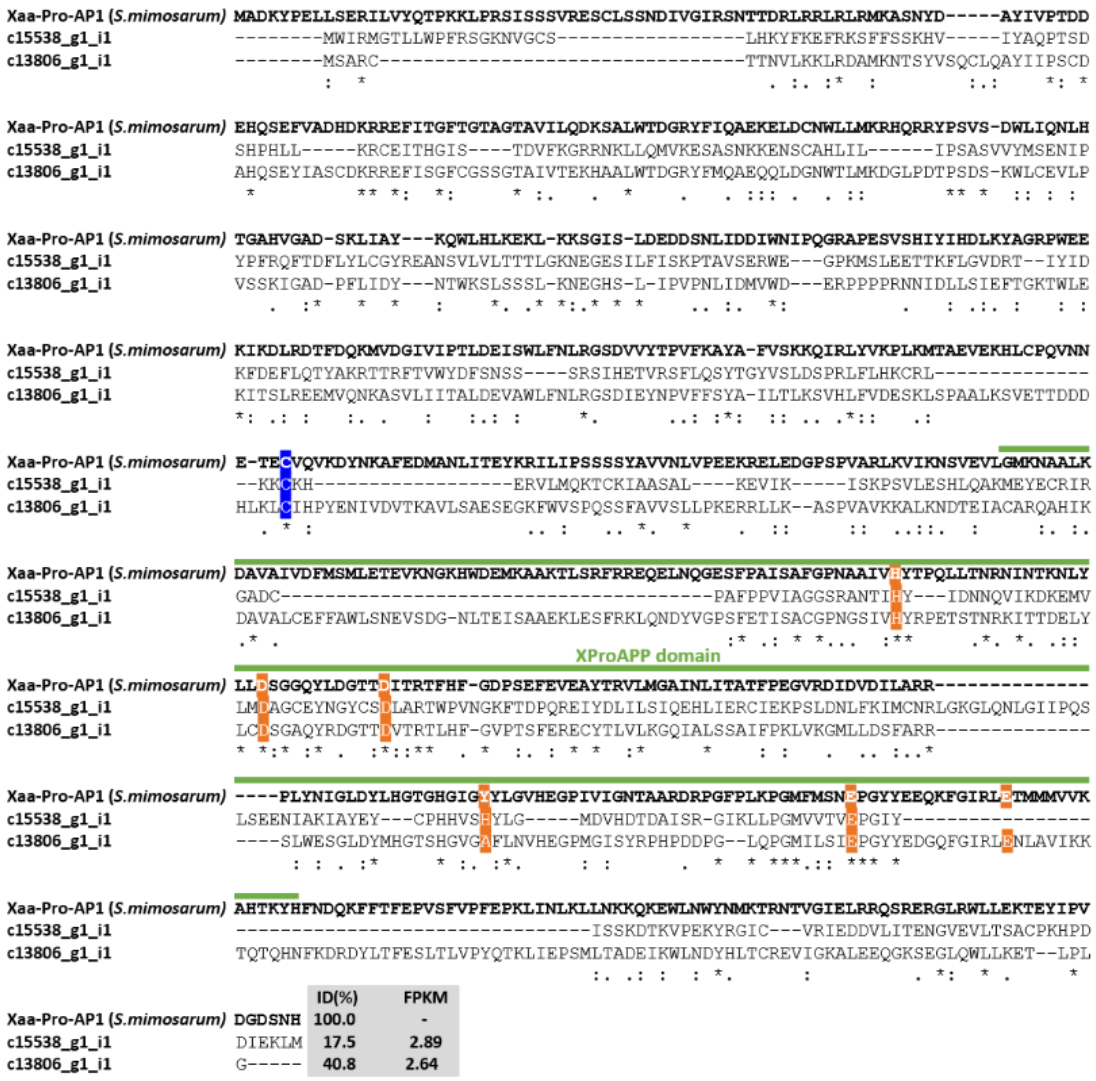


**Fig A30: Sequence alignment of transcripts similar to Xaa-Pro aminopeptidases.** Alignment was performed with MUSCLE, using Xaa-Pro aminopeptidase 1 from spider *Stegodyphus mimosarum* (UNIPROT A0A087SVH2), as reference. Conserved cysteines are highlighted in blue. Residues form the active site are highlighted in orange. Conserved XProAPP domain is marked by a green line. Percentage of identity (ID%) with the reference proteins was calculated using the tool EMBOSS Stretcher for pairwise sequence alignment. FPKM shows the transcript abundancy.

# PHOSPHOLIPASES

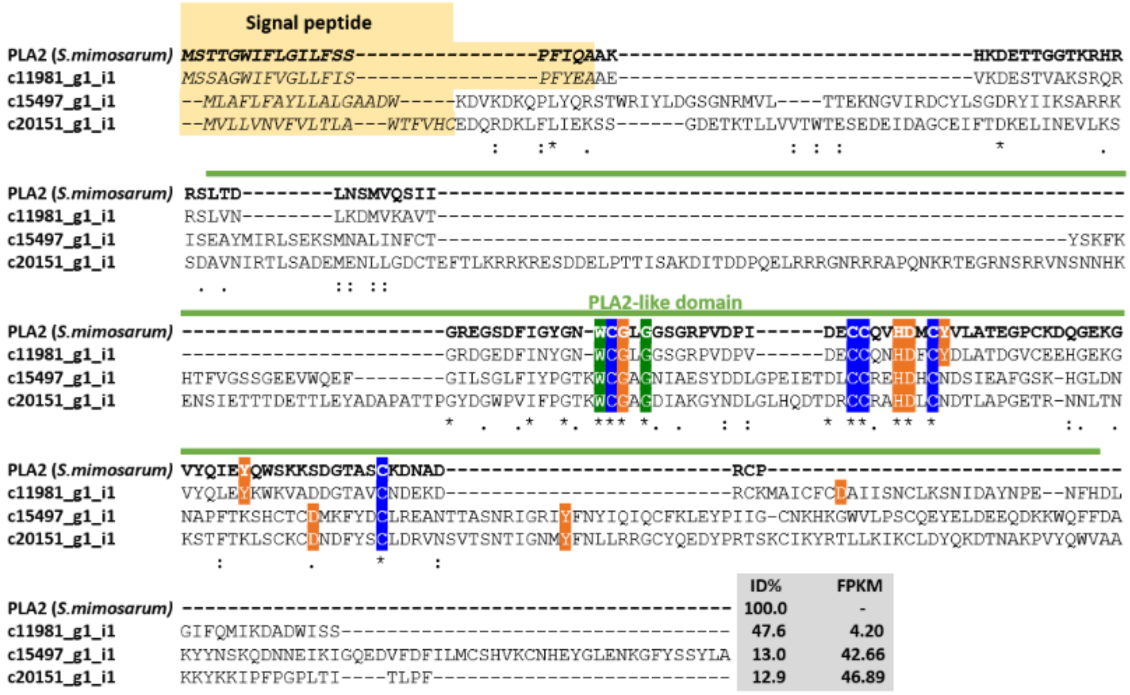


**Fig A31: Sequence alignment of transcripts similar to secretory PLA2.** Alignment was performed with MUSCLE, using PLA2 from spider *Stegodyphus mimosarum* (UNIPROT A0A087TZ71), as reference. Signal peptide is highlighted in yellow. Conserved cysteines are highlighted in blue. Residues form the active site are highlighted in orange. Primary metal binding site residues are highlighted in green. Conserved PLA2-like domain is marked by a green line. Percentage of identity (ID%) with the reference proteins was calculated using the tool EMBOSS Stretcher for pairwise sequence alignment. FPKM shows the transcript abundancy.

```
                              Signal peptide
PLB (C.adamanteus)        MIRFGNPSSSDKRRQRCRSWYWGGLLLLWAVAETRA---DIHYATVYWLEAEKSFQIKDVLDKNGDAYGYYNDAIQST
PLB-Like2 (S.mimosarum)   ------------------------------------------------------------------------------
c18240_g1_i1              --------------------MISYLLLFSCLTSVALSSAIKNASVTYDRQTKKFSLHDSFVSNAVAFANFQDEIFQT

PLB (C.adamanteus)        GWGILEIKAGYGNQPISNEILMYAAGFLEGYLTASHMSDHFAN-LFPLMIKNVIIEQKVKDFIQKQDEWTRQQIKNNK
PLB-Like2 (S.mimosarum)   ------------------------------------------------------------------------------
c18240_g1_i1              GWSYLEVKT---NEQYPDPIQAYAAGIVEGYLTRDLLNKHWYNTVSDYCNGEVLYCQRLAKFLQNNLDFINENIKVRR

PLB (C.adamanteus)        -DDPFWRNAGYVIAQLDGLYMGNVEWAKRQKRTPLTDFEISFLNAIGDLLDLIPALHSELRKSDFRSMPDVSRIYQWD
PLB-Like2 (S.mimosarum)   -------------------------------------------MGDLEDLEVVLKKNNLKKVLGS-----------
c18240_g1_i1              THDVYWHQIALTLEQLKGLEDGSKNITDGQPSTDIDVMGLLLINIMGDIEDLEVVLHKN-QKRVFGS-----------
                                                                     :**: **  .*:.:  *  : *

PLB (C.adamanteus)        MGHCSALIKVLPGYENIYFAHSSWFTYAATLRIYKHLDFRIT------DPQTKTGRASFSSYPGLFGSLDDFYILGSG
PLB-Like2 (S.mimosarum)   -GSCSGLVKVLPNNENLYVAQDTWSGYNSMLRILKKYIFSFHTAMNEGSPVIPGHTSTFSSQPGLIFSQDDFYLISSG
c18240_g1_i1              -GSCSALVKVLPNFKDLYFSQDTWSSYNTMLRILKKYSLKLHITMDNDASLIPGHTCTFSSQPGMIYSGDDFYLISSG
                           * **.*:****. :::*.::.:*  * : *** *:  : :        .      .:*** **:: * ****::.**

PLB (C.adamanteus)        LIMLQTTNSVFNLSLLKKVVPE-SLFAWERVRIANMMADSGKTWAETFEKQNSGTYNNQYMILDTKKIKLQRSLEDGT
PLB-Like2 (S.mimosarum)   LVAIETTIGNSNTSLYDFVTPEGTILEWQRNIIANRLAKDGRQWANLFEIMNSGTYNNQWMIVDYKKFMPGKPLQDGL
c18240_g1_i1              LATMETTIGNGNASLWQYVKAEGTILEWQRNIVANRLAKNGDQWTKLFGIMNSGTYNNQWMVLDYKKFKPGQPLQDGL
                          *  ::** .  * ** . * .* ::: *:*  :** :*..*  *:: *   ********:*::* **:    ..*:**

PLB (C.adamanteus)        LYIIEQVPKLVKYSDQTKVLRN-GYWPSYNIPFDKEIYNMSGYGEYVQRHGLEFSYEMAPRAKIFRRDQGKVTDMESM
PLB-Like2 (S.mimosarum)   LWVLEQLPGYTHSEDVTKLLRDQGYFPSYNVAYFPDVFNLSGAQINAQKYGDWFTYDRCPRALIFRRDQGKVSDVPSM
c18240_g1_i1              LWVLEQLPGYLHSEDKTDVLRNQSYWPSYNTAYFKDIFNLSGGQLNVDKYGDWFTYEKNPRALIFKRDHSKVSDIDGM
                          *:::**:*   : .* *.:**: .*:**** .:  :::*:**    .:.:*  *:*:  *** **.**:.**:*: .*

PLB (C.adamanteus)        KFIMRYNNYKEDPYAKHNPC------NTICCRQDLDRRT---PVPA------GCYDSKVADISMAAKFTAYAINGPPV
PLB-Like2 (S.mimosarum)   IKLMRYNDYTNDPLSRCNCTPPYSAENAISARCDLNPINGTYPFAALGHRQHGGTDMKLTTFDLFQNMEFVAFGGP--
c18240_g1_i1              IKLMRYNDYTNDPLSRCNCTPPYSAENAISARCDLNPSNGTYPFAALGHRQHGATDMKLTNYEMFKRFEFVSFGGPTY
                            :****:*.:** :. *         *:*..* **:  .   *..*      *  * *::  .:  .:   ::.**

PLB (C.adamanteus)        EKGLPVFSWVHFN---KTKHQGLPESYNFDFVTMKPVL
PLB-Like2 (S.mimosarum)   --------------------------------------
c18240_g1_i1              DP-LPPFQWSKSDFGKTVKHEGHPDLWKFKPIIHKWL-
```

| | ID% | FPKM |
|---|---|---|
| PLB (C.adamanteus) | 34.1 | - |
| PLB-Like2 (S.mimosarum) | 47.0 | - |
| c18240_g1_i1 | - | 8.84 |

**Fig A32: Sequence alignment of transcripts similar to Phospholipase B.** Alignment was performed with MUSCLE, using PLB from snake *Crotalus adamanteus* (UNIPROT F8S101) and PLB-Like2 from spider *Stegodyphus mimosarum* (UNIPROT A0A087TYC3) as references. Signal peptide is marked in yellow. Conserved cysteines are highlighted in blue. Percentage of identity (ID%) with the reference proteins was calculated using the tool EMBOSS Stretcher for pairwise sequence alignment. FPKM shows the transcript abundance.

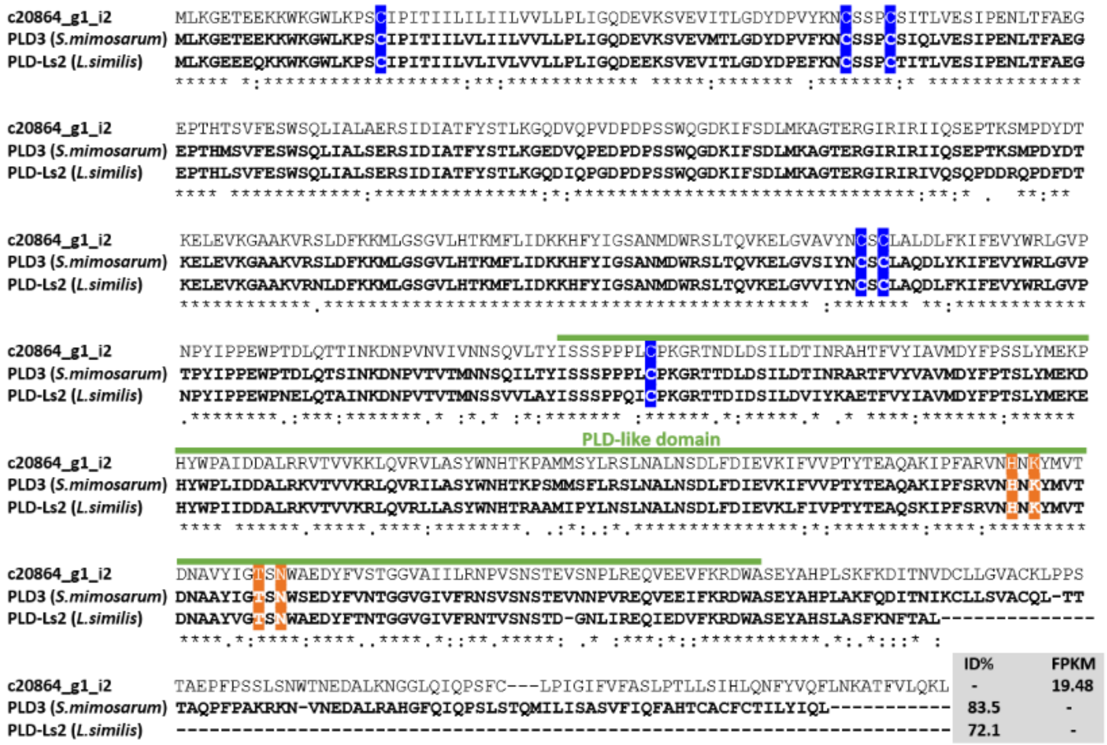

**Fig A33: Sequence alignment of transcripts similar to Phospholipase D3.** Alignment was performed with MUSCLE, using PLD3 from spider *Stegodyphus mimosarum* (UNIPROT A0A087TLF9) and PLD-Ls2 from spider *Loxosceles similis* (GENBANK ANY30958.1) as references. Signal peptide was not identified. Conserved cysteines are highlighted in blue. Residues form the active site are highlighted in orange. Conserved PLD-like domain is marked by an orange line. Percentage of identity (ID%) with the reference proteins was calculated using the tool EMBOSS Stretcher for pairwise sequence alignment. FPKM shows the transcript abundancy.

# DOPAMINE BETAL-HYDROXILASE

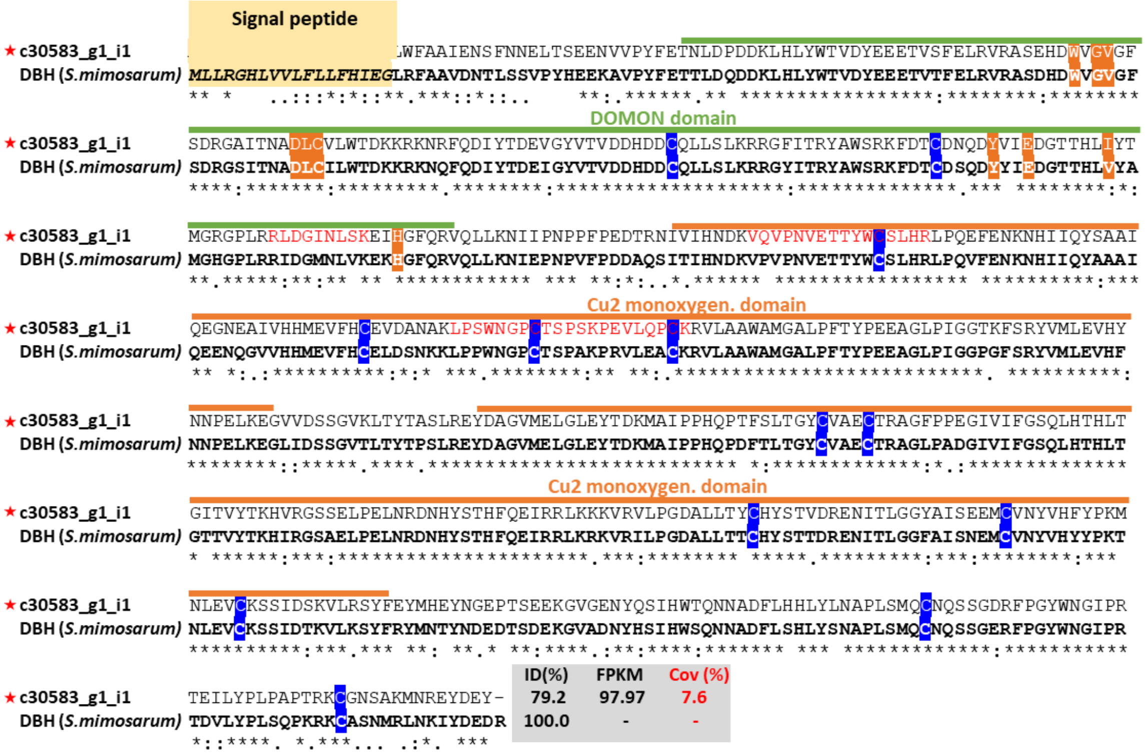


**Fig A34: Sequence alignment of transcripts similar to dopamine-beta hydroxilase.** Alignment was performed with MUSCLE, using Dopamine beta-hydroxylase from spider *Stegodyphus mimosarum* (UNIPROT A0A087TQF1) as reference. Signal peptide is highlighted in yellow. Conserved cysteines are highlighted in blue. Residues responsible for substrate binding are highlighted in orange. Conserved Cu2-monoxygenase domain is marked by an orange line and DOMON domain is marked by a green line. Sequences marked by red stars were confirmed in the proteome and amino acid residues in red correspond to the peptide sequences obtained by MudPIT analysis. Percentage of identity (ID%) with the reference protein was calculated using the tool EMBOSS Stretcher for pairwise sequence alignment. FPKM shows the transcript abundancy. Peptide coverage (Cov %) shows the percentage of the original transcript sequence confirmed by the proteome.

# 5’ NUCLEOTIDASE

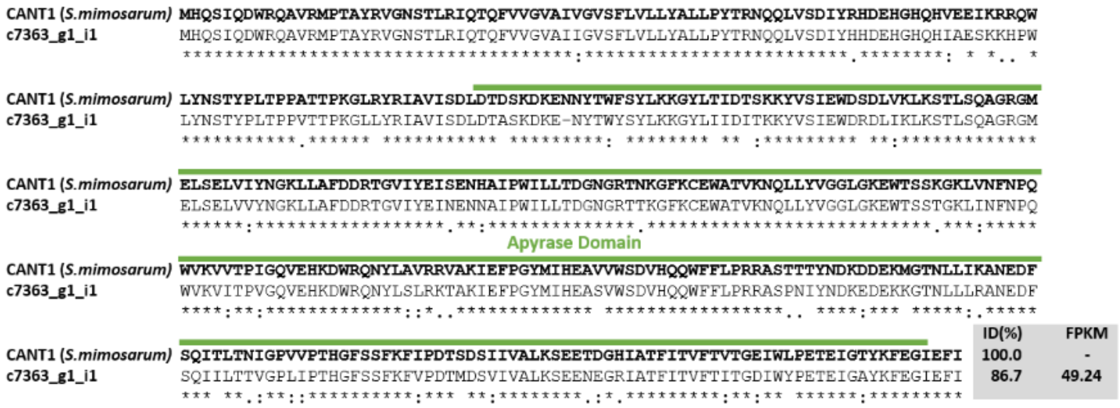


**Fig A35: Sequence alignment of transcripts similar to Soluble calcium-activated nucleotidase.** Alignment was performed with MUSCLE, using Soluble calcium-activated nucleotidase 1 from spider *Stegodyphus mimosarum* (UNIPROT A0A087TKB4) as reference. Common apyrase domain is marked by a green line. Percentage of identity (ID%) with the reference proteins was calculated using the tool EMBOSS Stretcher for pairwise sequence alignment. FPKM shows the transcript abundancy.

```
CP-5'NUC (S.mimosarum) -------------------------------------------------------------------------------
c16609_g1_i1           METQLSVEHDESRSESTCSTTQSRKYYRATQHRVFVNRSLHLEKIQFFGFDMDYTLAQYNSPEYEALQFNLIVDRLVFI

CP-5'NUC (S.mimosarum) ------------------------MYGNLLKVDAYGNILVCYHGFRFLKTSEIYNLYPNKFIQHHESRIYILNTLFNLP
c16609_g1_i1           GYPAQIKDFEYDPAFPIRGLWFDKLYGNLLKVDAYGNILVCCHGFKFLKTSEIYNLYPNKFIQHDESRIYMLSTLFNLP
                                               :**************** ***.****************** *****:*.******

CP-5'NUC (S.mimosarum) EAYLLACLIDYFTNSTEYIPGKNGIKNGNIFMSYKSIFEDVRDAIDWVHMRGTLKEETVNNLSKYVARDERLPMLFNRM
c16609_g1_i1           EAYLLACLVDFFSNSAVYIPGKNGIKNGNIFMSYKSIFQDVRDAIDWVHMRGSLKEETVNNLSKYVNRDERLPMLFNRM
                       ********:*:*:**: *********************:*************:************* ************

CP-5'NUC (S.mimosarum) HEVGKKTFLLTNSEFDYTTKIMSYLFDFPSEKKRDWKEYFDYILVDARKPVFFSGGTTLRQVDTETGALRIGIHVGPLT
c16609_g1_i1           HEVGKKAFLLTNSDYAYTAKIMSYLFDFPSEQKKDWREYFDYILVDARKPVFFSGGTTLRQVDTETGALKIGIHVGPLA
                       ******:******:: **:************:*.**.********************************.********:

CP-5'NUC (S.mimosarum) PGQVYSGGNSDVFTSLIGASGKDVLYIGDHIYGDILKSKKTRGWRTFLVVPELQREVHVWTTKCHLFNKLQDLDVQLGD
c16609_g1_i1           PGQVYSGGNSDVFTALIGANGKDVLYIGDHIYGDILKSKKTRGWRTFLIVPELQREVHVWTTKCQLFHRLQDLDVQLGD
                       **************:****.****************************:***************:**:.**********

CP-5'NUC (S.mimosarum) TYKEMDSSCKDTPDISQLRTAIREVSHELDMSYGILGSTFRSGSRQTFFANQICHYADLYACTFLNLMYYPFSYMFRAP
c16609_g1_i1           TYREMDSSCKDAPDISQLRSAIREVSHELDMSYGILGSTFRSGSRQTFFANQICHYADLYACTFLNLMYYPFSYMFRAP
                       **.********:*******:***********************************************************

CP-5'NUC (S.mimosarum) PMLMPHESTVEHEEKSFFSNGEVDELEEE-AQEPIRRRSRLENSESSVPHLYAETPNVVTHHHDTDDDEDTDKSAEN
c16609_g1_i1           PMLMPHESTVEHEEKSFISNGEVEELEEDIIQEPLRRRSRLENSESSVPHLYAETPNVVTHHHDTDDDEDTDKSAEN
                       *****************:*****:****:  ***:******************************************
```

| | ID(%) | FPKM |
|---|---|---|
| CP-5'NUC (*S.mimosarum*) | 100.0 | - |
| c16609_g1_i1 | 74.4 | 5.03 |

**Fig A36: Sequence alignment of transcripts similar to Cytosolic purine 5'-nucleotidase.** Alignment was performed with MUSCLE, using Cytosolic purine 5'-nucleotidase from spider *Stegodyphus mimosarum* (UNIPROT A0A087TJU6) as reference. Conserved cysteines are highlighted in blue. Residues composing the HAD hydrolases signature motif are highlighted in orange. Percentage of identity (ID%) with the reference proteins was calculated using the tool EMBOSS Stretcher for pairwise sequence alignment. FPKM shows the transcript abundancy.

# SPHINGOMIELINASE

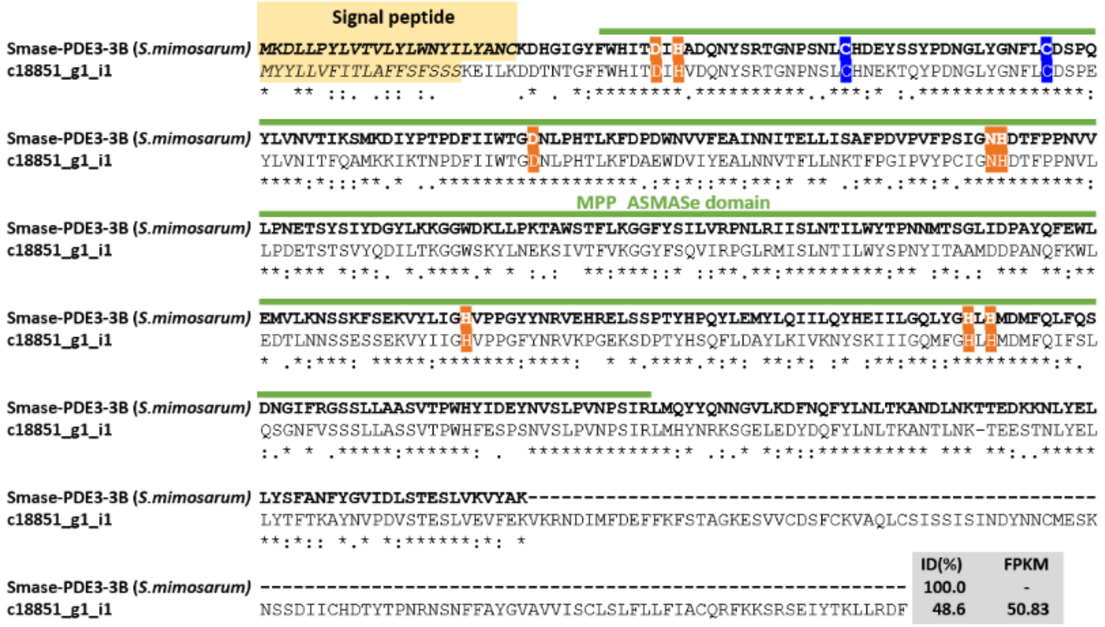


**Fig A37: Sequence alignment of transcripts similar to sphingomielinases.** Alignment was performed with MUSCLE, using acid sphingomyelinase-like phosphodiesterase 3b, from spider *Stegodyphus mimosarum* (UNIPROT A0A087TI01) as references. Signal peptide is highlighted in yellow. Conserved cysteines are highlighted in blue. Residues composing the active site are highlighted in orange. The green line marks the MPP a-Smase domain. Percentage of identity (ID%) with the reference proteins was calculated using the tool EMBOSS Stretcher for pairwise sequence alignment. FPKM shows the transcript abundancy.

# WAPRINS

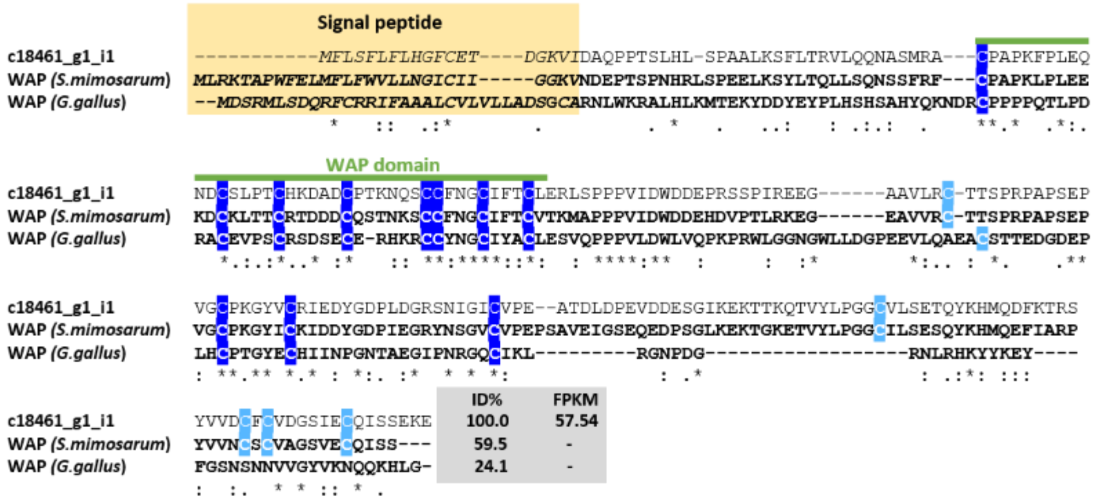


**Fig A38: Sequence alignment of transcripts similar to whey acidic protein-type four-disulfide core domains protein (WAP).** Alignment was performed with MUSCLE, using WAP, from *Gallus gallus* (UNIPROT Q8JG33) and from spider *Stegodyphus mimosarum* (UNIPROT A0A087U8D6) as references. Signal peptide is highlighted in yellow. Conserved cysteines are highlighted in blue and non-conserved ones are in cyan. The green line marks the WAP domain. Percentage of identity (ID%) with the reference proteins was calculated using the tool EMBOSS Stretcher for pairwise sequence alignment. FPKM shows the transcript abundancy.

# CHITINASES

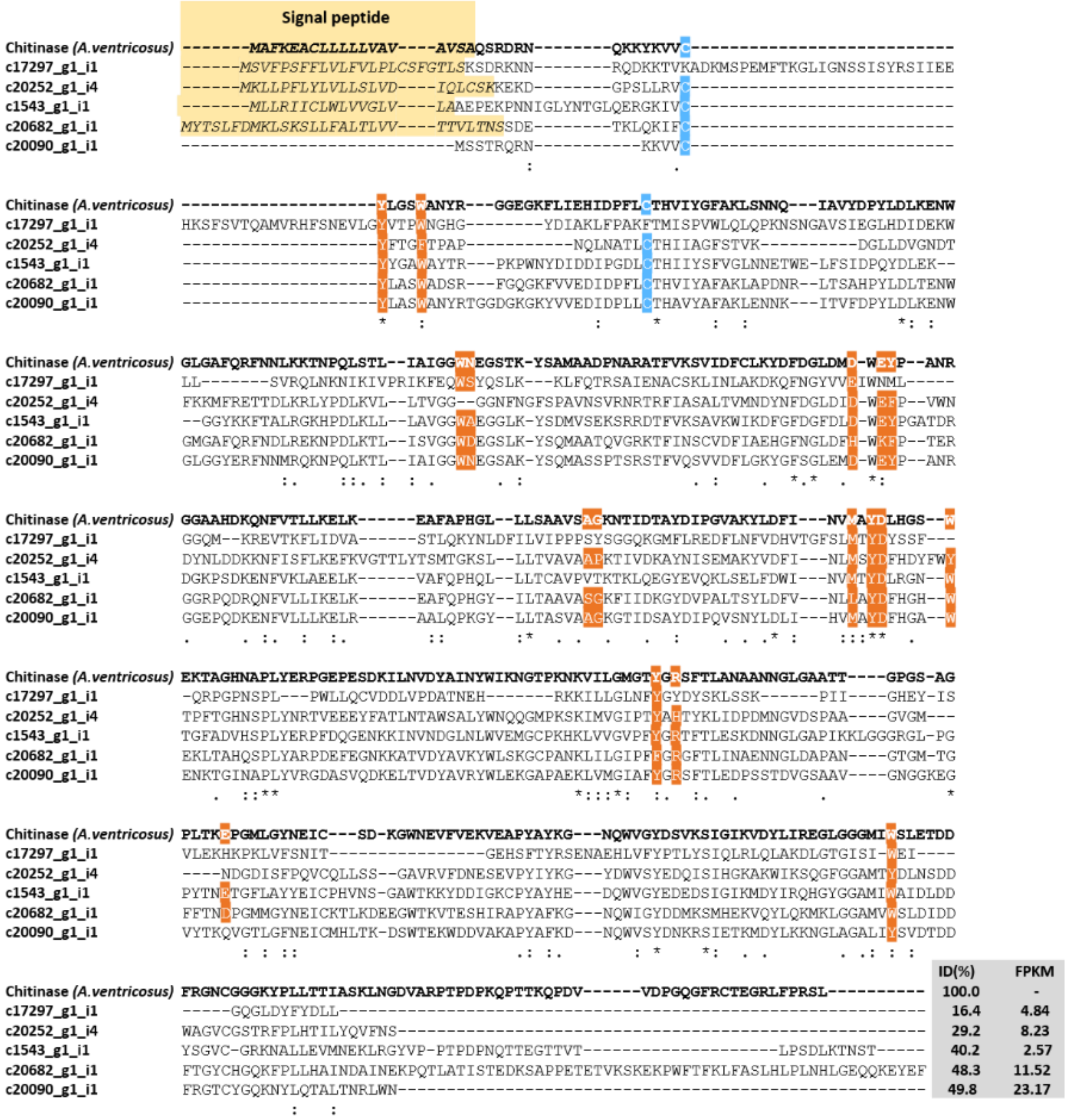


**Fig A39: Sequence alignment of transcripts similar to chitinases.** Alignment was performed with MUSCLE, using Chitinase, from spider *Araneus ventricosus* (UNIPROT Q8ISH5) as references. Signal peptide is highlighted in yellow. Conserved cysteines are highlighted cyan. Residues from the active site are highlighted in orange. Percentage of identity (ID%) with the reference proteins was calculated using the tool EMBOSS Stretcher for pairwise sequence alignment. FPKM shows the transcript abundancy.

# ANGIOTENSIN-CONVERTING ENZYME

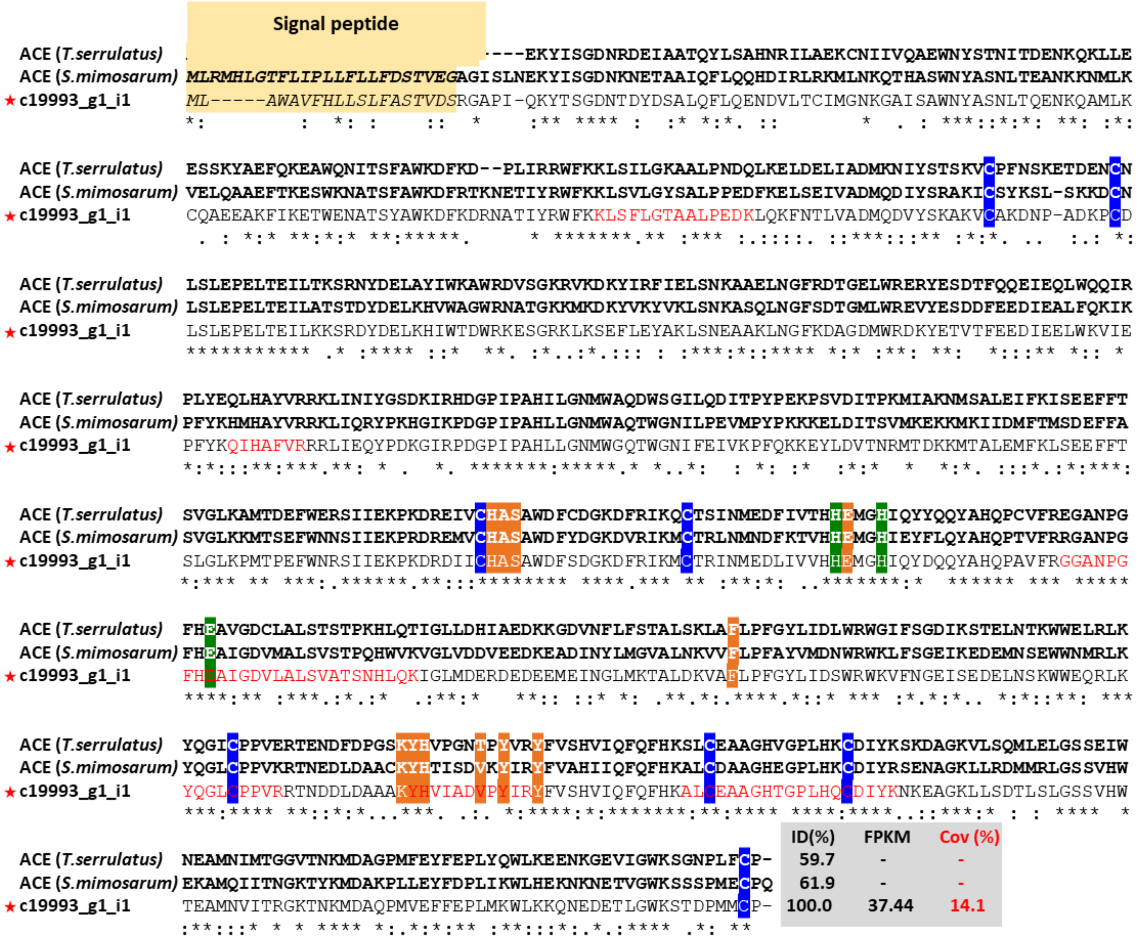


**Fig A40: Sequence alignment of transcripts similar to angiotensin-converting enzymes.** Alignment was performed with MUSCLE, using angiotensin-converting enzyme from spider *Stegodyphus mimosarum* (UNIPROT A0A087UCM7) and from scorpion *Tityus serrulatus* (UNIPROT A0A1S5QMZ5) as references. Signal peptide is highlighted in yellow. Residues that compose the active site are highlighted in orange and residues that bind to zinc are in green. Conserved cysteines are highlighted in blue. Sequences marked by red stars were confirmed in the proteome and amino acid residues in red correspond to the peptide sequences obtained by MudPIT analysis. Percentage of identity (ID%) with the reference protein was calculated using the tool EMBOSS Stretcher for pairwise sequence alignment. FPKM shows the transcript abundancy. Peptide coverage (Cov %) shows the percentage of the original transcript sequence confirmed by the proteome.

# CATALASE

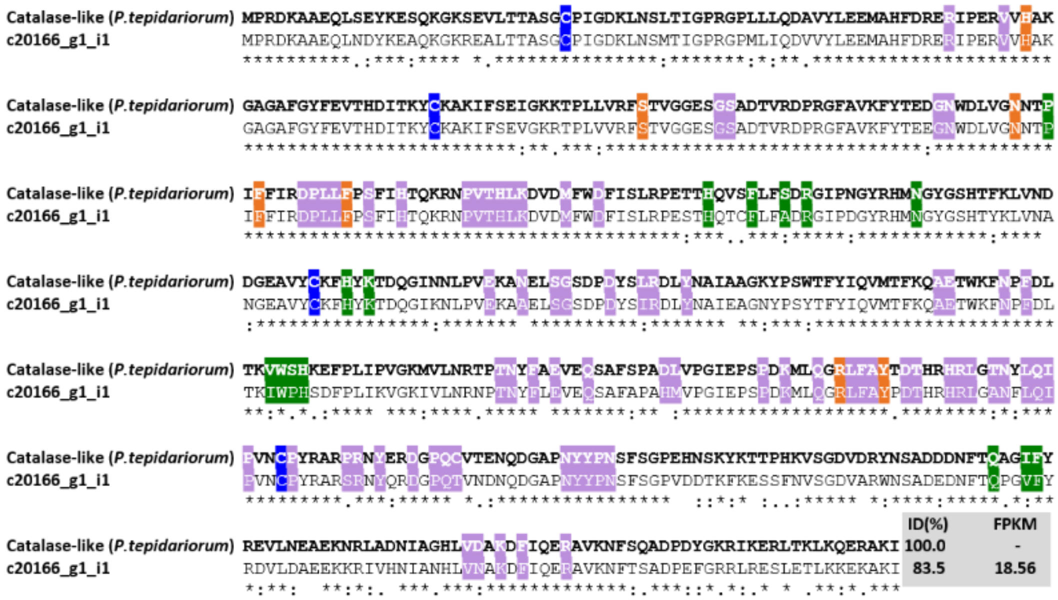


**Fig A41: Sequence alignment of transcripts similar to Catalase.** Alignment was performed with MUSCLE, using catalase-like protein from spider *Parasteatoda tepidariorum* (GenBank XP_015931113.1) as references. Residues that compose the heme-binding pocket are highlighted in orange and residues that bind to NADPH are in green. Residues composing the tetramer interface are highlighted in lavender. Conserved cysteines are highlighted in blue. Percentage of identity (ID%) with the reference proteins was calculated using the tool EMBOSS Stretcher for pairwise sequence alignment. FPKM shows the transcript abundancy.

# GAMMA-GLUTAMYL TRANSPEPTIDASE

```
GGTP-1 (S.mimosarum) --------------------------------------------------------------------------------
c21191_g1_i2         --------------MVNVITPLSSQKFYVNARDE----PRIKRSLTFRAKVSLFVAFVLAAVIVTVVVVCFVLPRKEEEQ
c19667_g1_i1         MSRDGNLTQTVHGSYVQLNTFDDKENEFVQVRTSYPTLLPYKRCLCTKRDTIIVLLVAIGTCILITASLAIALPKIHEKR

GGTP-1 (S.mimosarum) --------------------------------------------------------------M--GFGGGFLM--TVYIR
c21191_g1_i2         EESFWPTYV-SQSKLGRYRHAAVSTDAAPCAPIGKDILERNGTAIDAAIAVLVCMGAHNPHSM--GLGGGFLM--LYYNR
c19667_g1_i1         QQYYDVSFTESSSPLLTYDKAVVVSDGVNCADIGKDILLKGGSAVDAAIAALFCGGVVNPQSMETGLGGGFLMETTVYSR
                                                                                   *  *:******    * *

GGTP-1 (S.mimosarum) ENKTSAVIDARESAPKNADKDM--FKGNSTSSQEGGLSVAVPGELRGYKMAHEKYGKLPWKSLIEPSIKICSDGFEVSKH
c21191_g1_i2         SEERAYYIDAREVAPRKSSVNM--FHGNATHAAEGGLAIAVPGELAGYWEAYRLFGFLPWEELFAPTIEMCRSGIEVSAH
c19667_g1_i1         KTNSSTFIDGRETAPENASKDMETFQGNSSLSVEGGLSIAIPGELRGYWAAHEKFGNLTWKELIEPSIQLCTHGFKVSAH
                      . : :  **.** ** ::. :*  *:**:: : ****::*:**** **. *:  :* *.*:.*: *:*::*  *::** *

GGTP-1 (S.mimosarum) LAKYLQKVKKRILADNSMREEFLNNATNDLYKEGEILKRPVLARTLNEIAEKGADVLY-TGDLHENFLNDISNCGGIITK
c21191_g1_i2         LARGIKNNKEKLKKYEALGKVFLKNGT-DIYEEGDHIKMLDLAKTLEVISEKKMKALYGPSDLADDFLKDLKEAGSIIDR
c19667_g1_i1         LANHLQNYKEKILSDAYLKEEFSNNETNDLYKEGEILKRPNLGITLNEIANKGADVLY-TGDLHETFLKDIRKCGGIITE
                     **. ::: *:.:     : : * :* * *:*:**: :*   *. **: *::*  ..** . .** : **:*: :.*.**

GGTP-1 (S.mimosarum) EDLANYQPKLKPAVKLELEGAEKLFLHSVPPPGSGVILSFILNILSNYNM--TAEDFMDTDNAVLM--YHRITEAFKFAF
c21191_g1_i2         IDMQSYAPRMLDPVKVNLR--RNTTLYGVSPPGSGALLAFMMAVLDGYS---EMDESVVGDEELTALTYHRIVETMKFGY
c19667_g1_i1         TDLANYKPVIKSAVKVNLTTAEALTLHTVPPPGSGLILSLILNILSNYNMETTAKDFKDIDDAVLMETYHRITEAFKFAF
                      *: .* * :  .**::*       *: *.***** :*:::: :*..*.     .:    *: :    ****.*::**.:

GGTP-1 (S.mimosarum) AHRTQLGDDAFVNVTQLVSNLVSREYASSIWRQISD--EKTRPT-SFYKPEM--EVTNDHGTAHVSVVAANGDAVSVTST
c21191_g1_i2         AHRSHMEDDDSQEMEELMEKLTSKEYADHVRGRIND--TRTFDKLSYYGDLW--DYAEDHGTAHLSIIAPNGDAVAVTST
c19667_g1_i1         AERTQLGDEDFVDISELLTKLQSKEYANSLWQQISDKMETTYST-SFYKPIMETETVNDHGTAHVSVLAENGDAVSVTSS
                     * *::: *:   :: :*: :* *.***. :. .*.*    *  . *:*      : .:******:*::* *****:***:

GGTP-1 (S.mimosarum) INTYFGSLCRSPSTGIIILNNAM--DDFSSPNITNYFGIPPSPANYIVPGKRPLSSM--CPTIVVNADGDVHM--VVGGAG
c21191_g1_i2         INQYFGSKILSPSTGIVLNDEM--DDFSSPNITNMFDLPPSAKNNIKPGRRPMSSM--TPMILVDADGEAKL--VLGGNG
c19667_g1_i1         INTYFGSLCLSPSTGIIILNNGMETDDFSSANITNYFGVPPSPANNIVPGKRPLSSMETCPTIAVNADGDVHMETVSGGAG
                     ** ****   ******:**: *  *****.**** *.:***. * * **.**:***   * * *:***:.::  * ** *

GGTP-1 (S.mimosarum) GTRI--ITASALSM--IRTLWLSEDIKRATDAPRFHHQLFPNQIEYEESFPKIYMDKLKG-FGHSVVPETQA--SVFLGI
c21191_g1_i2         GTT---ITSGVLQV-AARSLFFGETIKQSIDAPRIHHQLTPNIMHHEKSFPAALVDKLRK-FGHDAHLWTMGMMSIIMGV
c19667_g1_i1         GTRMETITSSALVMETLRTLWLGQDIKYATDAPRIHHQLFPNVIQYEEGFPQVYQDKLKKEYGHLEQLDDHG--SVFLGI
                     **    **:..* :   *:*::.: ** : ****:**** ** : :*:.**    ***.  :**        .  *:::*:
```

| | Alignment (end) | ID(%) | FPKM |
|---|---|---|---|
| GGTP-1 (S.mimosarum) | IK-ENGKLNTNVDYRKGGSADGF | 100.0 | - |
| c21191_g1_i2 | QRGEDGYLYANSDYRKGGEVAGF | 36.8 | 3.93 |
| c19667_g1_i1 | VR-NGEKLQTNVDYRKGGSVSGY | 54.3 | 3.31 |
| | . :.  * :* ******.. *: | | |

**Fig A42: Sequence alignment of transcripts similar to gamma-glutamyl transpeptidase.** Alignment was performed with MUSCLE, using Gamma-glutamyltranspeptidase 1, from spider *Stegodyphus mimosarum* (UNIPROT A0A087T3V3) as references. Conserved cysteines are highlighted in blue. Percentage of identity (ID%) with the reference proteins was calculated using the tool EMBOSS Stretcher for pairwise sequence alignment. FPKM shows the transcript abundancy.

# TIMP INHIBITORS

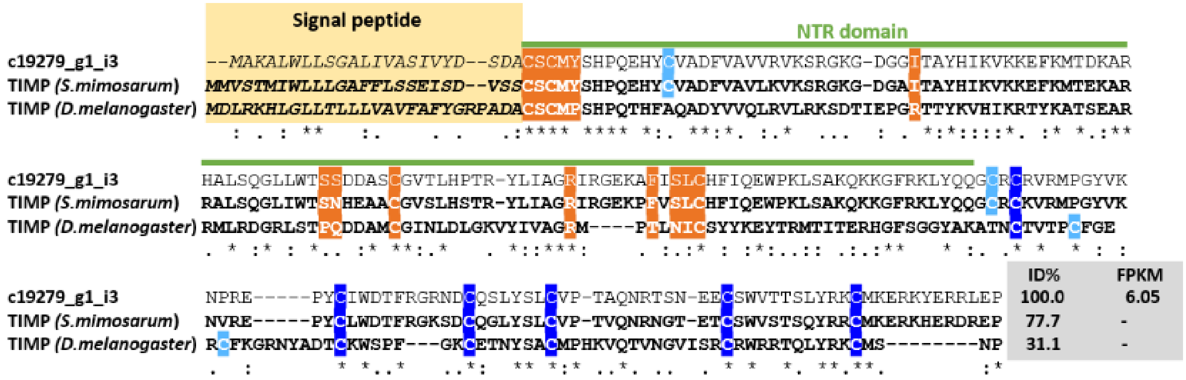


**Fig A43: Sequence alignment of transcripts similar to Tissue metalloproteases inhibitors (TIMP).** Alignment was performed with MUSCLE, using TIMP, from *Drosophyla melanogaster* (UNIPROT Q9VH14) and from spider *Stegodyphus mimosarum* (UNIPROT A0A087TCZ6) as references. Signal peptide is highlighted in yellow. Residues that compose the metzincin binding interface are highlighted in orange. Conserved cysteines are highlighted in blue and non-conserved ones are in cyan. The green line marks the NTR domain. Percentage of identity (ID%) with the reference proteins was calculated using the tool EMBOSS Stretcher for pairwise sequence alignment. FPKM shows the transcript abundancy.
